# Transcriptional regulation of metal metabolism- and nutrient absorption-related genes in *Eucalyptus grandis* by arbuscular mycorrhizal fungi at different zinc concentrations

**DOI:** 10.1186/s12870-022-03456-5

**Published:** 2022-02-22

**Authors:** Xinyang Wang, Jingwei Liang, Ziyi Liu, Yuxuan Kuang, Lina Han, Hui Chen, Xianan Xie, Wentao Hu, Ming Tang

**Affiliations:** State Key Laboratory of Conservation and Utilization of Subtropical Agro-bioresources, Guangdong Laboratory for Lingnan Modern Agriculture, Guangdong Key Laboratory for Innovative Development and Utilization of Forest Plant Germplasm, College of Forestry and Landscape Architecture, South China Agricultural University, Guangzhou, 510642 China

**Keywords:** *Eucalyptus grandis*, Zinc stress, Arbuscular mycorrhizal fungi, Genes transcript level

## Abstract

**Background:**

*Eucalyptus* spp. are candidates for phytoremediation in heavy metal (HM)-polluted soils as they can adapt to harsh environments, grow rapidly, and have good economic value. Arbuscular mycorrhizal fungi (AMF) are the most widely distributed plant symbiotic fungi in nature, and they play an important role in promoting the phytoremediation of HM-polluted soils. However, few studies have evaluated the HM detoxification mechanism of *E.* spp. in symbiosis with AMF, and thus, the molecular mechanism remains unclear.

**Results:**

The gene transcription and metabolic pathways of *E*. *grandis* were studied with and without inoculation with AMF and at different zinc (Zn) concentrations. Here, we focused on the transcript level of six HM-related gene families (ZNT, COPT/Ctr, YSL, ZIFL and CE). Under high-Zn conditions, thirteen genes (ZNT:2, COPT/Ctr:5, YSL:3, ZIFL:1, CE:2) were upregulated, whereas ten genes (ZNT:3, COPT/Ctr:2, YSL:3, ZIFL:1, CE:1) were downregulated. With AMF symbiosis under high-Zn conditions, ten genes (ZNT:4, COPT/Ctr:2, YSL:3, CE:1) were upregulated, whereas nineteen genes (ZNT:9, COPT/Ctr:2, YSL:3, ZIFL:4, CE:1) were downregulated. Under high-Zn conditions, genes of three potassium-related transporters, six phosphate transporters (PHTs), and two nitrate transporters (NRTs) were upregulated, whereas genes of four potassium-related transporters,four PHTs, and four nitrogen-related transporters were downregulated. With AMF symbiosis under high-Zn conditions, genes of two potassium-related transporters, six ammonium transporters (AMTs) and five PHTs were upregulated, whereas genes of six potassium-related transporters, two AMTs and five PHTs were downregulated.

**Conclusions:**

Our results indicates that AMF increases the resistance of *E*. *grandis* to high-Zn stress by improving nutrients uptake and regulating Zn uptake at the gene transcription level. Meanwhile, our findings provide a genome-level resource for the functional assignments of key genes regulated by Zn treatment and AM symbiosis in six HM-associated gene families and macromineral nutrient-related gene families of *E*. *grandis*. This may contribute to the elucidation of the molecular mechanisms of the response to Zn stress in *E*. *grandis* with AM symbiosis at the aspect of the interaction between HM tolerance and nutrient acquisition.

**Supplementary Information:**

The online version contains supplementary material available at 10.1186/s12870-022-03456-5.

## Background

Large quantities of heavy metals (HMs), such as zinc (Zn), copper (Cu), iron (Fe), and cadmium (Cd), are often released into the soil as a result of soil acidity, flooding, mining, industrial activities, or other sources of pollution [1]. HM contamination reduces the area of usable land and restricts the distribution of vegetation. Moreover, in tailings areas, the bioaccumulation of heavy metals in plants and animals causes serious harm to the food chain and, consequently, human health. To eliminate HMs from the ecosystem, bioremediation and phytoremediation are the most significant and appropriate strategies [2]. Phytoextraction and phytostabilization are the main methods used for phytoremediation [3]. Many plants have been developed for phytoextraction and phytostabilization. For example, Brown et al. [4] discovered that *Thlaspi caerulescens* super-accumulates Zn and Cd, indicating that the plant has potential remediation capacity in HM-contaminated soil. Blaylock et al. [5] found that mustard greens (*Brassica juncea*) can absorb and accumulate various HMs, such as lead (Pb), Cd, and Zn, and that *Thlaspi* can effectively absorb Zn, Pd, and Cd. However, many of the plants currently used for the bioremediation of HM pollution have slow growth and low biomass content [6, 7]. Therefore, to improve remediation efficiency, a feasible direction for the bioremediation of HM-contaminated soil is to screen for plants that are fast-growing, have high biomass content, and are HM tolerant.

*Eucalyptus grandis* has the characteristics of fast growth, strong stress resistance, and high economic value [8]. Both *E. grandis* 5 and *E. grandis* × *E. uroplrlla* can adapt to severe environments in mining areas and also have strong phytoremediation potential [9]. In some mining areas polluted with Zn, *Eucalyptus* spp*.* have been shown to be HM pollution hyperaccumulators [10]. In the Huangpu District of Guangzhou, where Zn pollution is a serious problem, *E. urophylla* became the dominant species [11]. These data suggest that *Eucalyptus* spp. are potential tools to remedy Zn pollution in the soil [12]. Moreover, *Eucalyptus* spp. can establish symbioses with arbuscular mycorrhizal fungi (AMF) and ectomycorrhizal (ECM) fungi [13]. Campos et al. [14] found AMF/ECM fungi symbioses at all ages of *E. grandis* and *E. urophylla*.

Zn can be toxic if it accumulates in excess amounts, although it is also an essential nutrient for plant growth at low concentrations [15]. Zn toxicity often occurs in soils contaminated by mining and smelting activities, in agricultural soils treated with sewage sludge, and in urban soils enriched with anthropogenic Zn inputs [16]. To respond to Zn toxicity, plants require a tightly controlled metal homeostasis network that balances the uptake, utilization, and tolerance of Zn [17, 18]. Zn-related transporters are involved in the absorption, intracellular transport, localization, and efflux of Zn. These include the P1B-type heavy metal ATPase (HMA), Zn- and Fe-regulated transporter-like protein (ZIP), copper transporter (COPT/Ctr), yellow stripe-like (YSL) transporter, Zn-induced facilitator1 (ZIF1) transporter, and cation efflux (CE) transporter families.

There is convincing evidence that mycorrhizal associations are of major importance in reducing metal transfer to plants [19] or serving as an effective exclusion barrier against the transport of these elements from roots to shoots [20]. In addition, mycorrhizal fungi, especially AMF, favor the absorption of nitrogen (N), phosphorus (P), and potassium (K) [21]. AMF also improve the tolerance of plants to HM stress [22], prevent plant contact with HMs, and favor the extraction of HMs from the soil [23]. As many plants form AMF symbioses, even in highly HM-contaminated soils [24], AMF may be an important tool for the remediation of HM-contaminated soils [25]. Inoculation with AMF can improve the nutritional status of *Eucalyptus* spp*.* by increasing N, P, and K concentrations and improve the tolerance of *Eucalyptus* spp. to HM stress [26]. Previous studies have shown that under conditions of Pb stress, AMF symbiosis can increase the uptake of Pb by the shoots of *E. globulus* [27]. However, few studies have focused on the mechanism of Zn tolerance and enrichment in *Eucalyptus* spp. with AMF symbiosis. It is necessary to explore the detoxification mechanism of *Eucalyptus* spp. in response to Zn stress, as this may provide the theoretical basis for the ecological restoration of artificial *E. grandis* forests in Zn-contaminated areas.

In this study, to better understand the mechanisms of the response to Zn stress in *E. grandis* with AMF symbiosis, candidate genes were screened using RNA sequencing (RNA-seq) analysis of *E. grandis* under different zinc chloride (ZnCl_2_) concentrations. Important response pathways, genes involved in Zn tolerance, and gene families associated with nutrient absorption were identified. This aim of this study was to determine (i) the type of metal transporters that are regulated by different Zn concentrations and (ii) the type of HM transporters and nutrient-associated transporters that respond to Zn treatment with AMF symbiosis. These results may contribute to a greater understanding of Zn-responsive mechanisms in *E. grandis* with AMF symbiosis.

## Results and discussion

In this study, we investigated genes involved in Zn and nutrient absorption by the transcriptome analysis of *E. grandis* with AMF symbiosis under different Zn concentrations, followed by the putative subcellular localization and functional prediction of DEGs. Correlations among genes belonging to the same gene family were also analyzed. After inoculation with AMF under high-Zn conditions, 2253 DEGs were identified, of which 1683 were upregulated and 570 were downregulated. However, only 539 DEGs were identified under high-Zn conditions without AMF inoculation, of which 275 were upregulated and 264 were downregulated (Supplementary Fig. S1). These findings represent the first step in determining the molecular mechanisms of the response of *E. grandis* to high-Zn stress and provide plenty of new candidate genes for studies on tolerant germplasm development and molecular biology in woody plants.

### Translocation and accumulation of Zn in mycorrhizal *Eucalyptus*

There were no obvious symptoms of visual toxicity, regardless of metal addition or AMF inoculation. Because the metal treatment was performed after the inoculation, the colonization rate did not change significantly, but the arbuscule abundance decreased significantly and the number of hyphae increased under high-Zn conditions (Supplementary Fig. S2).

Compared with the normal Zn concentration, the high Zn concentration caused a 35% reduction in the Pi content of the shoots of *E. grandis*, but the Pi content of the roots did not change significantly (Supplementary Fig. S3). After inoculation with AMF, the Pi content of the shoots increased, indicating that AMF increased the Pi content of *E. grandis* (Supplementary Fig. S3). The pre-experiment shows that *E. grandis* can promote plant growth after inoculation with AMF (Supplementary Fig. S4). These results also indicate that there is an antagonistic relationship between Zn and P in *Eucalyptus* spp. and that mycorrhizal plants may reduce HM-induced damage to plants through P absorption. The interconnection between these two nutrients has been observed in many crop species and can be summarized as follows: Zn-deficient plants over-accumulate Pi in the shoots and, conversely, Pi-deficient plants overaccumulate Zn in the shoots [28]. Mycorrhizae play an important role in the acquisition of P by the host plant [29], and they may also facilitate Zn transport in the soil-fungi-plant continuum [19]. Nevertheless, the molecular basis of the Pi-Zn interaction in plants remains poorly understood in both mycorrhizal and non-mycorrhizal plants [30].

### Transporters that introduce metals into the cytosol

As seedlings from the NM- and AMF-treatment groups demonstrated distinct patterns of Zn and P contents in their acclimation to Zn availability (Supplementary Fig. S3), specific differences may also be expected in the transcriptional regulation pattern of key genes implicated in Zn metabolism [31]. Therefore, the transcript levels of representative genes involved in Zn acquisition and assimilation were assessed in the roots of plants in the NM- and AMF-treatment groups.

### ZNT family

ZNTs are micronutrient transporters from the zinc-resistance transporter and iron-resistance transporter-like protein (ZIP) family. ZIP transporters contain a conserved cytosolic histidine-rich loop between transmembrane (TM) domains 3 and 4 in eukaryotes [32], which is associated with metal specificity and metal transport rate [33].

Nineteen candidate ZNT family genes were identified in the *E. grandis* genome. The characteristics of these genes, including the open reading frame length, chromosome location, number of exons, and the molecular weight and isoelectric point of the encoded protein were analyzed and are shown in Supplementary Table 1. To identify the evolutionary relationships between *EgZNT* genes of different plant species, a phylogenetic tree was constructed with 86 ZIP transport protein sequences from 10 plant species (Supplementary Fig. S5). Bioinformatics analyses showed that ZNT genes were well conserved and had similar physicochemical properties. Conserved motif structures were also detected (Supplementary Fig. S6). And the chromosomal location of ZNT gene family were shown in the chromosome-scale genome of *E.grandis* (Supplementary Fig. S7).

**Fig. 1:**
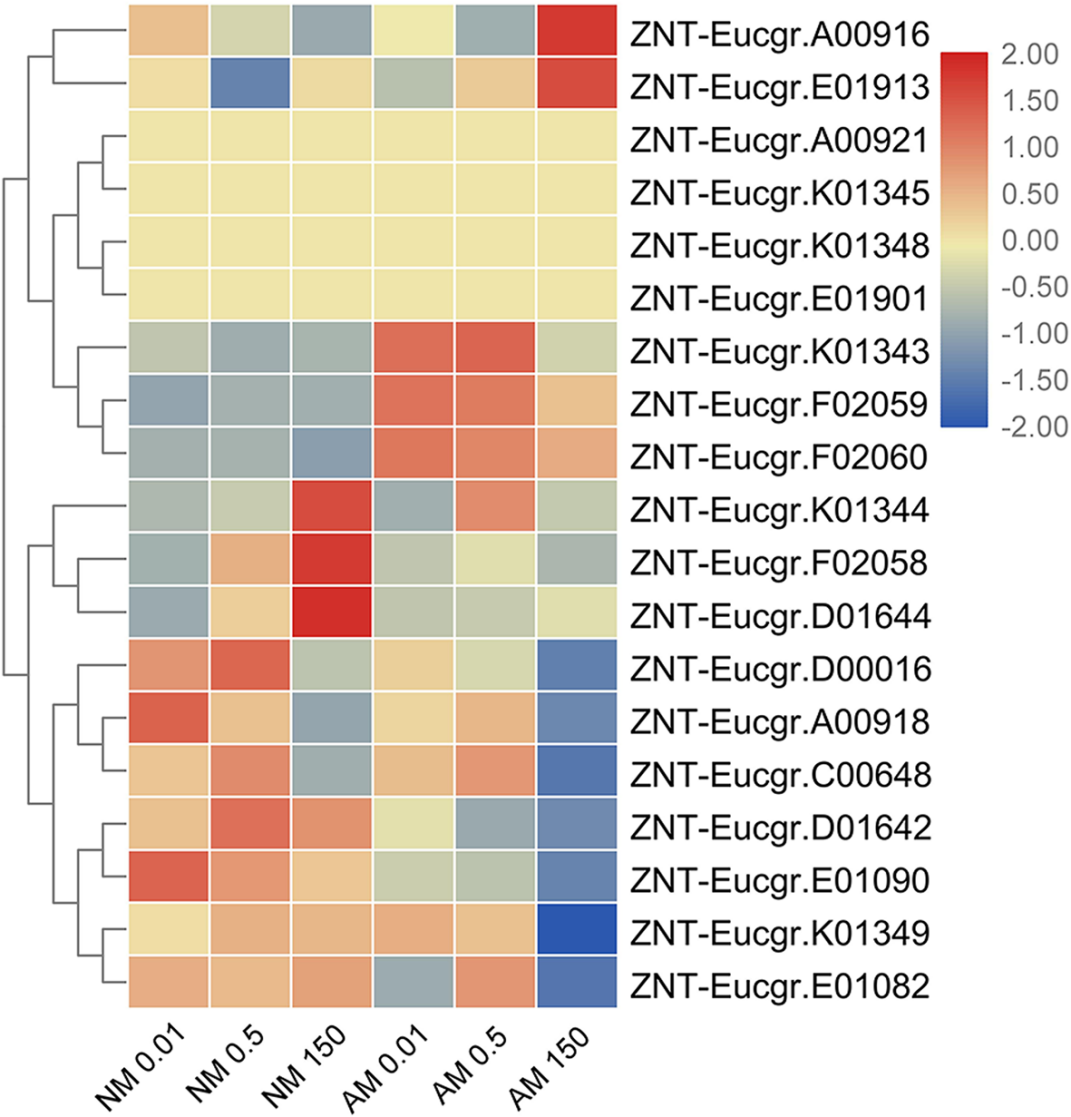
Heatmap of ZNT family members. Cluster analysis of transcriptional fold-changes of ZNT family gens in roots of non-mycorrhizal and mycorrhizal *E. grandis* exposed to 0.01, 0.5, or 150 μM ZnCl_2_. The color scale indicates fold-changes of mRNAs. For each gene, the expression levels in non-mycorrhizal roots exposed to 0.5 μM ZnCl_2_ were defined as 1, and the corresponding fold-changes under 0.01 and 150 μM ZnCl_2_ were calculated

Cluster analysis of transcript levels of the ZNT gene family clearly separated root samples from NM- and AMF-treatment groups based on their responsiveness to Zn (Fig. 1). Nine ZNT genes (*ZNT-Eucgr.A00916*, *ZNT-Eucgr.E0191*3, *ZNT-Eucgr.A00921*, *ZNT-Eucgr.K01345*, *ZNT-Eucgr.K01348*, *ZNT-Eucgr.E01901*, *ZNT-Eucgr.K01343*, *ZNT-Eucgr.F02059*, and *ZNT-Eucgr.F02060*) formed subcluster I. Under high-Zn conditions, the abundance of the transcripts of subcluster I genes in roots from the AMF-treatment group was higher than or similar to that in roots from the NM-treatment group. The second subcluster of genes included *ZNT-Eucgr.K01344*, *ZNT-Eucgr.F02058*, *ZNT-Eucgr.D01644*, *ZNT-Eucgr.A00918*, *ZNT-Eucgr.C00648*, *ZNT-Eucgr.D01642*, *ZNT-Eucgr.E01090*, *ZNT-Eucgr.K01349*, and *ZNT-Eucgr.E01082*. The transcript levels of these genes were lower in roots from the AMF-treatment group than in roots from the NM-treatment group under low-Zn conditions.

Limiting the Zn supply affected the transcript levels of genes involved in Zn metabolism in the roots from both the AMF- and NM-treatment groups. Generally, the transcript levels of genes from subcluster I were higher in roots from the AMF-treatment group than in those from the NM-treatment group under low-Zn conditions (Fig. 1). However, the transcriptional pattern of *ZNT-Eucgr.E01913* was completely different between the NM- and AMF-treatment groups. The transcript levels of this gene were upregulated in roots from the NM-treatment group under low- and high-Zn conditions, whereas in roots from the AMF-treatment group, they were only upregulated under high-Zn conditions. We found that ZNT-Eucgr.E01913, OsIRT1, HvIRT1, AtIRT1, MtZIP1, and MtZIP6 were in the same branch of the phylogenetic tree (Supplementary Fig. S5). *HvIRT1* is involved in Zn absorption under conditions of Zn deficiency [34]. *IRT1* is considered to be the main transporter for Fe^2+^ uptake in roots and is polarly localized to the soil-facing side of the epidermal plasma membrane [35]. *AtIRT1* is involved in Fe uptake. Barberon et al. [35] showed that plants growing under high Zn concentrations are deficient in iron. Recently, a ZIP transporter gene (*MtZIP6*) in the model legume *Medicago truncatula* was identified as being significantly upregulated by AMF colonization when Zn was at a limiting concentration [36]. The ZIP transporter encoded by *MtZIP6* is located in the plasma membrane and is involved in Zn transport in rhizobial symbioses [37]. However, *MtZIP6* has been shown to transport Fe in addition to Zn [38]. In contrast, the lower affinity of *MtZIP1* for Zn suggests that this transporter plays a role in Zn transport within the plant, because the concentration of Zn has been shown to be much higher in plant compartments than in the soil [39]. Therefore, the increase in the expression levels of *ZNT-Eucgr.E01913* under low-Zn conditions suggested that it is also involved in iron absorption under conditions of Zn deficiency. The increase in the expression levels of *ZNT-Eucgr.E01913* under high-Zn conditions may be caused by iron deficiency. This result also suggests that AMF may assist plants to absorb Zn through other mechanisms at low Zn concentrations. Thus, *ZNT-Eucgr.E01913* may be involved in regulating the zinc-iron balance in plant roots.

The expression of *ZNT-Eucgr.K01343*, which is homologous to *AtZIP1* (Supplementary Fig. S5), from the ZIP family, was induced under conditions of Zn deficiency and was inhibited under high-Zn conditions (Fig. 1). *AtZIP1* serves as a vacuolar transporter that remobilizes Zn from the vacuole to the cytoplasm in root cells [40]. *AtZIP1* expression is mainly induced under conditions of Zn deficiency and is involved in Zn uptake and redistribution [41]. Thus, *ZNT-Eucgr.K01 343*may also be involved in Zn uptake and redistribution under conditions of Zn deficiency.

ZNT-Eucgr.F02059 and ZNT-Eucgr.F02060 have a high degree of homology and are in the unified branch of the phylogenetic tree with OsZIP2, MtZIP7, and AtZIP2(Supplementary Fig. S5). Milner et al. [40] reported that Zn uptake from the root apoplast is carried out by *AtZIP2* in the root endodermis of *Arabidopsis thaliana*. *AtZIP2* is a Zn uptake transporter that is located mainly in the root stele. The expression levels of *ZNT-Eucgr.F02059* and *ZNT-Eucgr.F02060* were low under NM conditions and were not affected by the Zn concentration. The levels of these two transcripts increased significantly after AMF inoculation, but their levels decreased with the increase in Zn concentration with AM treatment. *MtZIP7*, a putative Zn and manganese (Mn) transporter, is expressed in both arbuscule-colonized and adjacent non-colonized cortical cells [42]. Our results showed that the expression of *ZNT-Eucgr.F02059* and *ZNT-Eucgr.F02060* is specifically induced by mycorrhizae. AMF contribute to Zn uptake by their host plants and alleviate HM toxicity. Therefore, *ZNT-Eucgr.F02059* and *ZNT-Eucgr.F02060* were specifically induced by AMF symbiosis, and their encoded proteins may transport Zn released by the AMF at the symbiotic interface.

Of the ZNTs in subcluster II, the transcript abundance of *ZNT-Eucgr.D01642*, *ZNT-Eucgr.D01644*, *ZNT-Eucgr.E01082*, and *ZNT-Eucgr.E01090* decreased after inoculation with AMF under high-Zn conditions (Fig. 1). These genes are in the same branch of the phylogenetic tree and are highly homologous, but only *ZNT-Eucgr.D01644* was induced by a high Zn concentration without AMF. These four transporters are in the same subfamily as *MtZIP2* and *AsZIP2* in the phylogenetic tree (Supplementary Fig. S5) The expression level of *AsZIP2* has been shown to increase with increasing Zn concentration [43]. *ZNT-Eucgr.D01644* may have the same function as *MtZIP2* to transport excess Zn from the intercellular space into the vacuole and thus maintain a suitable cytoplasmic Zn concentration [44]. As shown in Fig. 1, AMF symbiosis effectively inhibited the transcript levels of this subfamily, presumably because AMF symbiosis inhibits excessive Zn absorption [44]. There are two possible explanations for this phenomenon. First, AM fungal hyphae bind and fix HMs on the fungal cell wall, thus reducing the absorption of heavy metals by the host plant. Second, AMF deposit and chelate HMs in the rhizosphere [45].

We performed Pearson’s correlation analysis of the ZNT family genes and found that the transcript levels of *ZNT-Eucgr.K01343* and *ZNT-Eucgr.F02060* (*R* = 0.739), *ZNT-Eucgr.F02059* and *ZNT-Eucgr.F02060* (*R* = 0.953), *ZNT-Eucgr.D01644* and *ZNT-Eucgr.F02058* (*R* = 0.765), and *ZNT-Eucgr.K01344* and *ZNT-Eucgr.E01901* (*R* = 0.78) were highly significantly correlated (*P < 0.001*, Supplementary Table. S2).

### COPT/Ctr transporters

Cu transporters are known as COPTs in plants [46] and Ctrs in animals and fungi [47]. In plants, COPTs have been suggested to play a role in Cu uptake from the soil and Cu delivery to pollen [48]. The *PtCOPT* gene of *Populus trichocarpa* is expressed in many tissues and may be involved in regulating plant development [49]. Previous studies have indicated that the COPT/Ctr gene family in animals and herbaceous plants is induced during deficiencies and excesses of Cu [50] and by Fe, Mn, and Zn stress [51].

**Fig. 2:**
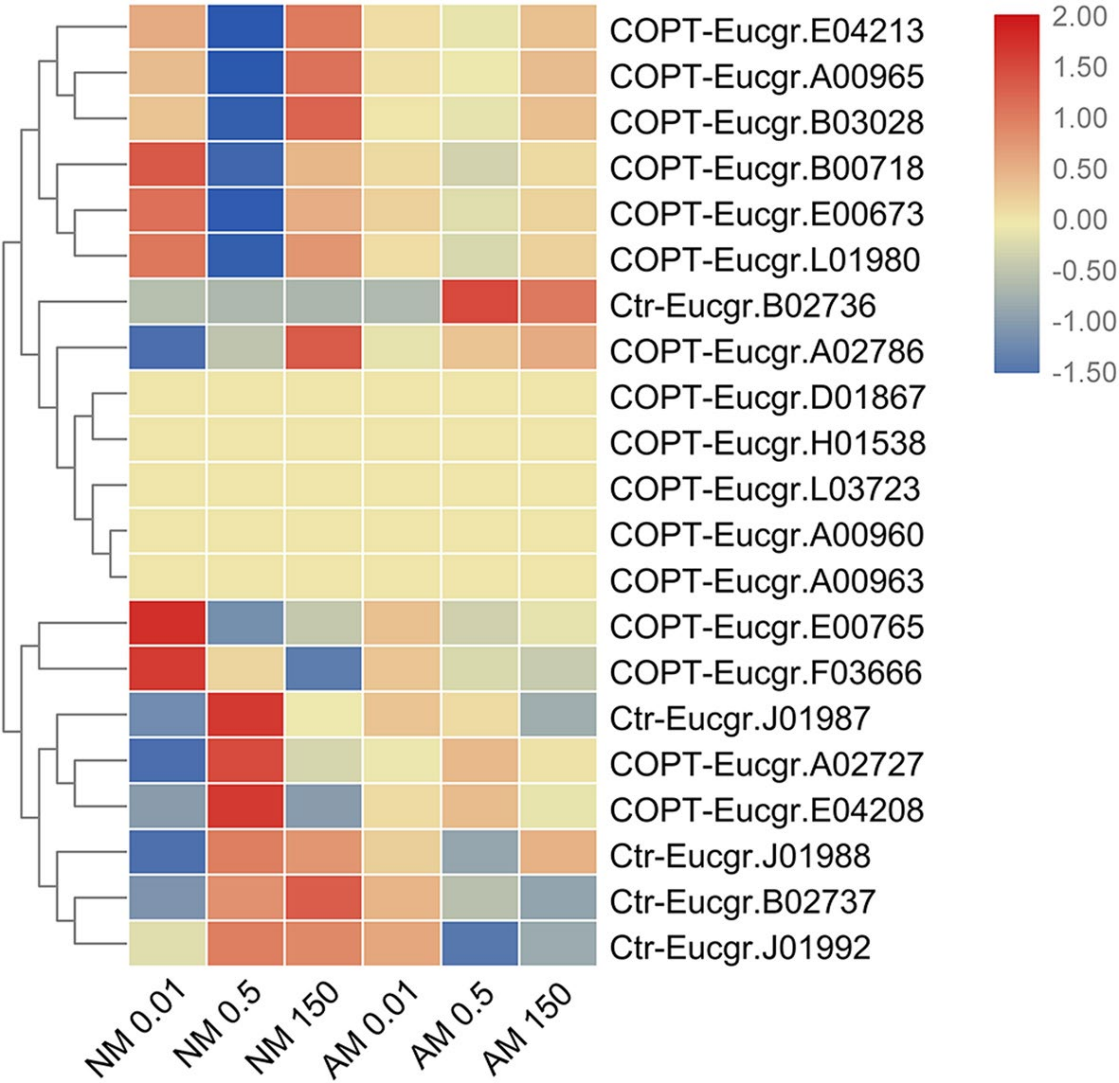
Heatmap of COPT/Ctr family members. Cluster analysis of transcriptional fold-changes of COPT/Ctr family gens in roots of non-mycorrhizal and mycorrhizal *E. grandis* exposed to 0.01, 0.5, or 150 μM ZnCl_2_. The color scale indicates fold-changes of mRNAs. For each gene, the expression levels in non-mycorrhizal roots exposed to 0.5 μM ZnCl_2_ were defined as 1, and the corresponding fold-changes under 0.01 and 150 μM ZnCl_2_ were calculated

Twenty-one candidate COPT/Ctr family genes were identified in the *E. grandis* genome. Heat maps showed that COPTs were divided into two subclusters (Fig. 2). In roots, *COPT-Eucgr.E04213*, *COPT-Eucgr.A00965*, *COPT-Eucgr.B03028*, *COPT-Eucgr.00718*, *COPT-Eucgr.E00673*, *COPT-Eucgr.L01980*, *Ctr-Eucgr.B02736*, *COPT-Eucgr.A02786*, *COPT-Eucgr.D01867*, *COPT-Eucgr.H01538*, *COPT-Eucgr.L03723*, *COPT-Eucgr.A00960*, and *COPT-Eucgr.A00963* formed subcluster I (Fig. 2). Subcluster I was divided into two small clusters. The transcript abundance of *COPT-Eucgr.E04213*, *COPT-Eucgr.A00965*, *COPT-Eucgr.B03028*, *COPT-Eucgr.00718*, *COPT-Eucgr.E00673*, and *COPT-Eucgr.L01980* in subcluster I was higher under low- and high-Zn conditions in both the NM- and AMF-treatment groups compared with their levels under normal-Zn condition. The expression levels of genes in the other cluster were diverse. For example, the expression level of *Ctr-Eucgr.B02736* induced by AMF increased significantly under both low- and high-Zn conditions, and its expression level under high-Zn conditions decreased after NM and AMF treatments.

The second subcluster consisted of *COPT-Eucgr.E00765*, *COPT-Eucgr.03666*, *Ctr-Eucgr.J01987*, *COPT-Eucgr.A02727*, *COPT-Eucgr.E04208*, *Ctr-Eucgr.J01988*, *COPT-Eucgr.B02737*, and *Ctr-Eucgr.J01992*. The transcript levels of these genes, except *COPT-Eucgr.E00765* and *COPT-Eucgr.03666*, were similar or lower after NM and AMF treatments under low-Zn conditions compared with their levels under normal-Zn conditions (Fig. 2).

Most members of the COPT gene family of *E. grandis* were in the same branch of the phylogenetic tree, indicating that the genes of this family are highly conserved in *E. grandis* (Supplementary Fig. S8).

Based on a comparison of transcriptome and JGI data, two groups of Ctrs and COPTs were identified in *E. grandis*. Sixteen of these genes were classified as COPTs and five were classified as Ctrs (Fig. 2). The five Ctr transporters had a high degree of homology with the previously reported *PtCOPT* gene of *P. trichocarpa*. In all COPT/Ctr transporters, motif 1 and motif 3 corresponded to the TM sites. It is noteworthy that motif 1 contained both the MxxxM and GxxxG motifs (Supplementary Fig. S9). The MxxxM motif is essential for Cu acquisition [52], and the conserved GxxxG motif is essential for trimerization in hCtr1 [53]. Previous studies of COPT/Ctr families in animals and herbaceous plants have demonstrated their upregulation during deficiencies and excesses of Cu [50] and in response to Fe, Mn, and Zn stress [51]. And the chromosomal location of COPT/Ctr gene family were shown in the chromosome-scale genome of *E.grandis* (Supplementary Fig. S10).

Heatmap analysis revealed that the expression of *EgCOPT* genes was induced under both limited and excessive Zn concentrations (Fig. 2). This result is consistent with the *PtCOPT* expression pattern previously identified in *P. tomentosa* [49]. Quantitative reverse transcription-PCR analysis has shown that the expression of *PtCOPT* genes is induced under conditions of limited and excessive Zn [49]. After inoculation with AMF, under low-Zn conditions, EgCOPT promoted the absorption of Zn and increased the Zn concentration in the plant, resulting in decreased expression levels of *EgCOPT*s. Under high-Zn conditions, mycorrhizal fungi can absorb excess Zn, reduce the Zn concentration in the plant, and reduce the expression level of *EgCOPT* (Fig. 2). This result indicates that mycorrhizal fungi can maintain the balance between HM ions in the host plant, thereby reducing the impact of HM ions. Under Zn stress, mycorrhizal fungi have been shown to downregulate plant Zn transporters to promote homeostasis [54].

We performed Pearson’s correlation analysis of the COPT/Ctr family genes and found that the transcript levels of *COPT-Eucgr.A00965* and *COPT-Eucgr.E00673* (*R* = 0.772) and *COPT-Eucgr.E00765* and *COPT-Eucgr.E04213* (*R* = 0.748) were highly significantly correlated (*P* < 0.001, Supplementary Table. S3).

### YSL transporter family

YSL family transporters belong to the oligopeptide transporter family and are significant iron transport proteins. YSL transporters do not use free metals as substrates, but complexes of metals with nicotiana amine (NA) or its derivatives [55]. NA is a non-proteogenic amino acid that is synthesized from S-adenosyl-methionine by the enzyme NA synthase [56](NAS). Transport by YSL proteins is induced by H^+^-symport [57]. Additionally, at least some plant oligonucleotide transporters are also associated with metal transport [58], although the identity of the metal complex transported remains elusive. Little is known about the structure of these proteins, with different models proposing a range of 11–16 TM regions [58]. In broad terms, YSL transporters are involved in metal uptake from the soil in monocots and in long-distance metal distribution in both monocots and dicots [59].

**Fig. 3:**
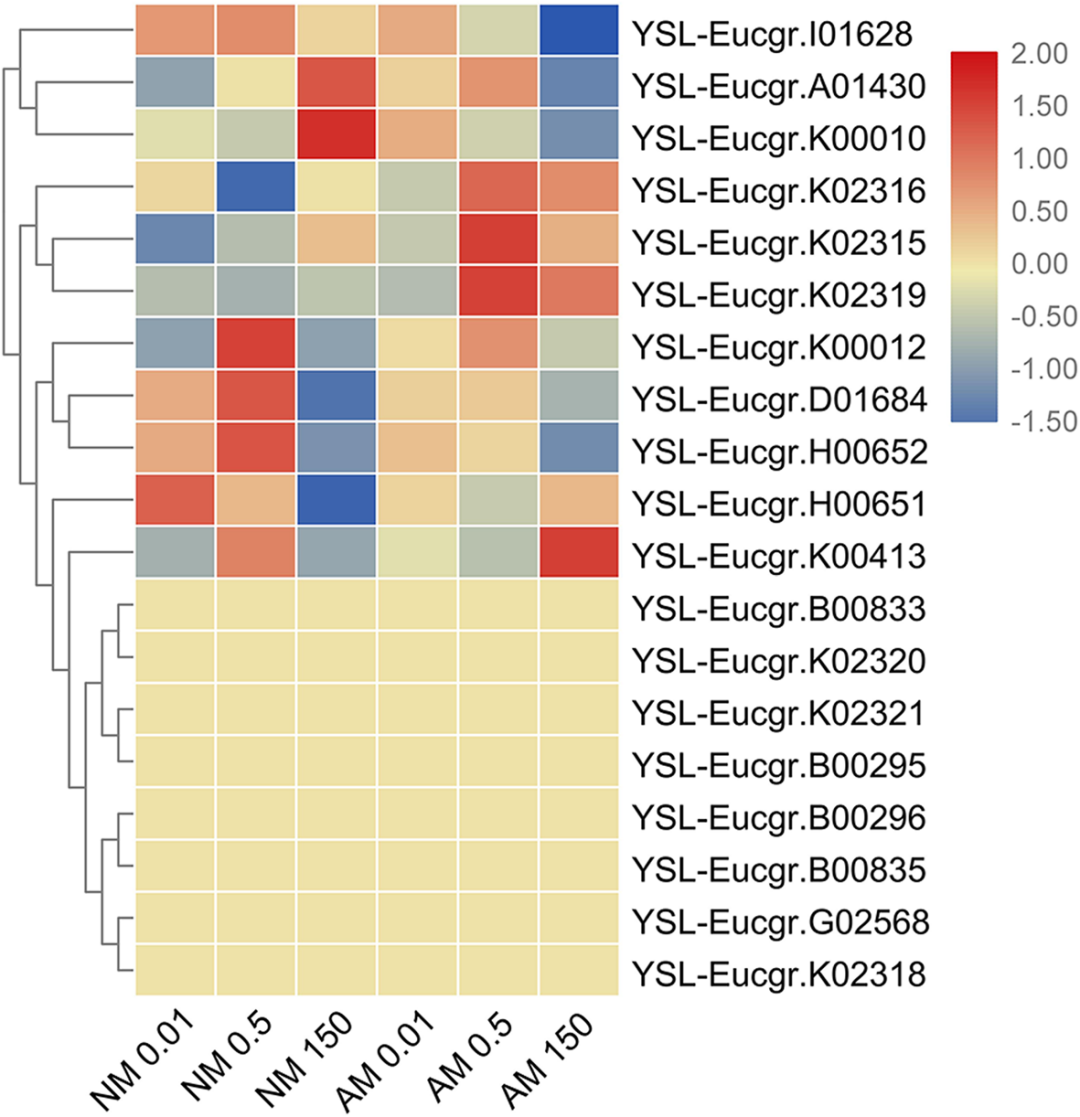
Heatmap of YSL family members. Heatmap of YSL family members. Cluster analysis of transcriptional fold-changes of COPT/Ctr family gens in roots of non-mycorrhizal and mycorrhizal *E. grandis* exposed to 0.01, 0.5, or 150 μM ZnCl_2_. The color scale indicates fold-changes of mRNAs. For each gene, the expression levels in non-mycorrhizal roots exposed to 0.5 μM ZnCl_2_ were defined as 1, and the corresponding fold-changes under 0.01 and 150 μM ZnCl_2_ were calculated

Nineteen candidate YSL family genes were identified in the *E. grandis* genome. Limiting the supply of Zn affected the transcript levels of genes involved in Zn metabolism in the roots of both AMF- and NM-treatment groups. In roots, *YSL-Eucgr.I01628*, *YSL-Eucgr.A01430*, and *YSL-Eucgr.K00010* formed subcluster I (Fig. 3). Under high-Zn conditions, the transcript abundance of genes in subcluster I was higher after NM treatment than after AMF treatment (Fig. 3). The second subcluster consisted of *YSL-Eucgr.K02316*, *YSL-Eucgr.K02315*, *YSL-Eucgr.K02319*, *YSL-Eucgr.K00012*, *YSL-Eucgr.D01684*, *YSL-Eucgr.H00652*, *YSL-Eucgr.H00651*, *YSL-Eucgr.K00413*, *YSL-Eucgr.B00833*, *YSL-Eucgr.K02320*, *YSL-Eucgr.K02321*, *YSL-Eucgr.B00295*, *YSL-Eucgr.B00296*, *YSL-Eucgr.B00835*, *YSL-Eucgr.G02568*, and *YSL-Eucgr.K02318.* Under high-Zn conditions, the transcript levels of these genes were similar or lower after NM treatment compared with their levels after AMF treatment.

To identify the evolutionary relationships between *EgYSL* genes of different plant species, a phylogenetic tree was constructed with 50 YSL transport protein sequences from 5 plant species (Supplementary Fig. S11). Bioinformatics analyses showed that YSL genes were well conserved and had similar physicochemical properties. Conserved motif structures were also detected (Supplementary Fig. S12). And the chromosomal location of YSL gene family were shown in the chromosome-scale genome of *E.grandis* (Supplementary Fig. S13).

We found that the expression level of *YSL-Eucgr.K00010* was significantly increased under high-Zn conditions without AMF inoculation but significantly decreased under high-Zn conditions after with AMF inoculation (Fig. 3). Comparatively, *YSL-Eucgr.K00010* expression levels increased after inoculation with AMF under low-Zn conditions. This result showed that after inoculation with AMF, the expression of this gene can be promoted when Zn concentrations are low and inhibited when Zn concentrations are high. Therefore, this gene may play a role in both nutrient absorption and metal resistance.

The expression level of *YSL-Eucgr.K02319* increased significantly after inoculation with AMF under normal- and high-Zn conditions, and its expression levels decreased after both NM and AMF treatments under high-Zn conditions. We found that YSL-Eucgr.K02319 was in the same branch of the phylogenetic tree as TcYSL5 and TcYSL7 (Supplementary Fig. S11). In situ hybridization showed that *TcYSL7* and *TcYSL5* are expressed around the vasculature of the shoots and in the central cylinder in the root [60]. Exposure to HMs (Zn, Cd, and nickel (Ni)) does not affect the high levels of constitutive expression of *TcYSL* genes [60] (Gendre et al., 2007). We found that *YSL-Eucgr.K02319* was induced by AMF and was affected by changes in the Zn concentration, indicating that this gene may be involved in the transport of HM and homeostatic balance.

We performed Pearson’s correlation analysis of genes in the YSL family and found that the transcript levels of *YSL-Eucgr.K02319* and *YSL-Eucgr.K02321* were highly significantly correlated (*R* = 0.924, *P < 0.001*; Supplementary Table. S4).

### ZIFL transporter family

ZIFL genes are major facilitator superfamily (MFS) transporters that play roles in responding to different stresses, including Zn stress [61]. Since the identification of three *ZIFL* genes in *Arabidopsis*, referred to as *AtZIF1* (AT5G13740), *AtZIFL1* (AT5G13750), and *AtZIFL2* (AT3G43790), evidence for their role in Zn homeostasis has been accumulating [61]. ZIF1 is thought to be involved in the proton-coupled transport of metal chelators or metal-chelate complexes into vacuoles, as the ZIF1 protein contains conserved motifs for proton/substrate antiport and related proteins mostly transport organic molecules [62].

Eight candidate ZIFL family genes were identified in the *E. grandis* genome. To identify the evolutionary relationships between *EgZIFL* genes from different plant species, a phylogenetic tree was constructed with 35 ZIFL transport protein sequences from five plant species (Supplementary Fig. S14). Bioinformatics analyses showed that ZIFL genes were well conserved and had similar physicochemical properties. Conserved motif structures were also detected (Supplementary Fig. S15). And the chromosomal location of ZIFL gene family were shown in the chromosome-scale genome of *E.grandis* (Supplementary Fig. S16).

*ZIFL* genes were found to have different evolutionary histories in monocot and dicot lineages, which is consistent with the conclusions of Ricachenevsky et al. [63].

**Fig. 4:**
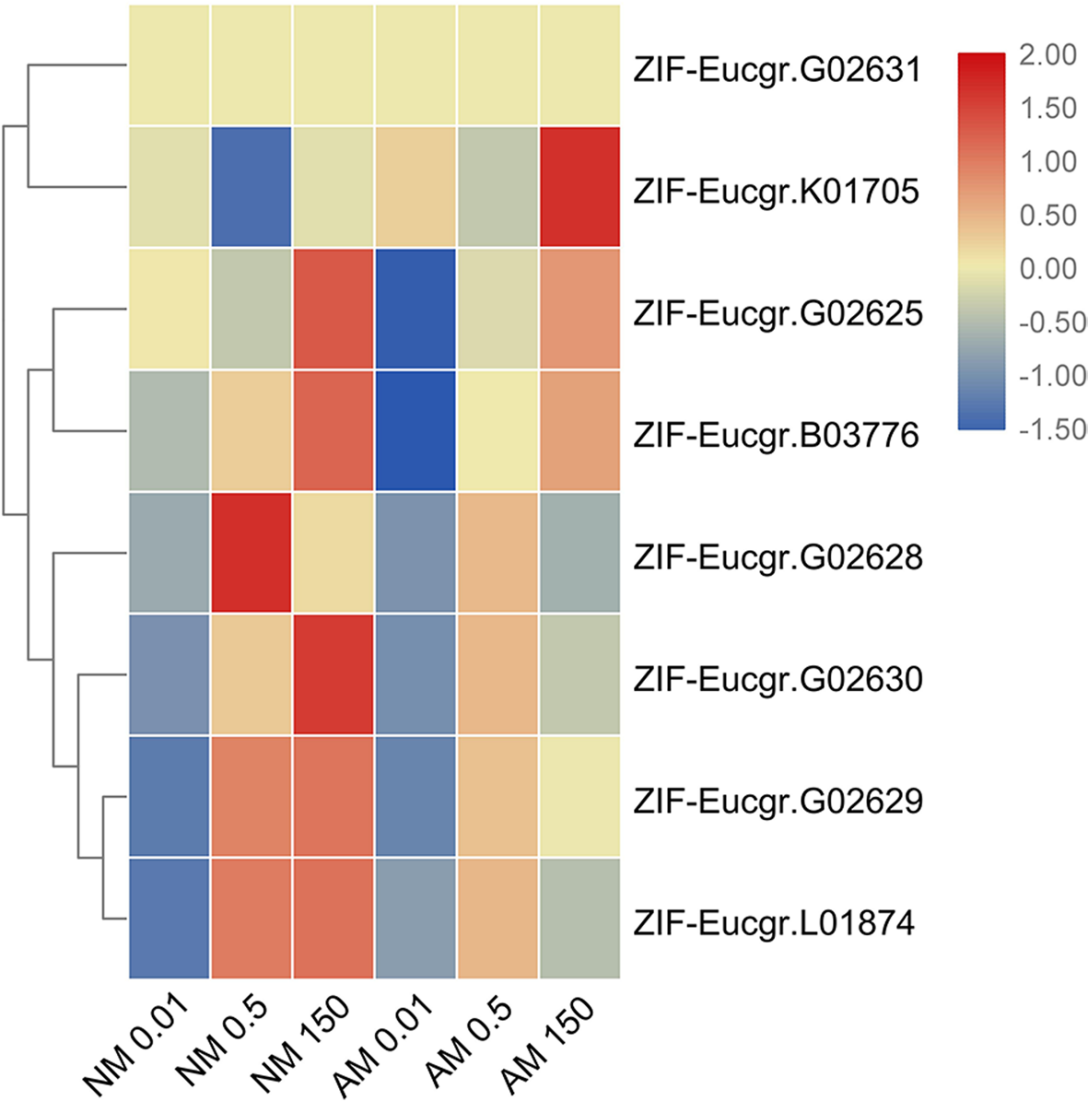
Heatmap of ZIFL family members. Cluster analysis of transcriptional fold-changes of ZIFL family gens in roots of non-mycorrhizal and mycorrhizal *E. grandis* exposed to 0.01, 0.5, or 150 μM ZnCl_2_. The color scale indicates fold-changes of mRNAs. For each gene, the expression levels in non-mycorrhizal roots exposed to 0.5 μM ZnCl_2_ were defined as 1, and the corresponding fold-changes under 0.01 and 150 μM ZnCl_2_ were calculated

We analyzed the expression of *EgZIFL* genes with different Zn treatments, between mycorrhizal and non-mycorrhizal plants, and in response to different Zn concentration stresses. The data on the expression of *EgZIFL* genes under different Zn concentrations for NM- and AMF-treated plants are shown in Fig. 4.

EgZIFL, AtZIF1, AtZIFL1, and PtZIFL2 were in a unified position on the evolutionary tree (Supplementary Fig. S14). The essential micronutrients Fe and Zn often limit plant growth but are toxic in excess. *PtZIFL2* was predicted to have a typical conserved domain (MFS structure and zinc finger structure), indicating that it is a hydrophobic TM secreted protein with a TM transport function when Zn is in excess. Through subcellular localization prediction analysis, these four proteins in *P. trichocarpa* were localized to the plasma membrane, while *AtZIF1* was localized to the vacuole membrane [63, 64]. The *AtZIF1* transporter is clearly involved in Zn homeostasis, as the loss-of-function *atzif1* mutant has altered Zn distribution and its transcription is upregulated by excess Zn [65]. We found that *ZIFL-Eucgr.G02630* was upregulated by a high Zn concentration (Fig. 4), which was consistent with the results of previous studies of this gene in *Arabidopsis*. This result indicates that *ZIFL-Eucgr.G02630* may be involved in Zn homeostasis and may thus be related to HM resistance in *Eucalyptus* spp*.* After inoculation with AMF at a high Zn concentration, the expression level of *ZIFL-Eucgr.G02630* decreased significantly, indicating that AMF is involved in maintaining a steady state of Zn to facilitate the resistance of *Eucalyptus* spp. to HMs*.*

We performed Pearson’s correlation analysis of the ZIFL family genes and found that the transcript levels of *ZIFL-Eucgr.G02629* and *ZIFL-Eucgr.L01874* (*R* = 0.829) were highly significantly correlated (*P* < 0.001, Supplementary Table. S5).

### Transporters that remove metals from the cytosol

#### HMA transport family

HMAs are proteins that hydrolyze ATP and use the released energy for TM transport. They also transport Zn^2 +^, Cd^2 +^, Pb^2 +^, Cu^2 +^, and other heavy metal ions across the membrane. HMA proteins transport heavy metal ions selectively. They may play an important role in the phytoremediation of contaminated soil. HMA family members are grouped into two distinct clades in phylogenetic analyses [66]. Members of one clade play roles in Cu and silver (Ag) transport, while members of the second clade function as Zn/cobalt (Co)/Cd/Pb transporters [67].

**Fig. 5:**
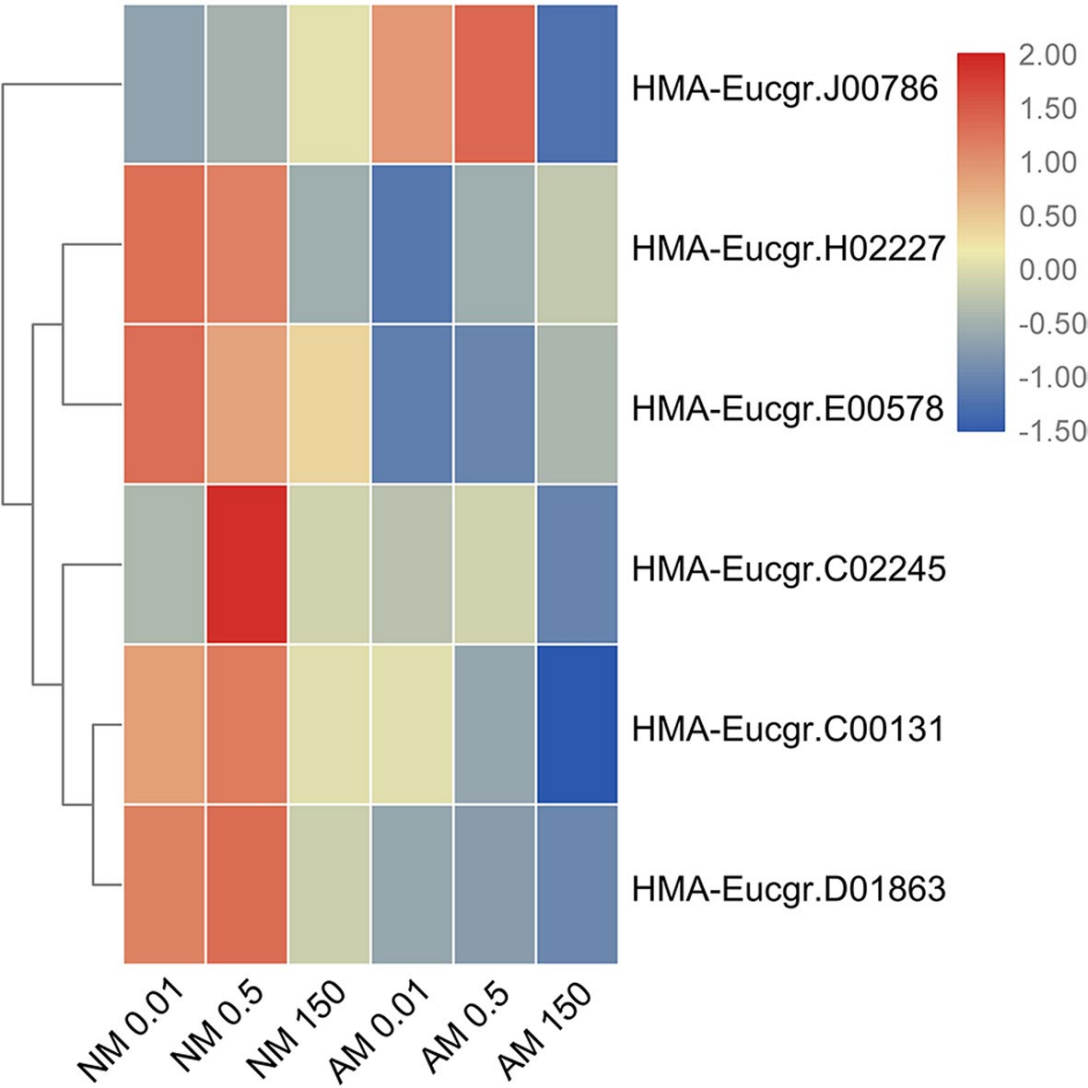
Heatmap of HMA family members. Cluster analysis of transcriptional fold-changes of HMA family gens in roots of non-mycorrhizal and mycorrhizal *E. grandis* exposed to 0.01, 0.5, or 150 μM ZnCl_2_. The color scale indicates fold-changes of mRNAs. For each gene, the expression levels in non-mycorrhizal roots exposed to 0.5 μM ZnCl_2_ were defined as 1, and the corresponding fold-changes under 0.01 and 150 μM ZnCl_2_ were calculated

In roots, the abundance of HMA family gene transcripts was higher after NM treatment than after AMF treatment under low-Zn conditions, except for *HMA-Eucgr.J00786* transcript levels, which were higher after AMF treatment. Every member of the HMA gene family had lower transcript levels after AMF treatment than after NM treatment under high-Zn conditions (Fig. 5).

To identify the evolutionary relationships between *HMA* genes of different plant species, a phylogenetic tree was constructed with 32 HMA transport protein sequences from 4 plant species (Supplementary Fig. S17). Bioinformatics analyses showed that HMA genes were well conserved and had similar physicochemical properties. Conserved motif structures were also detected (Supplementary Fig. S18). And the chromosomal location of HMA gene family were shown in the chromosome-scale genome of *E.grandis* (Supplementary Fig. S19).

HMA-Eucgr.C00131 and HMA-Eucgr.C02245 were found to be in the same branch of the phylogenetic tree as AtHMA2 and OsHMA2 (Supplementary Fig. S17). Therefore, these two genes are predicted to have the same function as members of the HMA2 subfamily. *OsHMA2* plays a role in Zn and Cd loading to the xylem and participates in root-to-shoot translocation of these metals in rice [68]. Eren et al. [69] found that *AtHMA2* is a high-affinity Zn transporter, transporting Zn out of cells to maintain a low Zn level in the cytoplasm and a steady-state balance of Zn within cells. The pericycle plasma membrane protein *AtHMA2* is responsible for loading Zn into the xylem, thus contributing to the control of the long-distance transport of Zn from roots to shoots [70]. The decrease in *HMA-Eucgr.C00131* and *HMA-Eucgr.C02245* gene expression levels under high-Zn conditions decreased the accumulation of Zn in the roots of *E. grandis*. After inoculation with AMF under high-Zn conditions, the expression of these genes was significantly inhibited, which may be because AMF symbiosis directs HMs to the roots and HM transporters at the plasmalemma or tonoplast of both symbiotic partners may catalyze the export of HMs from the cytoplasm [71].

HMA-Eucgr.J00227, AtHMA1, and OsHMA1 were found to be in the same branch of the phylogenetic tree (Supplementary Fig. S17). *OsHMA1* is highly upregulated by Zn deficiency in shoot tissue. *OsHMA1* may play a role in Zn efflux from plastids and may contribute to detoxification in the presence of excess Zn [72]. *AtHMA1* is located in the chloroplast envelope, where it contributes to Zn detoxification by reducing the Zn content in *A. thaliana* plastids under conditions of excess Zn [73]. Therefore, *HMA-Eucgr.J00227* is considered to have a Zn transport function, and its expression level decreased under high-Zn conditions. This may have been a result of Zn redistribution, indicating that this gene may have a detoxification function. After inoculation with AMF, HMs may bind to the cell wall and may be deposited in the vacuole of the fungus, which reduces the absorption of Zn by the plant. Thus, the expression level of this gene decreased after inoculation with AMF.

We found that *HMA-Eucgr.J00786* was induced by a high Zn concentration (Fig. 5), which was consistent with the expression pattern of *OsHMA9* (Supplementary Fig. S17). *OsHMA9* localizes to the plasma membrane and can discharge heavy metals to the outside of the cell, which may play a role in HM detoxification [74]. After inoculation with AMF, the expression level of *HMA-Eucgr.J00786* increased when the Zn concentration was low. The resulting promotion of Zn efflux from root cells then allows more Zn to be transported to the soil. The decrease in expression under high-Zn conditions may be due to the AM fungal absorption of Zn transported from plant cells.

We performed Pearson’s correlation analysis of HMA family genes and found that the transcript levels of *HMA-Eucgr.C00131* and *HMA-Eucgr.D01863* were highly significantly correlated (*R* = 0.773, *P* < 0.001, Supplementary Table. S6).

#### The cation efflux gene family

The CE gene family, also known as the cation diffusion facilitator gene family, was first described by Nies and Silver [75] and is considered to be compatible with Zn^2+^, Cd^2+^, Co^2+^, and Ni^2+^ transport. Members of this family are associated with the HM tolerance of plants. Most CE transporters are involved in storing metal ions within cells and transporting metal ions out of cells [76]. Many CE gene family members have been identified, and they have certain common structural features, including a C-terminal cation-binding domain, an N-terminal signal peptide sequence, and approximately six TM domains. The TM domains TM4-TM5 in CE family members in eukaryotes are all rich in histidine [77]. Most of the CE proteins are located in the cell membrane, but some are located in intracellular membrane systems, such as vacuole membranes and Golgi membranes.

To identify the evolutionary relationships between *CE* genes of different plant species, a phylogenetic tree was constructed with 44 CE transport protein sequences from 6 plant species (Supplementary Fig. S20). Bioinformatics analyses showed that CE genes were well conserved and had similar physicochemical properties. Conserved motif structures were also detected (Supplementary Fig. S21). And the chromosomal location of CE/MTP gene family were shown in the chromosome-scale genome of *E.grandis* (Supplementary Fig. S22).

**Fig. 6:**
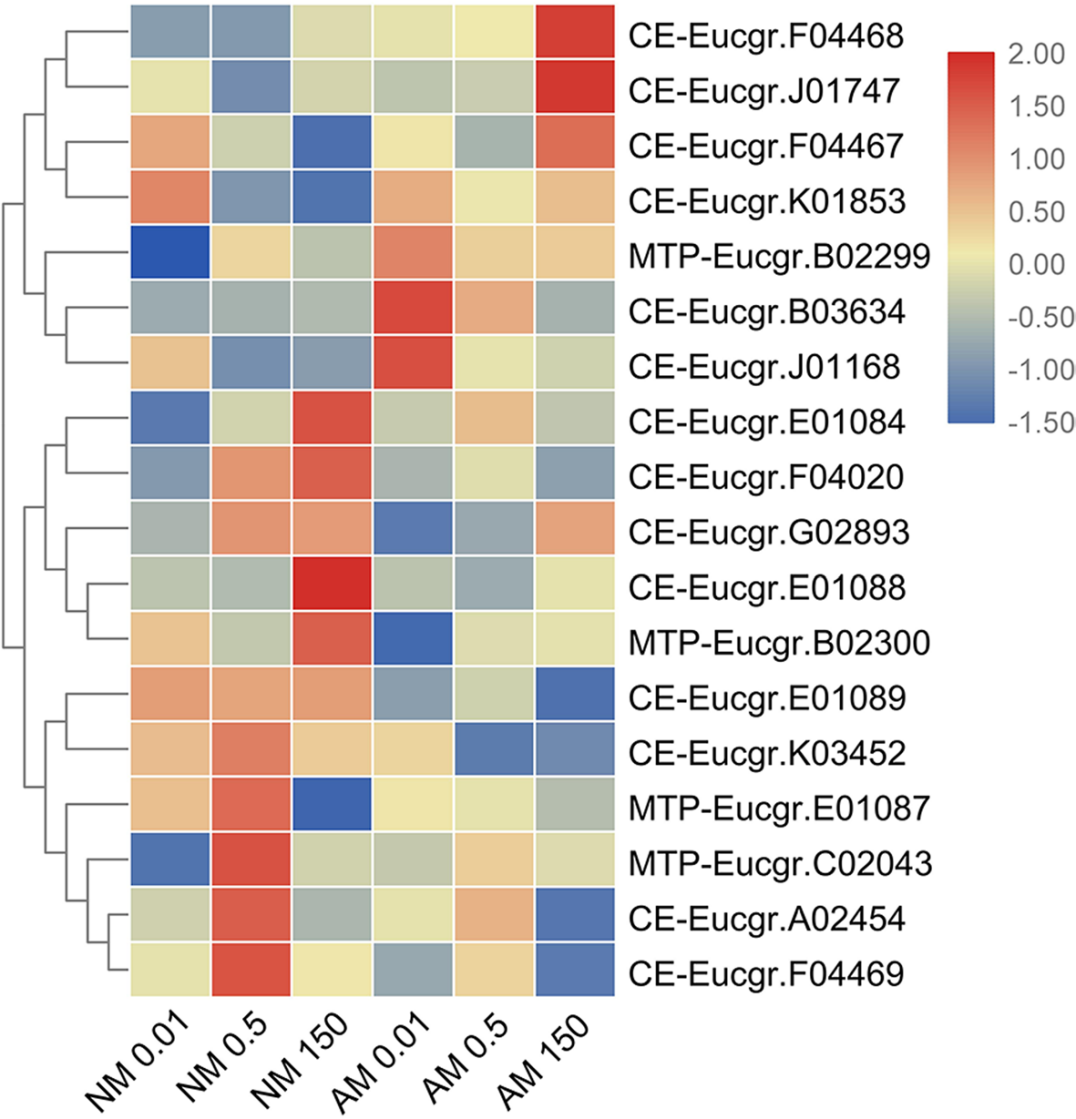
Heatmap of CE family members. Cluster analysis of transcriptional fold-changes of CE family gens in roots of non-mycorrhizal and mycorrhizal *E. grandis* exposed to 0.01, 0.5, or 150 μM ZnCl_2_. The color scale indicates fold-changes of mRNAs. For each gene, the expression levels in non-mycorrhizal roots exposed to 0.5 μM ZnCl_2_ were defined as 1, and the corresponding fold-changes under 0.01 and 150 μM ZnCl_2_ were calculated

Fourteen CE transporters were identified in the *E. grandis* genome based on sequencing results. Cluster analysis of transcript levels of the CE gene family clearly separated root samples from NM- and AMF-treated plants based on their responsiveness to Zn concentrations. According to variations in their transcript levels, they were divided into two subclusters. The two subclusters were distinguished by changes induced by AMF inoculation at a high Zn concentration (Fig. 6).

We found that CE-Eucgr.E01084, CE-Eucgr.E01089, and CE-Eucgr.E01088 were highly homologous and were in the same branch of the phylogenetic tree as OsMTP1, AtMTP1, AtMTP3, MtMTP1, and MtMTP3 (Supplementary Fig. S20). *AtMTP3* and *AtMTP1* are mainly expressed in the roots of *A. thaliana*. They transfer excess Zn from the cytoplasm to the vacuole and reduce the transport of Zn from the roots to the shoots [78]. We found that *CE-Eucgr.E01088* and *CE-Eucgr.E01084* transcript levels were upregulated after NM treatment under high-Zn conditions (Fig. 6), indicating that these two genes may have a similar function to *AtMTP3* in *Arabidopsis* (Supplementary Fig. S20), which transports excess Zn to the vacuoles in the roots. We conclude that *CE-Eucgr.E01088* and *CE-Eucgr.E01084* play crucial roles in avoiding Zn stress in the root meristematic tissue before the activation of Zn export systems and the synthesis of phytochelatins. After inoculation with AMF under high-Zn conditions, the transcriptional levels of *CE-Eucgr.E01084*, *CE-Eucgr.E01089*, and *CE-Eucgr.E01088* were suppressed. This may be because the mycelium increased Zn absorption under high-Zn conditions, resulting in the adsorption of more Zn to the cell wall of mycorrhizal fungi, thus reducing the absorption of Zn by plants [79].

We found that CE-Eucgr.J01168 was highly homologous to AtMTP11 (Supplementary Fig. S20). *Eucgr.J01168* had the highest transcriptional level at a low Zn concentration and the transcript levels of these genes were higher after AMF treatment than after NM treatment (Fig. 6). *AtMTP11* is involved in the tolerance of *A. thaliana* to high Mn concentrations. It is localized to the Golgi apparatus and is thought to remove Mn from the cell via the exocytosis of secretory vesicles [80]. *OsMTP11* is a trans-Golgi network-localized Mn transporter that is required for Mn homeostasis and contributes to the Mn tolerance of rice [81]. Therefore, we predicted that *Eucgr.J01168* may also be located in the Golgi apparatus. It was induced under low-Zn conditions and promoted the transport of Zn to the shoots, whereas under high-Zn conditions, it may be transported out of cells, thereby playing a role in cellular metal homeostasis.

We performed Pearson’s correlation analysis of CE/MTP family genes and found that the transcript levels of *CE-Eucgr.F04469* and *CE-Eucgr.A02454* (*R* = 0.849), *CE-Eucgr.J01747* and *CE-Eucgr.F04468* (*R* = 0.764), and *CE-Eucgr.K01853* and *CE-Eucgr.F04020* (*R* = 0.762) were highly significantly correlated (*P* < 0.001, Supplementary Table. S7).

### Improved metal tolerance through changes in nutrients

#### Phosphorus transporter genes

Phosphorus is an essential element for plants. It is involved in the synthesis of nucleic acids and ATP in cells, as well as the regulation of enzyme activity and signal transduction [82]. Pi is relatively inaccessible to plant roots because of its low solubility and high capacity for adsorption to soil particles. Plants must, therefore, use a complex series of Pi transporters to acquire Pi from the soil and distribute it to tissues and subcellular organelles. Pi acquisition at the root periphery in plants is coupled with proton entry (Pi:H^+^ symporter) and mediated by members of the *PHT1* gene family [83].

Pi-deficient plants over-accumulate Zn in the shoots and, conversely, Zn-deficient plants overaccumulate Pi in the shoots [28]. More than 90% of land plants form symbiotic associations with mycorrhizal fungi [83]. Mycorrhizae play an important role in the acquisition of P by the plant [29] and also facilitate Zn transport in the soil-fungi-plant continuum [19].

Thirty-five candidate PHT family genes were identified in the *E. grandis* genome. Bioinformatics analyses showed that PHT genes were well conserved and had similar physicochemical properties. Conserved motif structures were also detected (Supplementary Fig. S23). And the chromosomal location of PHT gene family were shown in the chromosome-scale genome of *E.grandis* (Supplementary Fig. S24). Through a heatmap analysis, we found that *PT-Eucgr.A02668*, *PT-Eucgr.K00323*, and *PT-Eucgr.J00101* were only expressed after inoculation with AMF. The expression levels of these genes were significantly reduced by a high Zn concentration. The expression level of *PT-Eucgr.A02668* was also reduced by a low Zn concentration. However, the expression levels of *PT-Eucgr.F03590, PT-Eucgr.I01609*, and *PT-Eucgr.H03069* increased significantly after inoculation with AMF under high-Zn conditions. Moreover, *PT-Eucgr.F03590* and *PT-Eucgr.H03069* expression levels were affected by NM treatment under low-Zn conditions (Supplementary Fig. S25).

PT-Eucgr.A02668, PT-Eucgr.K00323, and PT-Eucgr.J00101 showed homology with MtPT4 and OsPT11 (Supplementary Fig. S26). *MtPT4* of *Medicago truncatula* and *OsPT11* of *Oryza sativa* are localized in the perarbuscular membrane [84, 85]. Moreover, *MtPT4* RNAi lines and loss-of-function mutants fail to show symbiosis-associated increases in Pi or growth responses, indicating that *MtPT4* is required for Pi transport in the symbiotic system [86]. These data indicate that these three genes are phosphorus transporters specifically induced by mycorrhizal fungi and that they are affected by the Zn concentration.

PT-Eucgr.F03590, PT-Eucgr.I01609, and PT-Eucgr.H03069 showed homology with LePT2 and MtPT2 (Supplementary Fig. S26). Expression of these genes was induced under high-zinc conditions and increased after inoculation with AMF. The expression levels of *PT-Eucgr.F03590* and *PT-Eucgr.H03069* increased after NM treatment under low-Zn conditions. This result shows that after inoculation with AMF, the absorption of phosphorus is increased to reduce the toxic effect of heavy metals. PT-Eucgr.H03067 also showed homology with LePT2 and MtPT2. However, *PT-Eucgr.H03067* levels increased under high-Zn conditions after NM treatment and after inoculation with AMF; however, under these conditions, the expression levels were lower than those observed after NM treatment under low-Zn and high-Zn conditions. *LePT2* is predominantly expressed in Pi-deficient roots and is significantly downregulated in mycorrhizal roots under low-Pi conditions [87]. Therefore, in this sub-branch, the *Eucalyptus PHT* gene has evolved two functions to cope with the effects of changes in Zn concentration. The expression levels of MtPT2 within mycorrhizal roots are likely influenced by changes in the Zn status of the plant as a result of symbiotic function, as expression levels in the roots are inversely correlated with the P status of mycorrhizal plants [54].

We performed Pearson’s correlation analysis on PHT family genes and found that the transcript levels of *PHT-Eucgr.H03069* and *PHT-Eucgr.F03590* (*R* = 0.894) and *PHT-Eucgr.H03069* and *PHTEucgr.I01609* (*R* = 0.861) were highly significantly correlated (*P* < 0.001, Supplementary Table. S8).

#### AMTs and NRTs

Plants absorb and utilize two inorganic nitrogen sources, ammonium nitrogen (NH_4_^+^) and nitrate nitrogen (NO_3_^−^) [88]. The NRT family is responsible for transporting NO_3_^−^ and the AMT family is responsible for transporting NH_4_^+^ [89, 90].

Fourteen AMTs and eleven NRTs were identified in the *E. grandis* genome based on the sequencing results. Bioinformatics analyses showed that AMTs and NRTs were well conserved and had similar physicochemical properties. Conserved motif structures were also detected (Supplementary Fig. S27). And the chromosomal location of AMT and NRT gene family were shown in the chromosome-scale genome of *E.grandis* (Supplementary Fig. S28).

The expression levels of *NRT-Eucgr.H02533* decreased with increasing Zn concentration under NM treatment conditions, and its expression levels increased significantly after inoculation with AMF under high-Zn conditions. Based on the heat map, *AMT-Eucgr.K01403* and *AMT-Eucgr.K03320* are AMTs that are specifically induced by mycorrhizal fungi, as their expression levels decreased significantly after inoculation with AMF (Supplementary Fig. S29). Zn deficiency reduces the nitrogen content in the root system and increases the non-protein nitrogen content, which mainly affects RNA metabolism and consequently protein synthesis. The large accumulation of free amino acids, regardless of Zn content, causes a decrease in the levels of ammonium nitrogen and nitrate nitrogen [91]. Thus, N is critical for the uptake and accumulation of Zn in plants, and it deserves special attention in the biofortification of food crops with Zn [92].

We found that the expression levels of AMT family genes were decreased by a high Zn concentration and increased significantly after AMF inoculation. However, AMT gene expression levels induced by a low Zn concentration increased after inoculation with AMF, indicating that AMTs play roles in symbiosis with AMF at low Zn concentrations. Thus, nutrient absorption plays a role in improving the resistance of plants to high Zn concentrations.

We performed Pearson’s correlation analysis of genes in the AMT and NRT families and found that the transcript levels of *AMT-Eucgr.B02160* and *AMT-Eucgr.L03045, AMT-Eucgr.C01787* and *AMT-Eucgr.H05067*, and *AMT-Eucgr.I02296* and *AMT-Eucgr.K03320* were highly significantly correlated (*P* < 0.001, Supplementary Table. S9).

#### Potassium transporters

Potassium transporters are important for potassium uptake by plants. Based on their protein structure and function, they can be divided into the K^+^ uptake permease (KUP)/high-affinity K^+^ (HAK)/KT family, the HKT family, and the cation/proton antiporter (CPA) family, all of which are expressed in different plant tissues or organs. Cation and pH homoeostasis is regulated by monovalent CPAs that fall into two categories, the CPA1 family, which includes Na^+^/H^+^ NHX antiporters, and the CPA2 family, which includes cation/H^+^ (CHX) and K^+^ efflux antiporters (KEAs) [93].

Fifty-six candidate potassium transporter genes were identified in the *E. grandis* genome. Among them, the KUP/HAK/KT family was predicted to have 30 members (KTs: 4, HAKs: 23, CHXs: 23), and the CPA family was predicted to have 22 members (CHXs: 17, KEAs: 4, NHXs: 1). Bioinformatics analyses showed that subfamily of potassium transporters were well conserved and had similar physicochemical properties of each subfamily. Each subfamily conserved motif structures were also detected (Supplementary Fig. S30). And the chromosomal location of potassium transporters gene family were shown in the chromosome-scale genome of *E.grandis* (Supplementary Fig. S31). According to the heat map, 9 genes were induced by Zn and mycorrhizae (KTs: 1, KUPs: 2; HAKs: 4; CHXs: 2) (Supplementary Fig. S32).

A high Zn concentration reduced the levels of *HAK-Eucgr.C02191*, *HAK-Eucgr.C00265,* and *HAK-Eucgr.G02011* transcripts in NM-treated roots. AMF colonization increased the levels of *HAK-Eucgr.C02191* transcripts and reduced the levels of *HAK-Eucgr.C00265* and *HAK-Eucgr.G02011* transcripts. However, a high Zn concentration increased the levels of *HAK-Eucgr.F02234, NHX-Eucgr.F00635,* and *HAK-Eucgr.G01991* transcripts. Moreover, AMF colonization increased the levels of *HAK-Eucgr.F02234* transcripts and reduced the levels of *HAK-Eucgr.G01991* and *NHX-Eucgr.F00635* transcripts (Supplementary Fig. S32).

Many previous studies have found that certain physiological processes that are important for plant growth, such as cell stretching and shock motion, are also related to the regulation of potassium ions in plants to maintain cell turgor and osmotic potential [94]. In summary, the effect of potassium ions on the regulation of plant cell turgor and osmotic potential has important physiological significance in the normal growth and development of plants.

We performed Pearson’s correlation analysis of potassium transporter genes and found that the transcript levels of *KT-Eucgr.C04163* and *KUP-Eucgr.B03355* (*R* = 0.821), *KUP-Eucgr.B03355* and *KT-Eucgr.C04163* (*R* = 0.821), *KUP-Eucgr.B03355* and *HAK-Eucgr.C02265* (*R* = 0.744), *KUP-Eucgr.B03355* and *HAK-Eucgr.G02011* (*R* = 0.889), *KUP-Eucgr.B03355* and *CHX-Eucgr.K03153* (*R* = 0.750), *HAK-Eucgr.C02265* and *HAK-Eucgr.G02011* (*R* = 0.747), *HAK-Eucgr.C02265* and *CHXEucgr.E00818* (*R* = 0.861), *HAK-Eucgr.C02265* and *CHXEucgr.K03153* (*R* = 0.939), *CHXEucgr.E00818* and *HAK-Eucgr.G02011* (*R* = 0.733), and *CHXEucgr.E00818* and *CHXEucgr.K03153* (*R* = 0.771) were highly significantly correlated (*P* < 0.001, Supplementary Table. S10).

#### PCA of heavy metal transporter and nutrient transporter responses

**Fig. 7:**
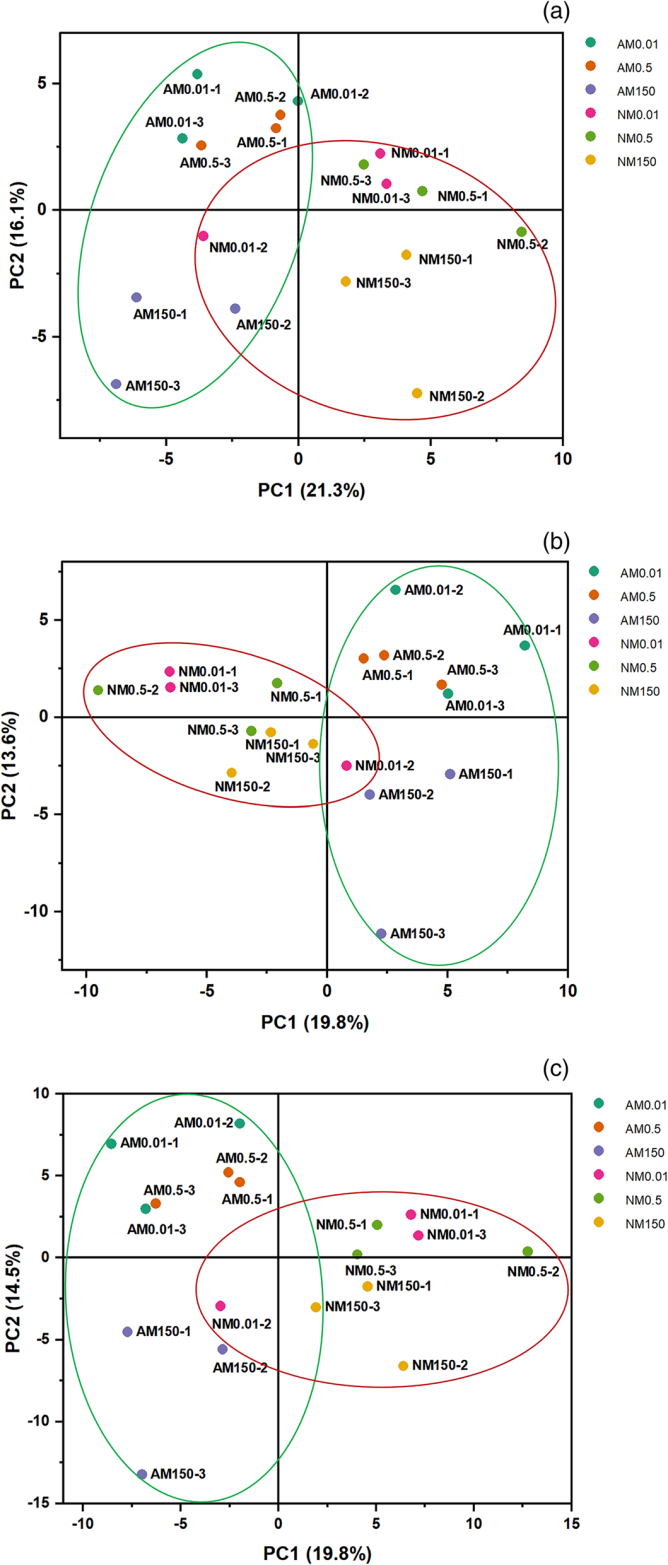
Principal component analysis (PCA) plot of the first two principal components in roots of non-mycorrhizal and mycorrhizal *E. grandis.* The analysis was conducted using data of heavy metal-related and nutrition-related transporters of non-mycorrhizal and mycorrhizal *E. grandis* exposed to 0.01, 0.5150 μM ZnCl_2_. (**a**) Heavy metal related transporters (**b**) Nutrition-related transporters (**c**) Heavy metal-related and nutrition-related transporters

A PCA was performed using the data of related genes involved in HM and nutrient responses. In the PCA plot, a greater distance between symbols associated with Zn concentration suggested a stronger responsiveness of HM and nutrient transporters to changes in Zn concentration. The results of the PCA indicated that nutrient-related transporters were clustered together, but separately in AMF- and NM-treatment groups, at different Zn concentrations, indicating that AMF symbiosis may improves the metal stress resistance of plants mainly through nutrient regulation (Fig. 7).

## Conclusions

In the presence of symbiosis with AMF, we explored the variation in the transcription levels of six HM-related gene families under high-Zn conditions. The results indicated that the expression levels of one *ZNT* gene, three *YSL* genes, and one *COPT* gene were significantly upregulated, while those of one *ZIFL* gene and one Ctr gene were significantly downregulated(*p* < 0.05).

**Fig. 8:**
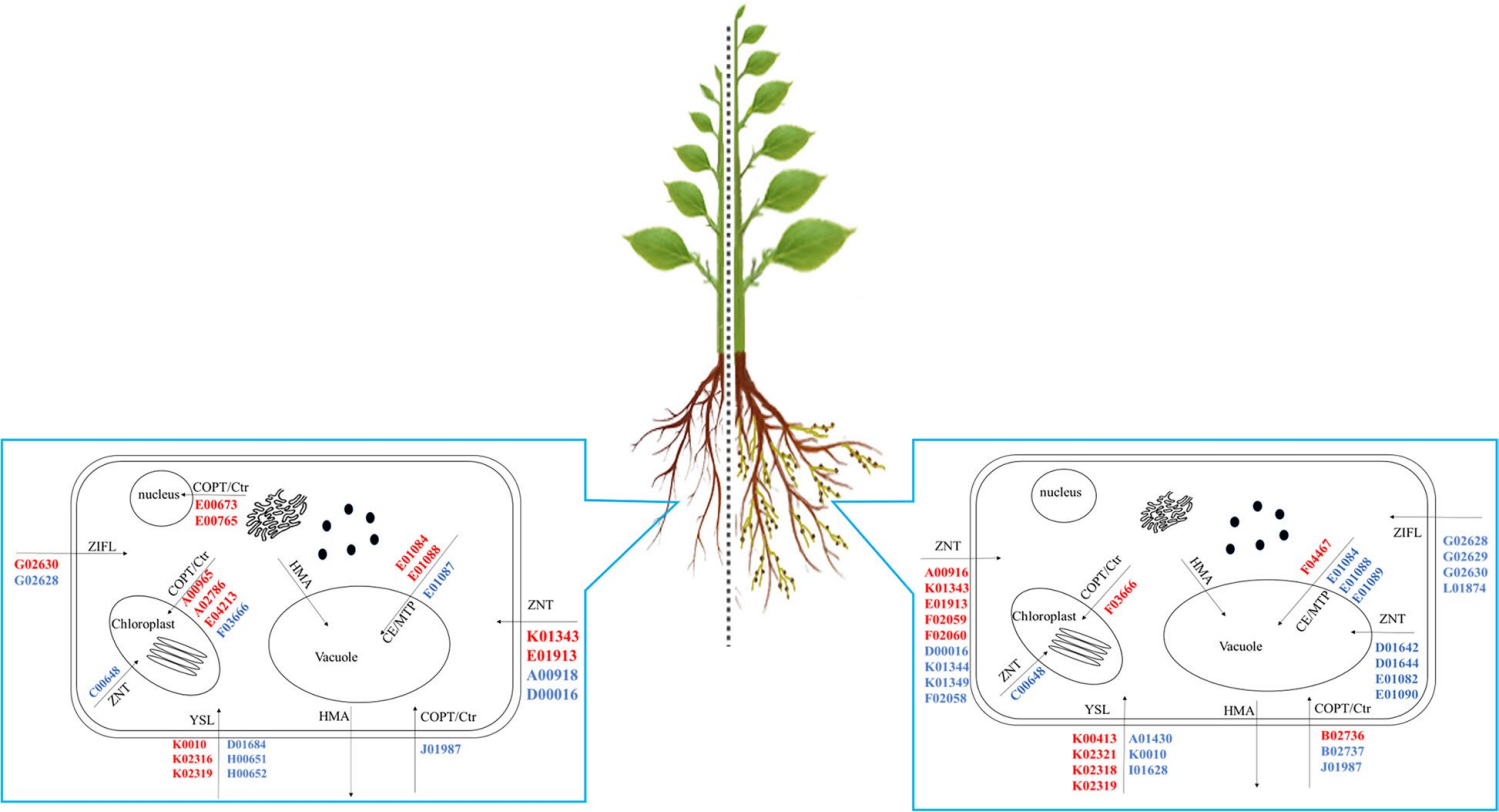
Graphical representation of plant responses to zinc (Zn) excess and resupply of Zn in uptake and translocation of Zn and the expression of the Heavy metal transporter in *E. grandis.* Blue arrows, increased levels; red arrows, reduced levels

Under high-Zn conditions, genes related to the absorption of nutrients, mainly N, P, and K, were analyzed. We found that the expression levels of two *NRT2* transporter genes, and one *HAK* transporter gene were significantly downregulated(*p* < 0.05). There was no significant upregulation of nutrient-related genes, indicating that a high Zn concentration inhibits the growth and development of plants. Meanwhile, under AMF symbiosis and high-Zn conditions, the expression levels of seven *PHT* genes, one *NRT1* gene, two *NRT2* genes, one *HAK* gene, and three *AMT* genes were significantly upregulated (*p* < 0.05), whereas those of only one *PHT* gene and one *NRT1* gene were significantly downregulated (*p* < 0.05) (Fig. 8).

Here, we showed that AMF increases the resistance of *E*. *grandis* to high-Zn stress by improving the absorption of nutrients by the plant and regulating Zn uptake at the gene transcription level. Among theses genes, 41 heavy metal transporters (ZNT:15, YSL:11, COPT/Ctr:8, CE/MTP:3, ZIFL:4) and 36 nutrition-related transporters are involved in Zn tolerance in *E. grandis* with AMF symbiosis. Although these genes’ function was inferred from their homologous genes’ function in model herbaceous plants, these candidate genes’ multiple function still need to be investigated at whole plant and tissue level for the woody perennial plants having unique physiological and anatomical structure. Futhermore, the interaction between HM tolerance and nutrient acquisition should be furtherly explored to facilitate the genetic improvement of nutrient utilization in *E*. *grandis* at the HM-contaminated areas.

## Materials and methods

### Plant cultivation, inoculation, and Zn treatment

The seedlings needed for the experiment were taken from *E. grandis* clone GL1, kindly provided by Dr. Chunjie Fan from the Research Institute of Tropical Forestry, Chinese Academy of Forestry, Guangzhou, China. The AMF species *Rhizophagus irregularis* (BGCBJ09) was purchased from the Beijing Academy of Agriculture and Forestry Sciences (Beijing, China). The AMF was propagated using *Trifolium repens*, and the inoculum was collected to obtain spores. The AMF spores were obtained by sucrose-gradient centrifugation [95]. Uniform *E. grandis* seedlings were selected and transplanted into plastic pots (9 cm high, 10 cm diameter) with sterile medium (quartz sand:vermiculite, V:V = 2:1). Seedlings receiving inoculation treatment (hereafter referred to as the AMF-treatment group) were inoculated with 600 spores, which were added to the medium in each pot. Seedlings in the non-inoculation group were not inoculated with AMF spores (referred to as the NM-treatment group).

Plants were grown in a growth chamber with 25/20 °C day/night temperatures, 60% relative humidity, and a 16/8-h light/dark photoperiod. To generate highly colonized roots, for 45 days post-inoculation (dpi), the plants were supplied with 50 mL of Long-Ashton solution [96] with a modified monosodium phosphate (NaH_2_PO_4_) concentration (30 μM) every 3 days. This solution contained 0.5 μM Zn^2+^. At 46 day, each pot was immersed in a beaker filled with distilled water. The water was changed five times, once every 2 h, to remove residual nutrients from the substrates. The AMF- and NM-treatment groups were then divided into three sub-groups: low Zn concentration (0.01 μM Zn supplied as ZnCl_2_), normal Zn concentration (0.5 μM Zn supplied as ZnCl_2_), and high Zn concentration (150 μM Zn supplied as ZnCl_2_). Zinc treatment were supplied with 50 mL of Long-Ashton solution with different Zn concentration every three days for fifteen days, five times in total. There were six pots (replicates) for each Zn treatment.

### Arbuscular mycorrhizal colonization rate measurement

Mycorrhizal-colonized roots were stained with Wheat germ agglutinin-conjugated Alexa Fluor®488 (WGA-AF488) as described previously [97]. Briefly, harvested roots were placed in 50% ethanol for more than 4 h and then transferred to 20% (w/v) KOH for 2–3 days, followed by 0.1 M HCl for 2 h at room temperature. After removing the HCl, the samples were rinsed twice with distilled water and once with phosphate-buffered saline (PBS, pH 7.4), and then immersed in PBS/WGA-Alexa Fluor 488 staining solution (0.2 μg/mL) in the dark for more than 6 h at 37 °C. The arbuscular mycorrhizal colonization rate was determined under a fluorescence microscope (NIKON, Eclipse Ni-U) using the gridline intersect method described by Giovannetti and Mosses (2010).

### Acid digestion and determination of Zn and inorganic phosphate (Pi) content

Dried leaves and roots were ground and digested (50 mg) for 8 h in 5 mL of 6 M nitric acid (HNO_3_) in closed-glass tubes on heating blocks at 90 °C [98]. Extracts were diluted in a solution of 2% HNO_3_. Pi and Zn concentrations were determined by inductively coupled plasma mass spectrometry (Thermo Scientific™ iCAP™ Q ICP-MS; Thermo Fisher Scientific, Waltham, MA, USA).

### cDNA library construction and sequencing

Total RNA was extracted from individual samples using the RNAprep Pure Plant Kit (Tiangen, Beijing, China). For roots from each subgroup, three independent biological replicates were analyzed. RNA quality and concentration were determined by 1% agarose gel electrophoresis and spectrophotometry, respectively. mRNA was purified from total RNA using the TruSeq RNA Sample Prep Kit (Illumina, San Diego, CA, USA) with oligo-dT magnetic beads. mRNA samples were fragmented into 150-bp fragments using a chemical reagent at high temperature. Double-stranded cDNA was synthesized using random hexamer primers and end-repaired with an exonuclease and a polymerase. The cDNA fragments and sequencing adapters were ligated with T4 DNA ligase (Thermo Scientific), according to the manufacturer’s protocol, and sequenced using an Illumina HiSeq™ 2000 sequencing system.

### Transcriptome assembly

Raw data (raw reads) were first processed using in-house Perl scripts. Clean data (clean reads) were obtained by removing reads containing adapter sequences, poly-Ns, and low-quality reads from the raw data. Meanwhile, the number of bases scoring Q20 and Q30, the GC content, and the sequence duplication level of the clean data were calculated.. All downstream analyses were based on clean data of high quality. Transcriptome assembly was accomplished using Trinity with min_kmer_cov set to 2 and all other parameters set to default values [99].

### Gene annotation

Gene functional annotation was performed by sequence comparison with public databases. The Basic Local Alignment Search Tool was used to search for homology (E value <0.00001) between unique sequences and JGI nonredundant proteins (https://phytozome.jgi.doe.gov/pz/portal.html#!info?alias=Org_Egrandis), and Swiss-Prot (http://www.expasy.ch/sprot) databases were searched for protein sequence analysis. The best-hit transcripts were selected as unigenes. The Blast2GO program was then used to obtain Gene Ontology (GO) annotations and functional classification of all unigenes [100]. Enzyme Commission terms and biochemical pathway information were generated using Kyoto Encyclopedia of Genes and Genomes (KEGG) (http://www.genome.jp/kegg/). Evolutionary genealogy of genes: Non-supervised Orthologous Groups (eggNOG) (http://eggnog.embl.de/) was used to predict and classify potential functions based on known orthologous gene products [101].

### Gene expression, signaling pathway analysis, and differential gene identification

Based on the number of reads mapping to a particular gene, the reads per kilobase per million reads (RPKM) metric was used to estimate the transcript levels of the genes [102]. Briefly, the RPKM value was calculated by dividing the number of reads mapped to each gene by the length of the gene and the number of reads from the library to compensate for slightly different read depths for different samples. An RPKM threshold value of 0.1 was set to detect the presence of a unigene, which corresponds to a false discovery rate (FDR) of 5% [103].

The DESeq program (http://www-huber.embl.de/users/anders/DESeq/) was used for the statistical analysis of differentially expressed genes (DEGs) between two samples [104]. DEGs were identified according to a difference in expression > two-fold and a significant *p-*value (padj <0.05), after adjusting for the FDR due to multiple testing procedures to minimize the chance of a type I error [105]. GO and KEGG analyses were also used to evaluate DEGs in a variety of biological pathways. Based on information from Nr (Non-Redundant Protein Sequence Database), eggNOG, GO, and KEGG analyses, DEGs involved in metamorphosis, immunity, and sensory perception were further investigated manually. The KEGG metabolic pathways for all unigenes with GO terms were constructed using an online tool with different colors to indicate different gene expression levels (http://www.genome.jp/kegg/tool/map_pathway2.html). Functional domain analysis was performed using ExPASy PROSITE (http://www.expasy.ch/tools/scanprosite/).

### Data analyses

Gene families related to the transport and distribution of metals and nutrients in plants were selected for further analysis. These included zinc transporter (ZNT), copper transporter (COPT), zinc-induced facilitator (ZIF), yellow stripe-like (YSL), heavy metal ATPase (HMA), cation efflux (CE), phosphate transporter (PHT), ammonium transporter (AMT), nitrate transporter (NRT), and potassium transporter gene families. A cluster analysis of the transcriptional levels of these gene families was performed using TBtools (gitub.com/CJ-chen/TBtools). Phylogenetic analyses of each gene family were performed using the neighbor-joining method implemented in MEGA 6 (www.megasoftware.net). For principal component analysis (PCA), data were standardized and computed using Origin2021b (https://www.originlab.com/). Pearson’s correlation analyses were performed using SPSS (IBM, Armonk, NY, USA).

## Supplementary Information

**Additional file 1: Table S1.** General information of ZNT transporter genes identified in *Eucalyptus grandis.* **Table S2.** Pearson’s correlation analysis on ZNT family genes. **Table S3.** Pearson’s correlation analysis on COPT/Ctr family genes. **Table S4.** Pearson’s correlation analysis on YSL family genes. **Table S5.** Pearson’s correlation analysis on ZIFL family genes. **Table S6.** Pearson’s correlation analysis on HMA family genes. **Table S7.** Pearson’s correlation analysis on CE/MTP family genes. **Table S8.** Pearson’s correlation analysis on PHT family genes. **Table S9.** Pearson’s correlation analysis on AMT and NRT family genes. **Table S10.** Pearson’s correlation analysis on Potassium transporters family genes.

**Additional file 2.**

**Additional file 3.**

**Additional file 4.**

**Additional file 5.**

## Data Availability

The datasets supporting the conclusion of this article are included within the article and its additional files, all gene expression data were deposited in Box (https://app.box.com/s/2a1z0u7pekdpk8cnoa43h6fcpmh2f45b) and Galaxy Project (https://usegalaxy.org/datasets/bbd44e69cb8906b568ca01481728cd72/display?to_ext=tabular) and Eucalyptus grandis reference genome from Phytozome database (https://phytozome.jgi.doe.gov/pz/portal.html#!info?alias=Org_Egrandis), as well as can be requested from HWT (hwt@scau.edu.cn).
