# Supplementary material for: Transcriptional regulation of metal metabolism- and nutrient absorption-related genes in Eucalyptus grandis by arbuscular mycorrhizal fungi at different zinc concentrations
Source: BMC Plant Biol. 2022 Feb 22;22:76. doi: 10.1186/s12870-022-03456-5 (PMC8862258; doi:10.1186/s12870-022-03456-5)

Supplementary table Table 1. General information of ZNT transporter genes identified in Eucalyptus grandis

| **Gene ID** | **ORF Length** | **Location Coordinates** | | | **Chr** | **Protein** | | |
| --- | --- | --- | --- | --- | --- | --- | --- | --- |
|  |  | **Start Site** | **End Site** | **Direction** |  | **Length(a.a.)** | **PI** | **Mol.Wt.(kDa)** |
| Eucgr.A00916 | 1852 | 11034269 | 11037194 | - | Chr01 | 364 | 6.91 | 38342.73 |
| Eucgr.A00918 | 1071 | 11019062 | 11021449 | - | Chr01 | 356 | 6.99 | 37485.4 |
| Eucgr.A00921 | 900 | 10944869 | 10946878 | - | Chr01 | 299 | 6.82 | 31373.76 |
| Eucgr.D00016 | 2279 | 123926 | 127148 | - | Chr04 | 234 | 6.48 | 24666.64 |
| Eucgr.K01343 | 1703 | 16785145 | 16787333 | + | Chr11 | 406 | 8.76 | 43013 |
| Eucgr.K01344 | 1392 | 16791295 | 16793286 | + | Chr11 | 374 | 7.25 | 39677.69 |
| Eucgr.K01345 | 882 | 16799599 | 16802819 | + | Chr11 | 293 | 5.97 | 30904.11 |
| Eucgr.K01348 | 1115 | 16815316 | 16816935 | + | Chr11 | 346 | 6.04 | 36692.76 |
| Eucgr.E01913 | 891 | 24886255 | 24888744 | + | Chr05 | 297 | 7.23 | 31716.64 |
| Eucgr.K01349 | 861 | 16818839 | 16820705 | + | Chr11 | 287 | 6.24 | 30604.62 |
| Eucgr.F02058 | 1434 | 27146566 | 27148813 | + | Chr06 | 336 | 5.75 | 35542.38 |
| Eucgr.F02059 | 1005 | 27161213 | 27163017 | + | Chr06 | 334 | 8.45 | 35803.59 |
| Eucgr.F02060 | 1050 | 27168937 | 27170681 | + | Chr06 | 349 | 5.96 | 37524.52 |
| Eucgr.C00648 | 1730 | 11872539 | 11875899 | - | Chr03 | 422 | 6.03 | 45108.86 |
| Eucgr.E01901 | 1249 | 24545012 | 24546371 | + | Chr05 | 381 | 7.04 | 39667.47 |
| Eucgr.D01642 | 2399 | 29898164 | 29907652 | - | Chr04 | 440 | 5.99 | 48588.18 |
| Eucgr.D01644 | 1441 | 29921818 | 29925333 | - | Chr04 | 392 | 5.89 | 44529.33 |
| Eucgr.E01082 | 1692 | 10473702 | 10477543 | + | Chr05 | 415 | 5.77 | 45975.51 |
| Eucgr.E01090 | 1534 | 10564662 | 10566195 | + | Chr05 | 421 | 5.86 | 46522.2 |

**1 Illumina sequencing and assembly**

The transcriptome profile of root samples from mycorrhizal and non-mycorrhizal *E*. *grandis* samples were determined under conditions of 0.01 μM, 0.5 μM, and 150 μM ZnCl_2_. For each treatment, three replicates were sequenced using an Illumina NextSeq500 instrument. Approximately 50 million reads of raw tags were obtained for each sample (Table A). Data cleaning provided 793,504,720 (110.9 GB) clean reads. All error rates were less than 0.1%. The general base quality value reached 30%. The GC content ranged from 40% to 50% (Fig. A). These results indicated high-quality sequencing data and confirmed the feasibility of using the data in subsequent analyses. The Pearson’s correlation coefficients of the three replicates of each treatment were greater than 80%, suggesting appreciable correlation (Table B). Ninety percent of the reads were mapped to the *E. grandis* genome, and most of the reads were uniquely mapped (Table C). Approximately 90% of the mapped reads were mapped to gene regions, and more than 98% were mapped to exons (Table C). Further, 36,350 sequenced genes were recovered and were distributed across 11 chromosomes (Fig. B).

TableA: Calculate the Raw Data of each sample separately

| **Sample** | **Reads NO.** | **Bases(bp)** | **Q30(bp)** | **N%** | **Q20(%)** | **Q30(%)** |
| --- | --- | --- | --- | --- | --- | --- |
| NM 0.01-1 | 53066402 | 7959960300 | 7343221801 | 0.000116 | 96.97 | 92.25 |
| NM 0.01-2 | 47288376 | 7093256400 | 6552107411 | 0.000114 | 97.02 | 92.37 |
| NM 0.01-3 | 53246386 | 7986957900 | 7397963872 | 0.000114 | 97.14 | 92.62 |
| NM 0.5-1 | 47181390 | 7077208500 | 6589091491 | 0.000118 | 97.34 | 93.1 |
| NM 0.5-2 | 49637312 | 7445596800 | 6896653901 | 0.000115 | 97.14 | 92.62 |
| NM 0.5-3 | 43571640 | 6535746000 | 5998060386 | 0.000117 | 96.75 | 91.77 |
| NM 150-1 | 49927798 | 7489169700 | 6939683062 | 0.000115 | 97.16 | 92.66 |
| NM 150-2 | 47173916 | 7076087400 | 6551062198 | 0.00012 | 97.11 | 92.58 |
| NM 150-3 | 50182360 | 7527354000 | 7008386910 | 0.000115 | 97.35 | 93.1 |
| AM 0.01-1 | 43994118 | 6599117700 | 6123236848 | 0.000118 | 97.21 | 92.78 |
| AM 0.01-2 | 46515882 | 6977382300 | 6453760968 | 0.000116 | 97.09 | 92.49 |
| AM 0.01-3 | 45615882 | 6848846100 | 6337340968 | 0.000114 | 97.1 | 92.53 |
| AM 0.5-1 | 45658974 | 6904548900 | 6357392162 | 0.000115 | 96.88 | 92.07 |
| AM 0.5-2 | 46030326 | 6522595500 | 6029309336 | 0.000112 | 97.04 | 92.43 |
| AM 0.5-3 | 43483970 | 6612018600 | 6121863105 | 0.000114 | 97.1 | 92.58 |
| AM 150-1 | 44080124 | 7245426300 | 6702221732 | 0.000111 | 97.08 | 92.5 |
| AM 150-2 | 49124902 | 7368735300 | 6806647035 | 0.000115 | 97.02 | 92.37 |
| AM 150-3 | 45978936 | 6896840400 | 6364358271 | 0.000111 | 96.95 | 92.27 |

Table B: Summary of read statistics from RNA-sequencing of *Eucalyptus grandi*

| **Sample** | **Clean Reads No.** | **Clean Data (bp)** | **Clean Reads %** | **Clean Data %** | **Total_Mapped** | **Map_Events** | **Mapped_to_gene** |
| --- | --- | --- | --- | --- | --- | --- | --- |
| NM 0.01-1 | 49180430 | 7377064500 | 92.67 | 92.67 | 44337228（90.15%） | 42460364 | 38859644（91.52%） |
| NM 0.01-2 | 43868146 | 6580221900 | 92.76 | 92.76 | 39340182（89.68%） | 37964751 | 34827829（91.74%） |
| NM 0.01-3 | 49307496 | 7396124400 | 92.6 | 92.6 | 43732268（88.69%） | 42266496 | 38880160（91.99%） |
| NM 0.5-1 | 43712496 | 6556874400 | 92.64 | 92.64 | 39623275（90.65%） | 38308506 | 35249785（92.02%） |
| NM 0.5-2 | 46058130 | 6908719500 | 92.78 | 92.78 | 41067230（89.16%） | 39617839 | 36107748（91.14%） |
| NM 0.5-3 | 40503920 | 6075588000 | 92.95 | 92.95 | 36329133（89.69%） | 35067883 | 32296352（92.10%） |
| NM 150-1 | 46375906 | 6956385900 | 92.88 | 92.88 | 42102978（90.79%） | 40676858 | 37399236（91.94%） |
| NM 150-2 | 43703186 | 6555477900 | 92.64 | 92.64 | 38885410（88.98%） | 37521032 | 34385006（91.64%） |
| NM 150-3 | 46515620 | 6977343000 | 92.69 | 92.69 | 42146431（90.61%） | 40691217 | 37274261（91.60%） |
| AM 0.01-1 | 40693338 | 6104000700 | 92.49 | 92.49 | 35648299（87.60%） | 34448833 | 31745651（92.15%） |
| AM 0.01-2 | 43298580 | 6494787000 | 93.08 | 93.08 | 36348006（83.95%） | 35153440 | 32243204（91.72%） |
| AM 0.01-3 | 42406560 | 6360984000 | 92.87 | 92.87 | 37269247（87.89%） | 35914411 | 32898969（91.60%） |
| AM 0.5-1 | 42771402 | 6415710300 | 92.92 | 92.92 | 37443498（87.54%） | 36068852 | 33007586（91.51%） |
| AM 0.5-2 | 40469892 | 6070483800 | 93.06 | 93.06 | 35679313（88.16%） | 34456821 | 31591975（91.69%） |
| AM 0.5-3 | 41042058 | 6156308700 | 93.1 | 93.1 | 35852043（87.35%） | 34548246 | 31833202（92.14%） |
| AM 150-1 | 45152538 | 6772880700 | 93.47 | 93.47 | 39692045（87.91%） | 38271465 | 35247476（92.10%） |
| AM 150-2 | 45645116 | 6846767400 | 92.91 | 92.91 | 40365561（88.43%） | 38891214 | 35729324（91.87%） |
| AM 150-3 | 42799906 | 6419985900 | 93.08 | 93.08 | 37576878（87.80%） | 36170792 | 33144180（91.63%） |

Fig. A: The GC content (Base content distribution)


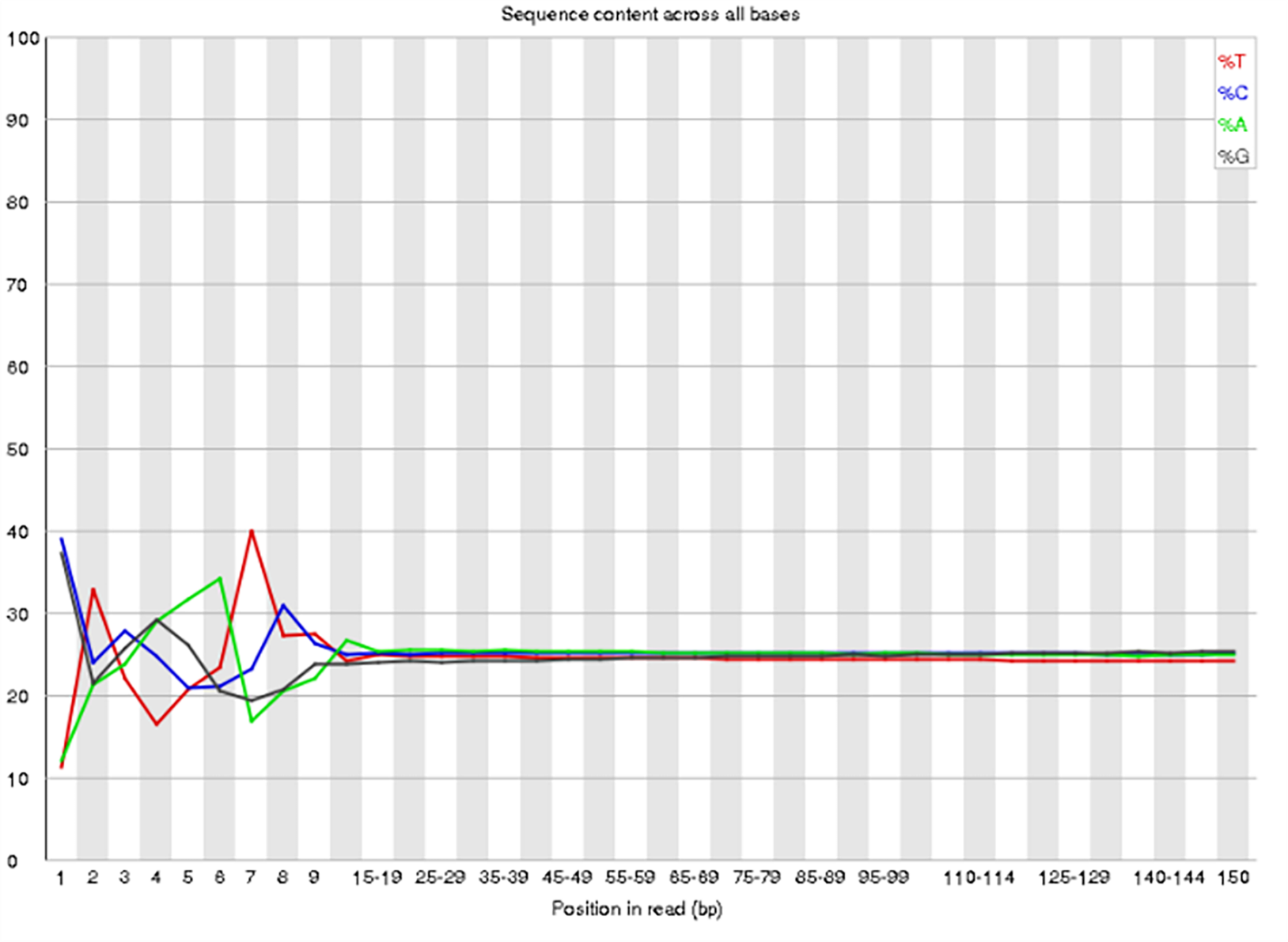


Note: The abscissa is the position of the base in Reads (5’->3’), and the ordinate is the proportion of a certain base at that position.


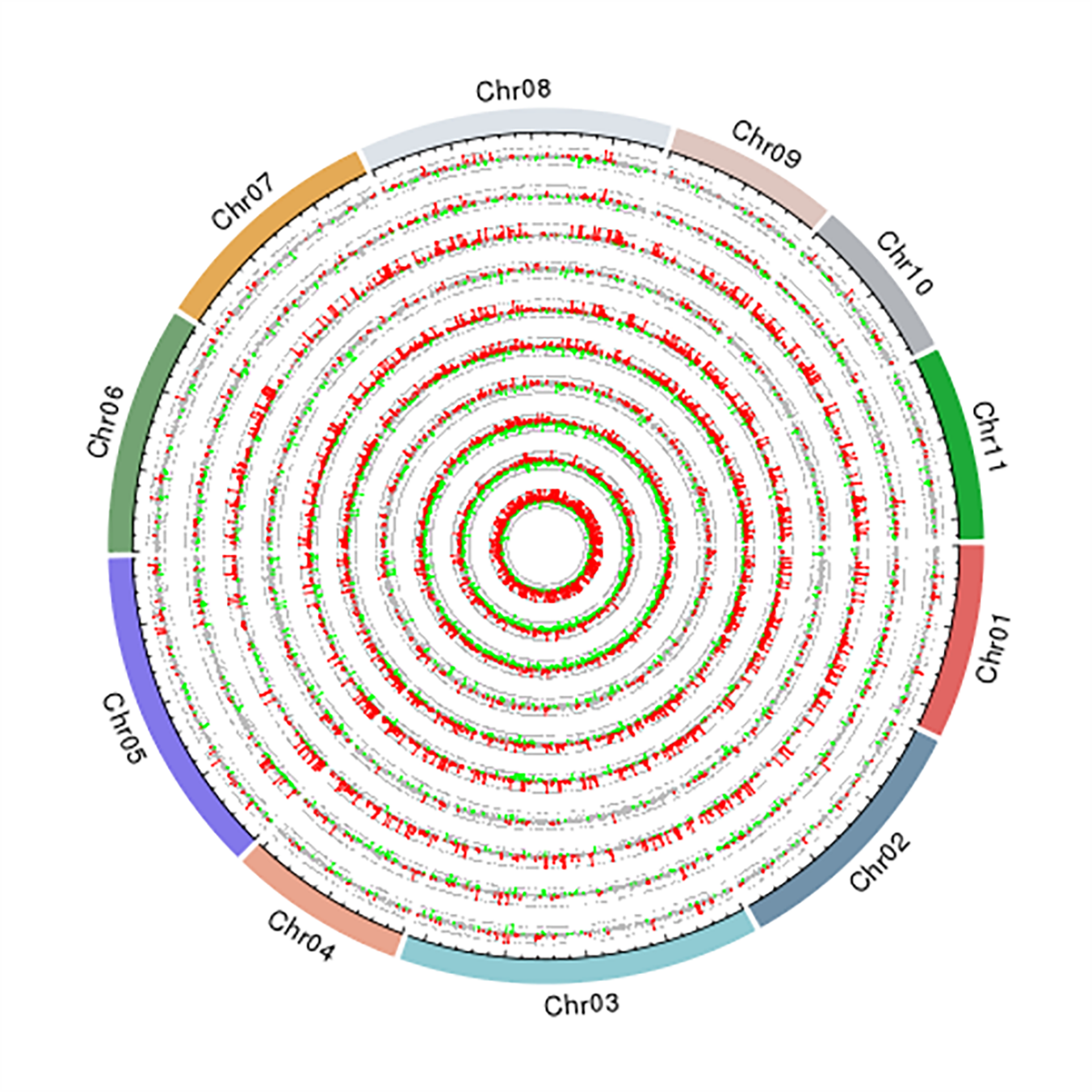
Fig. B：Genome circle map

Note: The outermost circle is the chromosome band, from the outside to the inside are the differential expression analysis results of different differential analysis. Red and green are the histograms of the log2FoldChange values of the up-regulated and down-regulated genes, and the gray is the scatter plot of the log2FoldChange values of the undifferentially expressed genes.
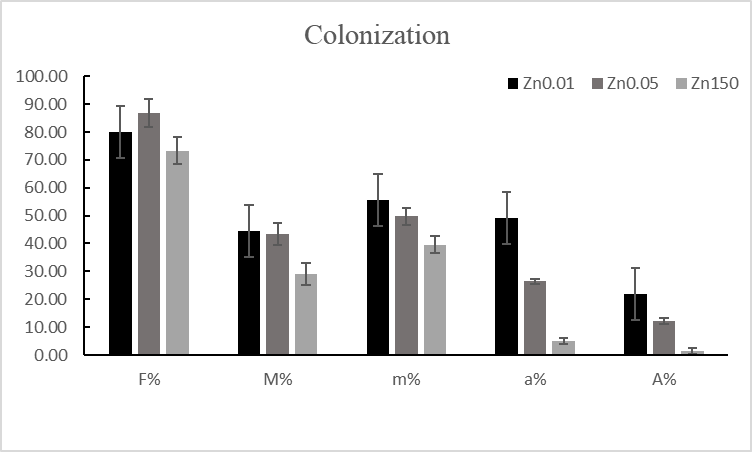

Supplement: Supplementary file 4 — Additional file 4. [file 12870_2022_3456_MOESM4_ESM.docx]
