# Supplementary material for: Transcriptional regulation of metal metabolism- and nutrient absorption-related genes in Eucalyptus grandis by arbuscular mycorrhizal fungi at different zinc concentrations
Source: BMC Plant Biol. 2022 Feb 22;22:76. doi: 10.1186/s12870-022-03456-5 (PMC8862258; doi:10.1186/s12870-022-03456-5)
Supplement: Supplementary file 5 — Additional file 5. [file 12870_2022_3456_MOESM5_ESM.docx]

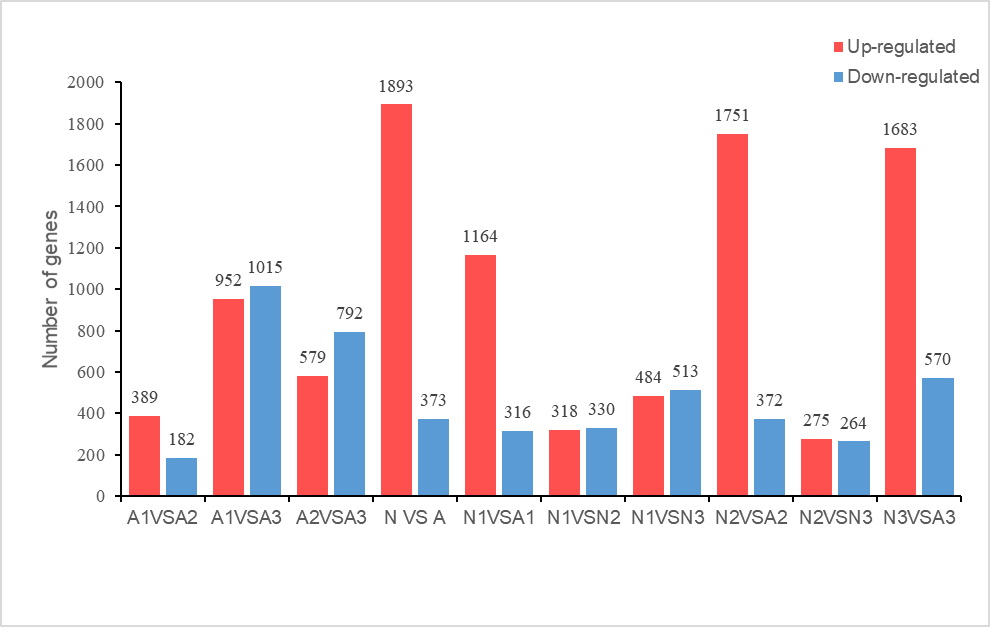
Supplementary Figure S1. Differentially expressed genes under different Zn level Note: The abscissa indicates the comparison group for difference analysis, and the ordinate indicates the number of differential genes. The red in the color indicates the up-regulated gene, and the blue indicates the down-regulated gene. (N represent NM (N1, N2, N3), A represent AM (A1, A2, A3), A1 represent AM 0.01 μM Zn, A2 represent AM 0.5 μM Zn, A3 represent AM 150 μM Zn, N1 represent NM 0.01 μM Zn, N2 represent NM 0.5 μM Zn, N3 represent NM 150 μM Zn,).


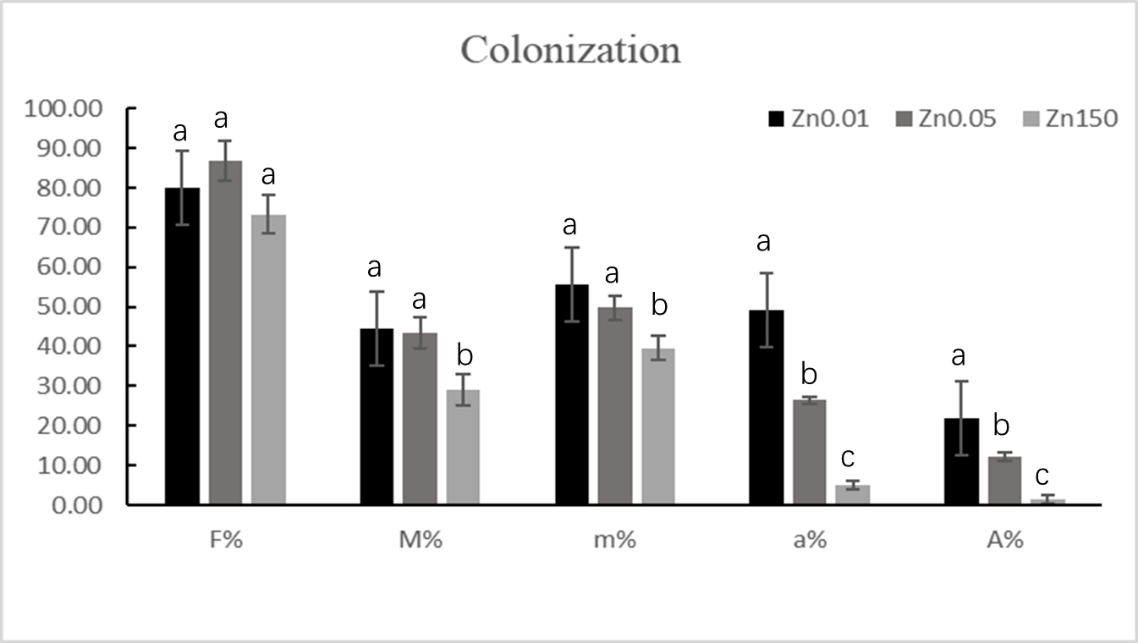


Supplementary Figure S2. Arbuscular mycorrhizal colonization rate of *Eucalyptus grandis*. (F%: Frequency of mycorrhiza in the root system, M%: Idensity of the mycorrhizal colonization in the root system, m%: M*(nb total) / (nb myco), a%: Arbuscule abundance in mycorrhizal parts of root fragmengs, A%: Arbuscule abundance in the root system).


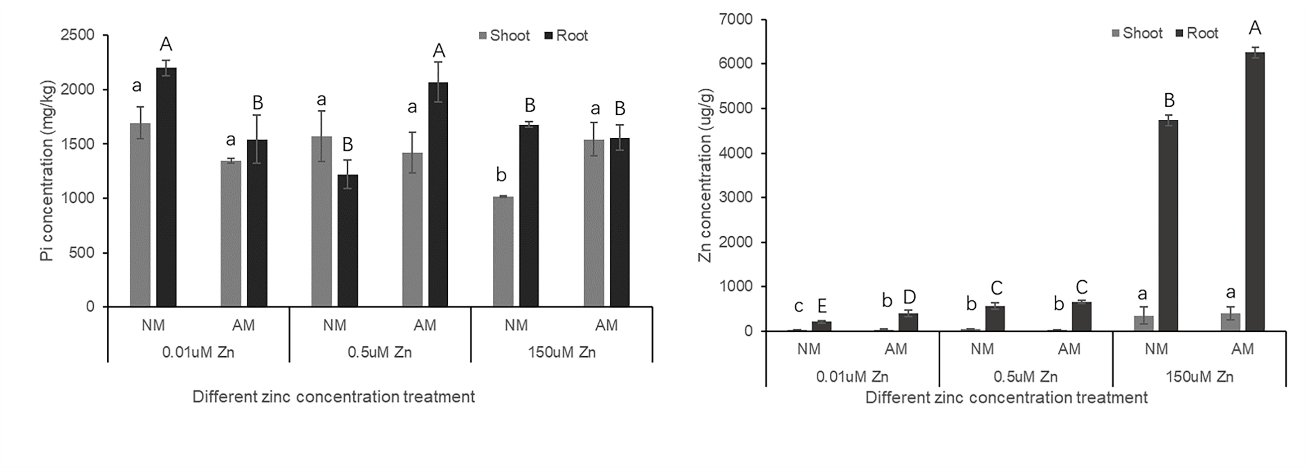
Supplementary Figure S3. Determination of Zn/Pi in Mycorrhizal *Eucalyptus grandis*


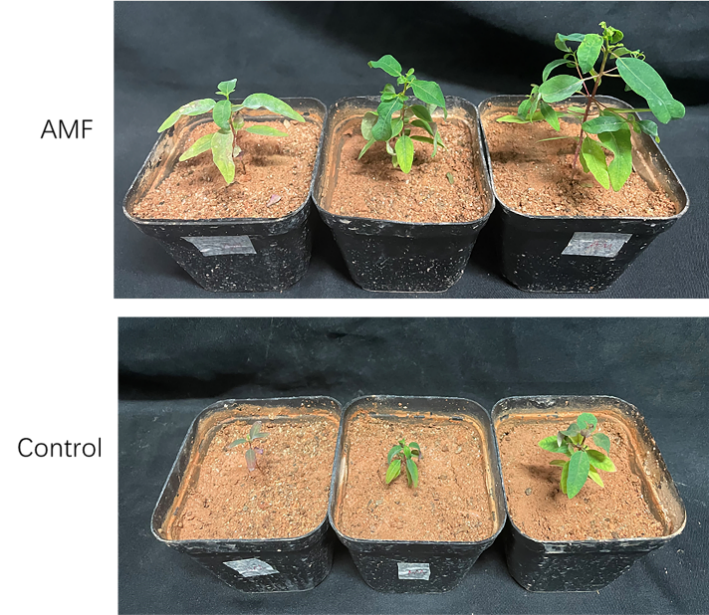


Supplementary Figure S4. Mycorrhizal and non-mycorrhizal *Eucalyptus grandis*（Pre-experiment result graph, Soil taken from South China）


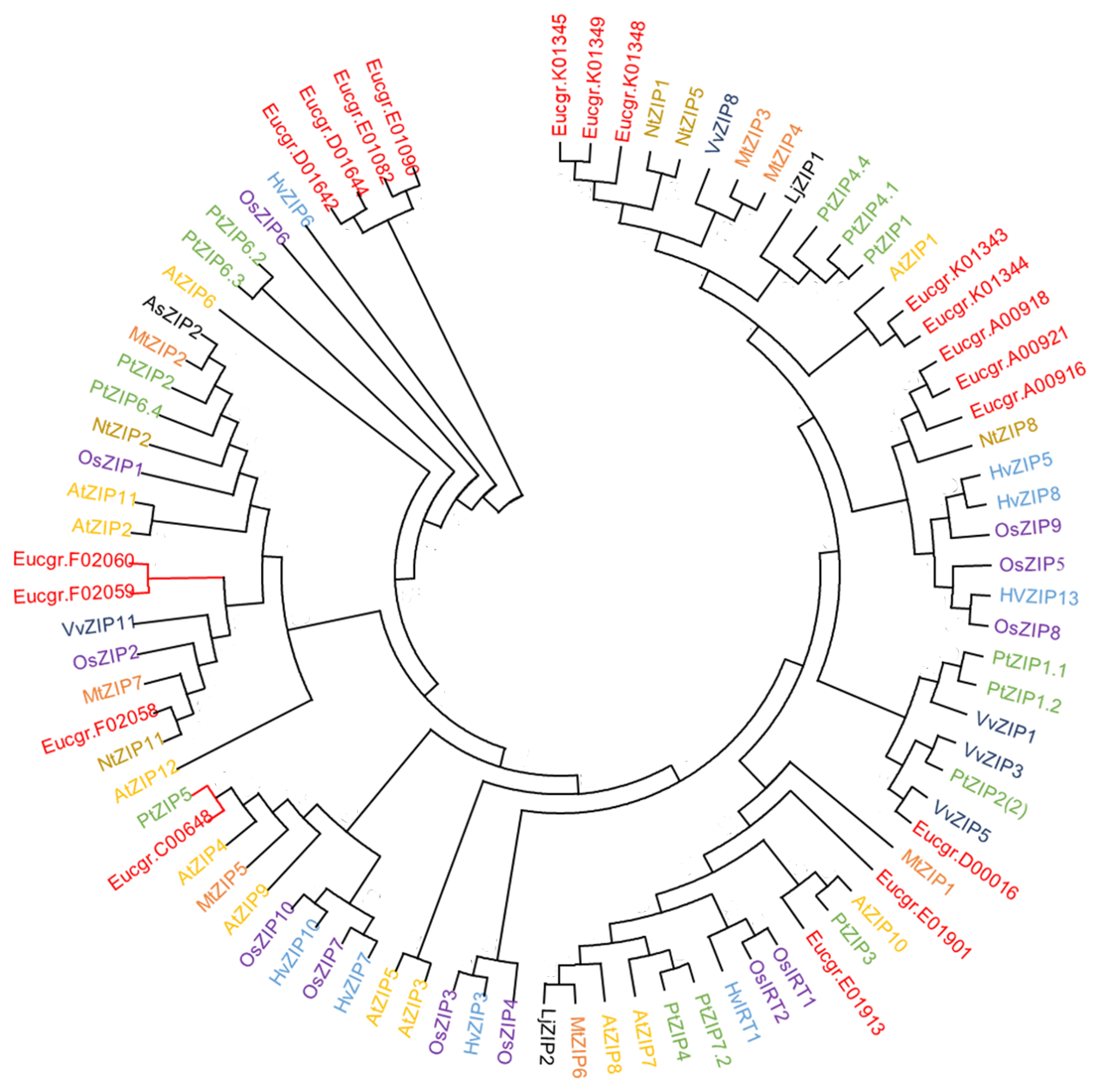


Supplementary Figure S5. Phylogenetic analysis of the ZNT family members. Phylogenetic tree of ZIP proteins from ten plant species. ZIP proteins of ten plant species were used for construction of the phylogenetic tree using MEGA6. The ten species are *Arabidopsis thaliana* (*At*), *Oryza sativa* (*Os*), *Hordeum vulgare* (*Hv*), *Populus trichocarpa* (Pt), *Vitis vinifera* (Vv), *Medicago Sativa* (*Mt*), *Nicotiana Tabacum L.* (*Nt*)*, Lotus japonicus* (*Lj*), *Astragalus sinicu* (*As*) and *Eucalyptus grandis* (*Eg*) respectively. Accession numbers and identifier of the predicted proteins are listed in Supporting Information.


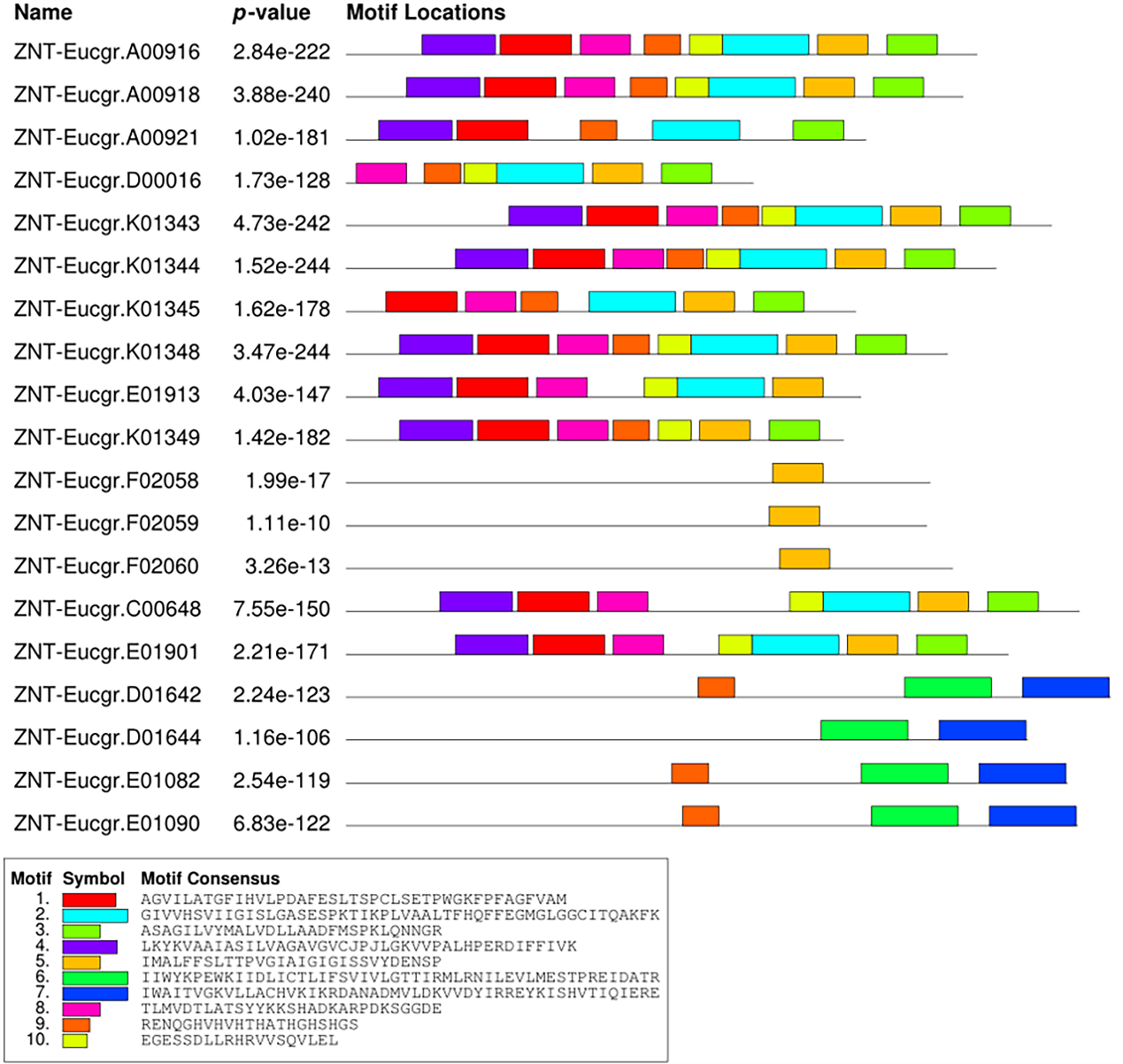


Supplementary Figure S6. The motif of ZNT family members. The motif analyzed using MEME (https://meme-suite.org/meme/tools/meme).


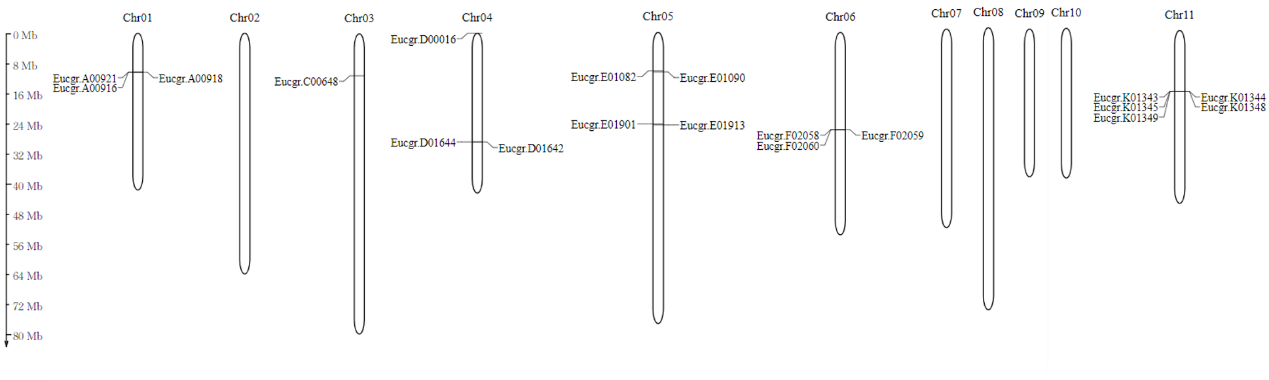


Supplementary Figure S7. The Chromosomal location of ZNT family genes in *E.grandis* using MG2C (http://mg2c.iask.in/mg2c_v2.1/).


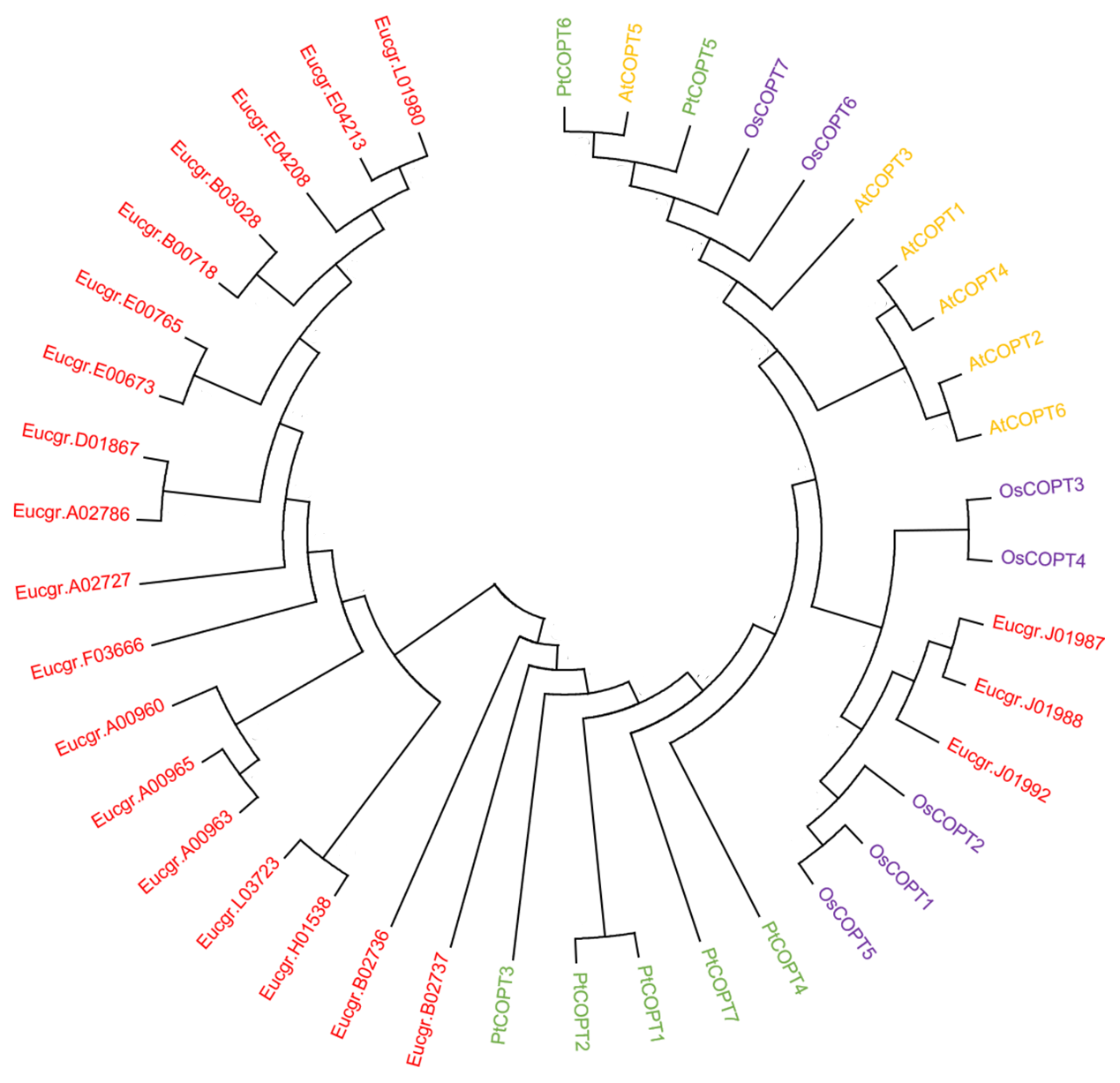


Supplementary Figure S8. Phylogenetic analysis of the COPT/Ctr family members. COPT/Ctr proteins of four plant species were used for construction of the phylogenetic tree using MEGA6. The four species are *Arabidopsis thaliana* (*At*), *Oryza sativa* (*Os*), *Populus trichocarpa* (*Pt*), and *Eucalyptus grandis* (*Eg*) respectively. Accession numbers and identifier of the predicted proteins are listed in Supporting Information


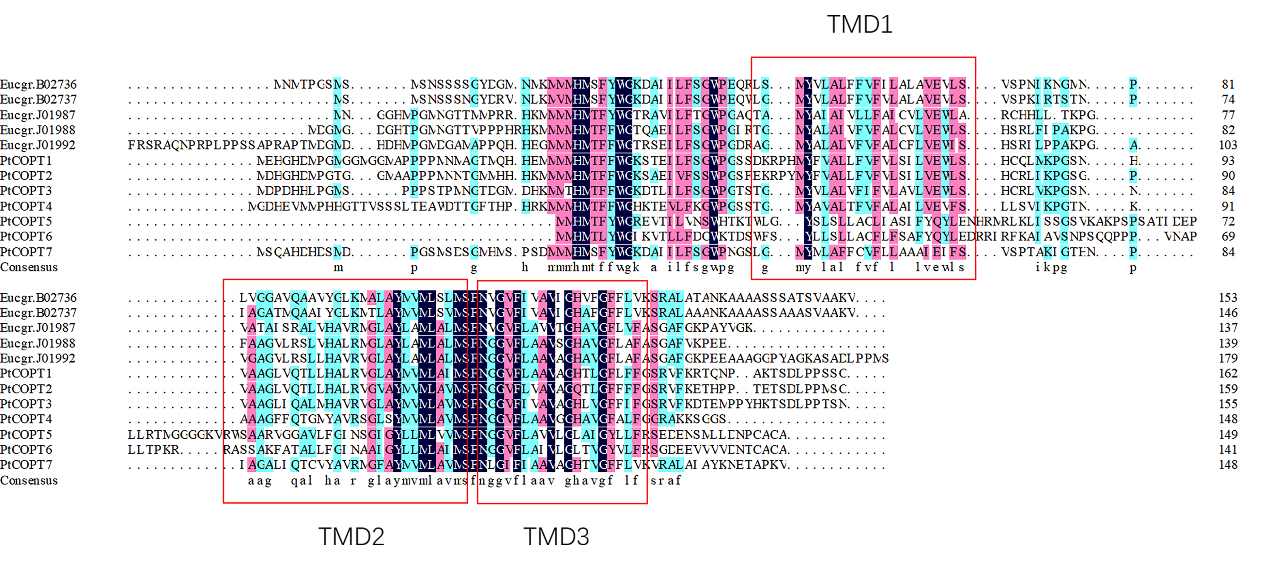


supplementary Figure S9. The TM motif of COPT/Ctr family members.


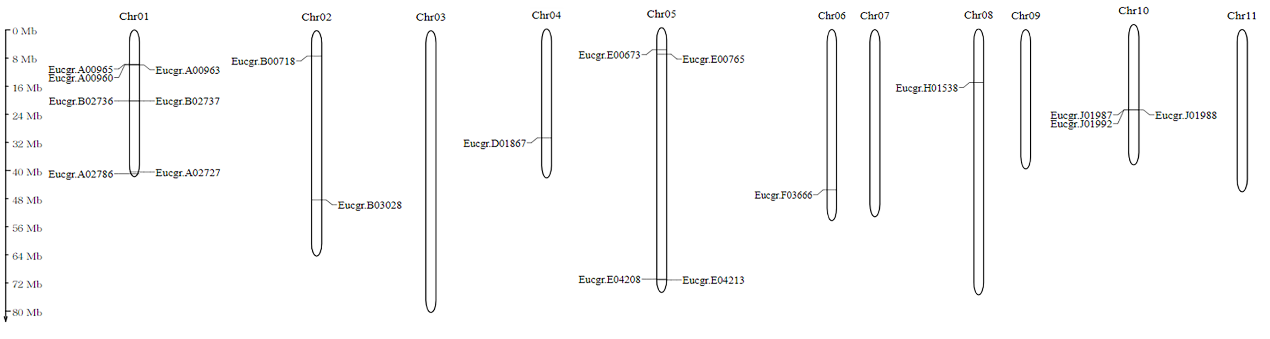


Supplementary Figure S10. The Chromosomal location of COPT/Ctr family genes in *E.grandis* using MG2C (http://mg2c.iask.in/mg2c_v2.1/).


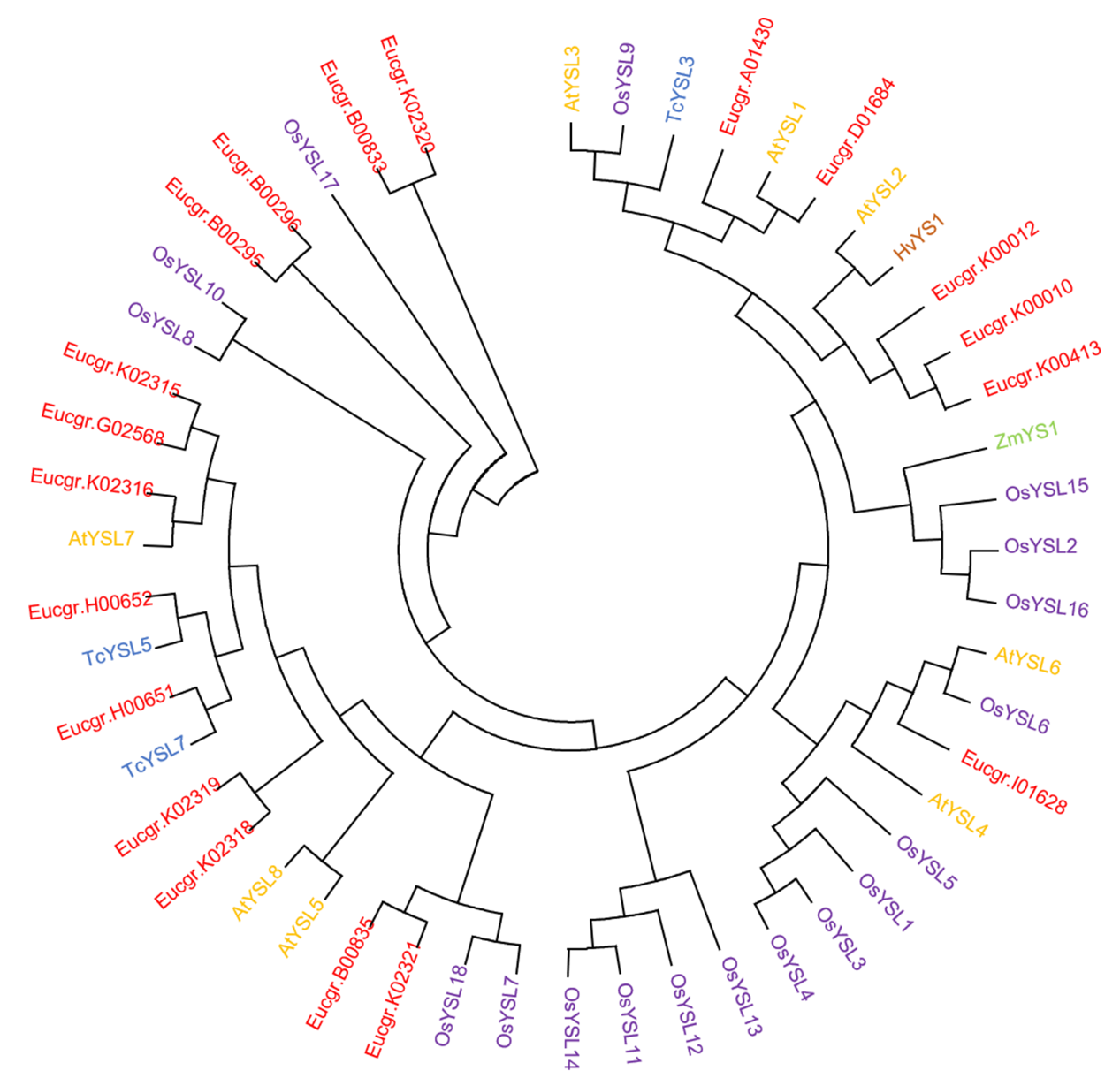


Supplementary Figure S11. Phylogenetic analysis of the YSL family members. Phylogenetic tree of YSL proteins from five plant species. YSL proteins of five plant species were used for construction of the phylogenetic tree using MEGA6. The five species are *Arabidopsis thaliana* (*At*), *Oryza sativa* (*O*s), *Thlaspi caerulescens* (*Tc*), *Zea mays* (*Zm*), and *Eucalyptus grandis* (*Eg*) respectively. Accession numbers and identifier of the predicted proteins are listed in Supporting Information.


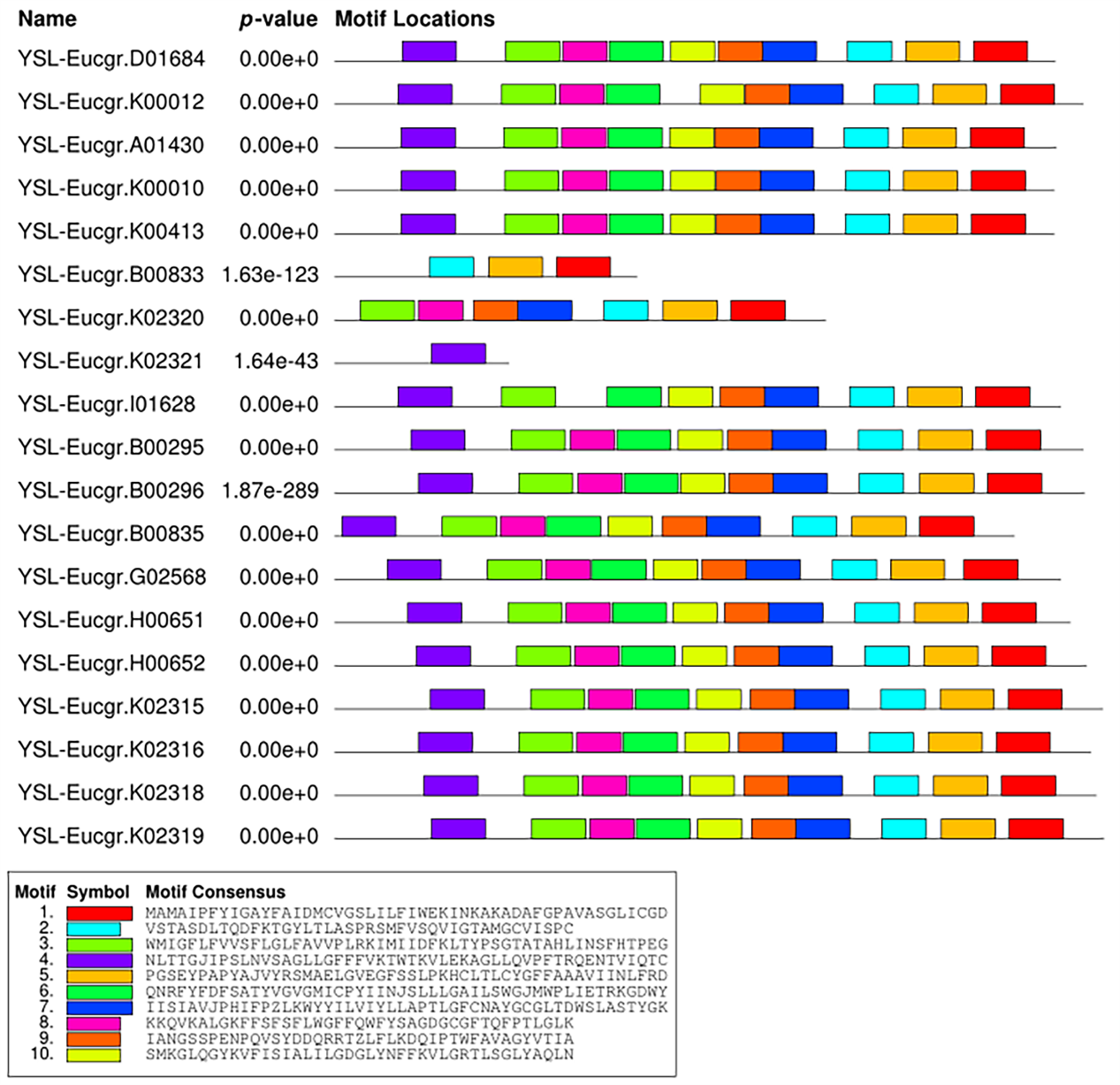


Supplementary Figure S12. The motif of YSL family members. The motif analyzed using MEME (https://meme-suite.org/meme/tools/meme).


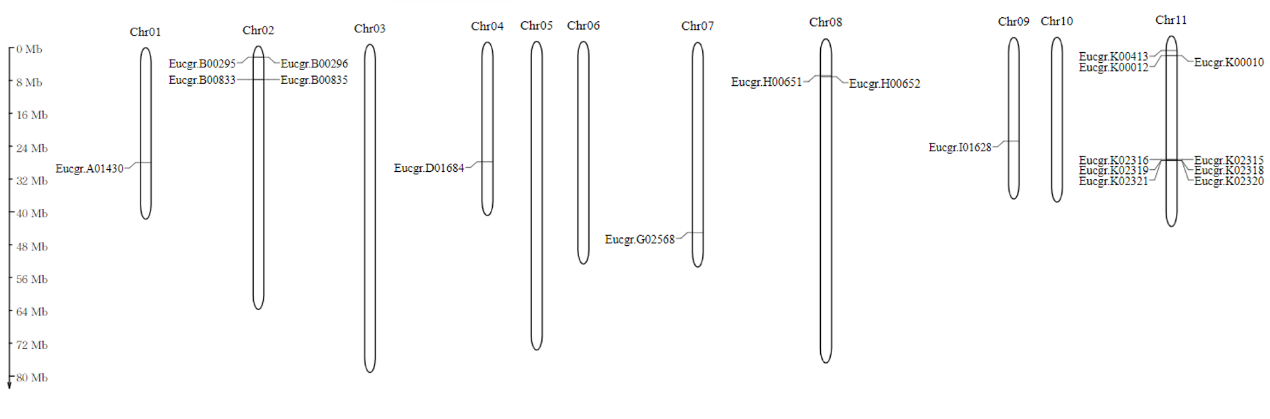


Supplementary Figure S13. The Chromosomal location of COPT/Ctr family genes in *E.grandis* using MG2C (http://mg2c.iask.in/mg2c_v2.1/).


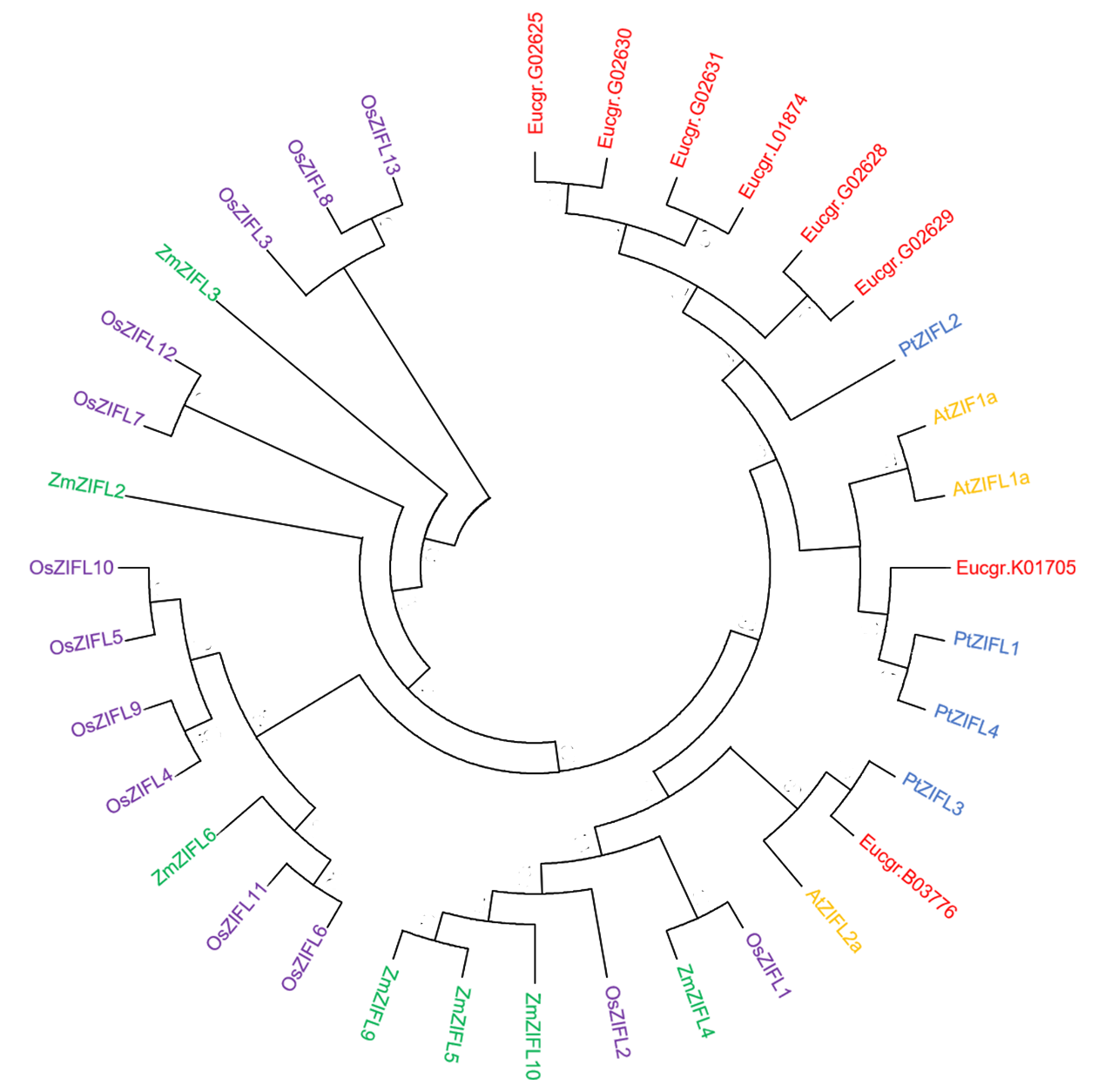


Supplementary Figure S14. Phylogenetic analysis of the ZIFL family members. Phylogenetic tree of ZIFL proteins from five plant species. ZIFL proteins of five plant species were used for construction of the phylogenetic tree using MEGA6. The five species are *Arabidopsis thaliana* (*At*), *Oryza sativa* (*Os*), *Populus trichocarpa* (*Pt*), *Zea mays* (*Zm*), and *Eucalyptus grandis* (*Eg*) respectively. Accession numbers and identifier of the predicted proteins are listed in Supporting Information.


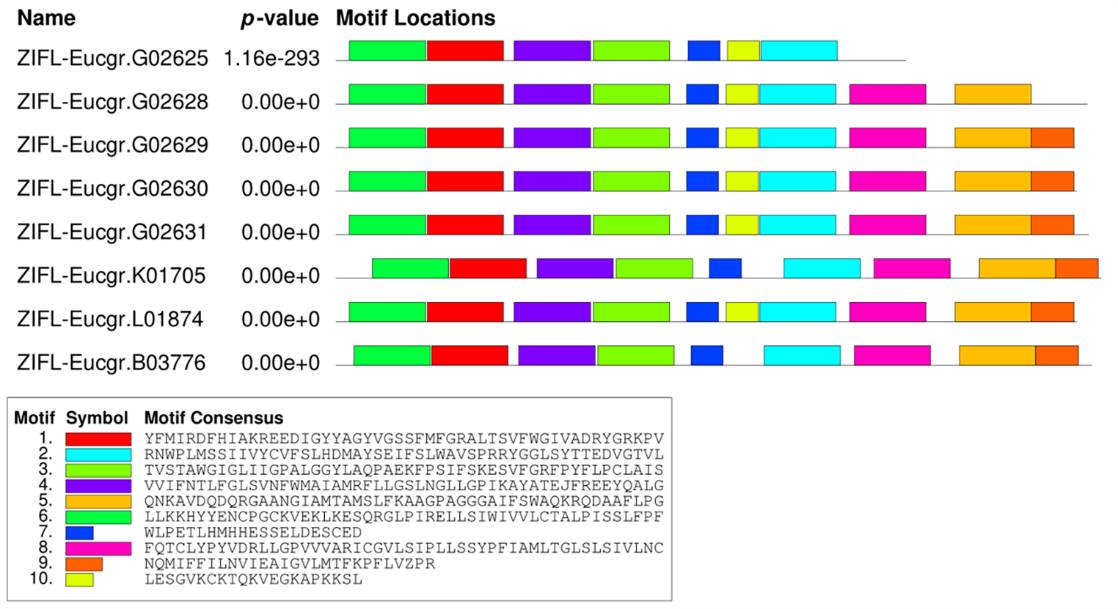


Supplementary Figure S15. The motif of ZIFL family members. The motif analyzed using MEME (https://meme-suite.org/meme/tools/meme).


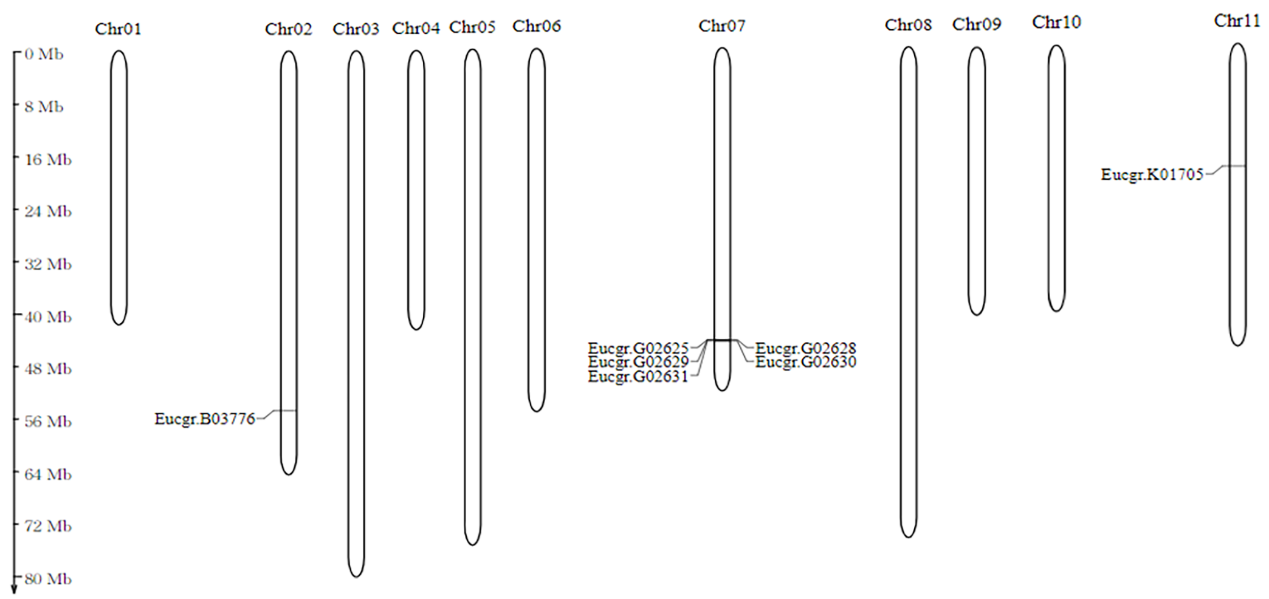


Supplementary Figure S16. The Chromosomal location of COPT/Ctr family genes in *E.grandis* using MG2C (http://mg2c.iask.in/mg2c_v2.1/).


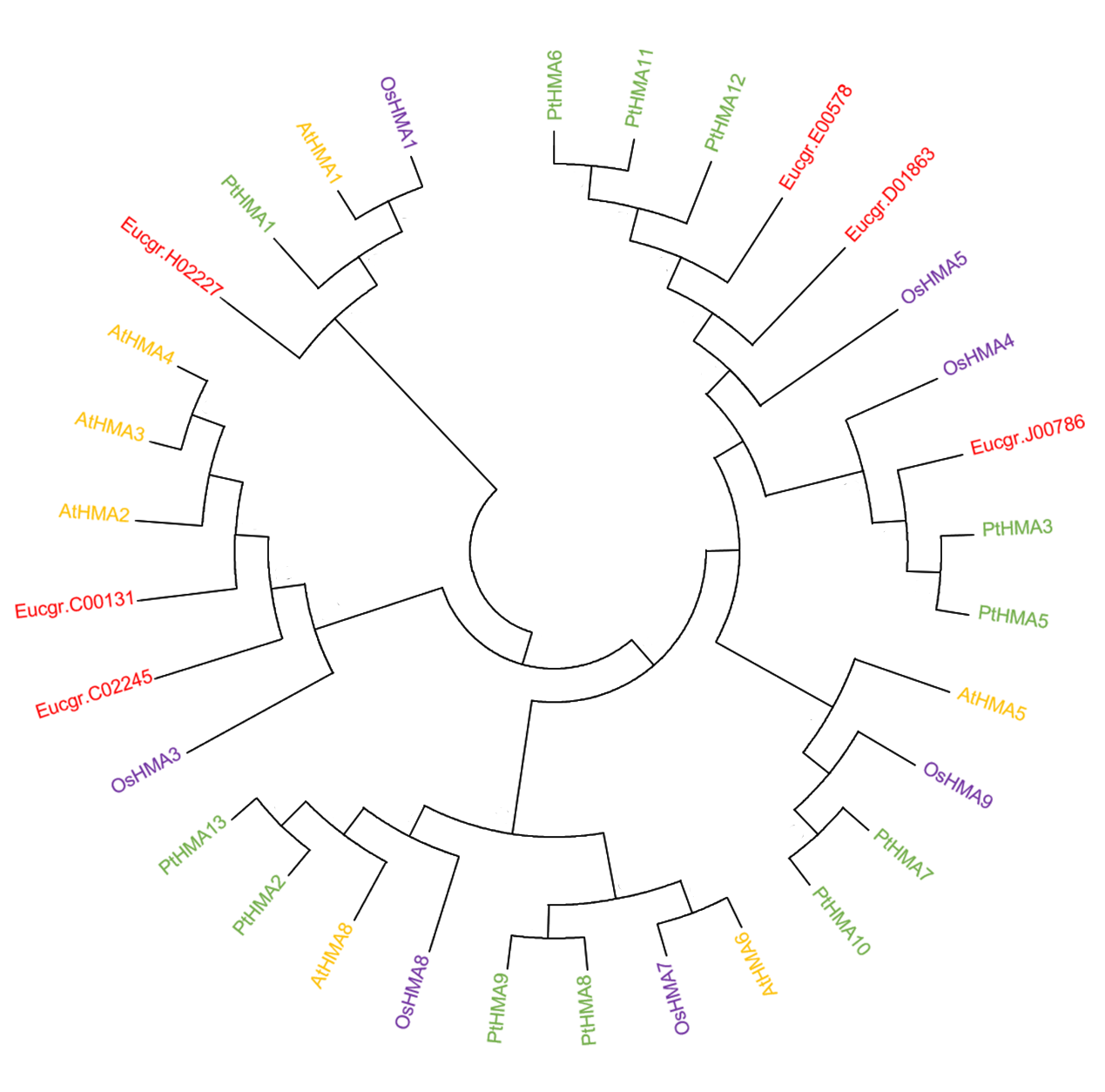


Supplementary Figure S17. Phylogenetic analysis of the HMA family members. Phylogenetic tree of HMA proteins from four plant species. HMA proteins of four plant species were used for construction of the phylogenetic tree using MEGA6. The four species are *Arabidopsis thaliana* (*At*), *Oryza sativa* (*Os*), *Populus trichocarpa* (*Pt*), and *Eucalyptus grandis* (*Eg*) respectively. Accession numbers and identifier of the predicted proteins are listed in Supporting Information.


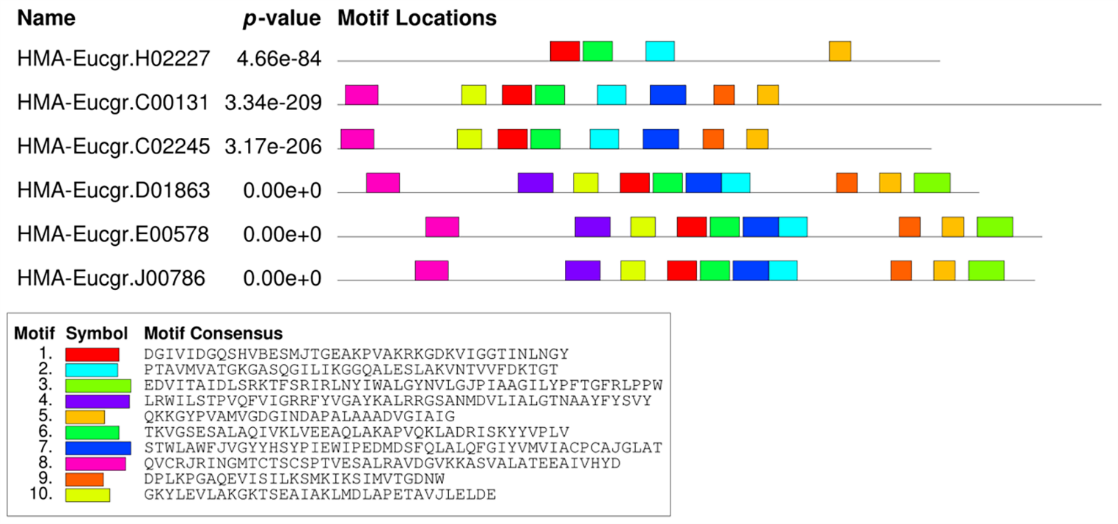


Supplementary Figure S18. The motif of HMA family members. The motif analyzed using MEME (https://meme-suite.org/meme/tools/meme).


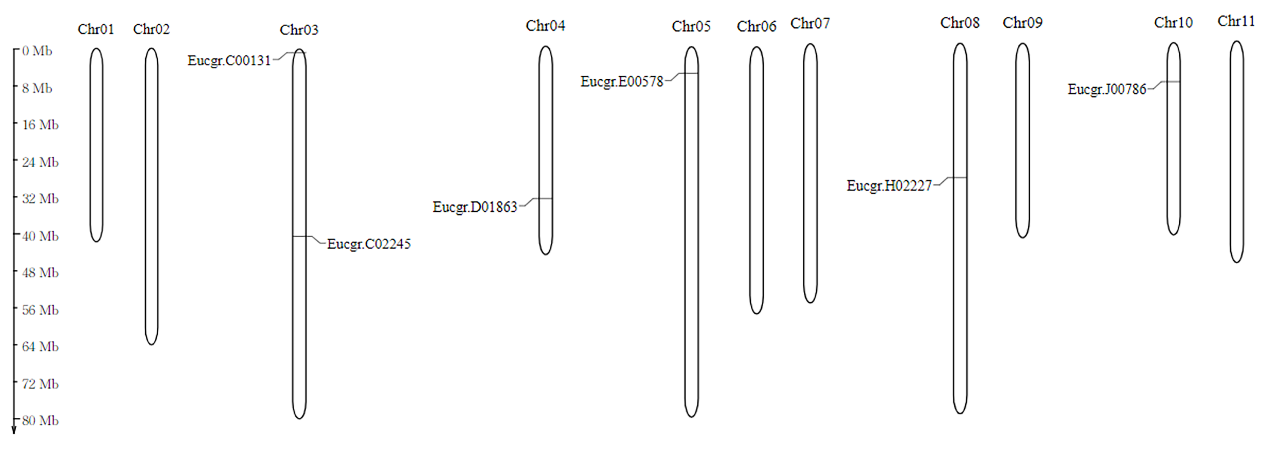


Supplementary Figure S19. The Chromosomal location of COPT/Ctr family genes in *E.grandis* using MG2C (http://mg2c.iask.in/mg2c_v2.1/).


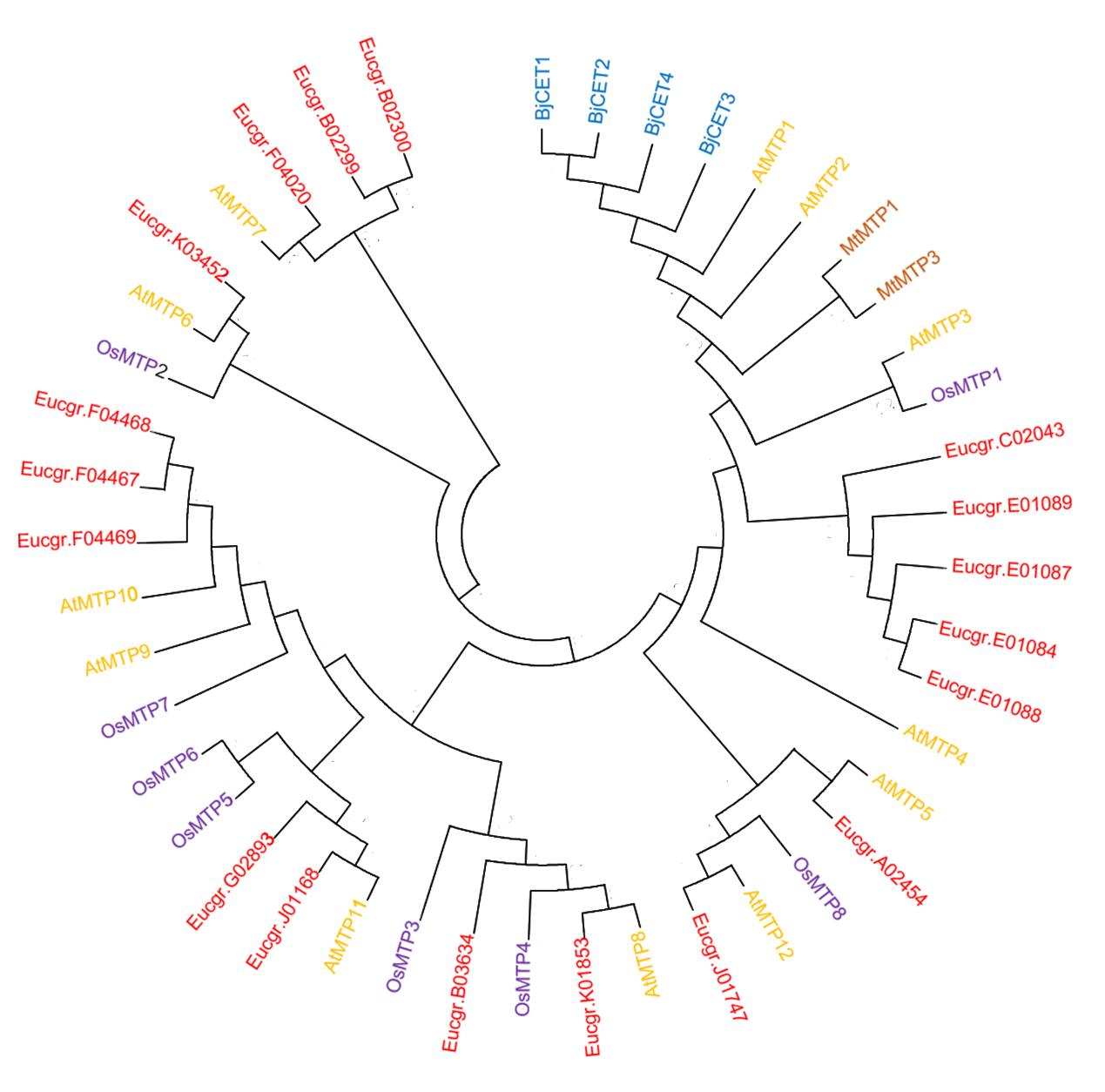


Supplementary Figure S20. Phylogenetic analysis of the CE and MTP family members. Phylogenetic tree of CE and MTP proteins from six plant species. CE and MTP proteins of ten plant species were used for construction of the phylogenetic tree using MEGA6. The six species are *Arabidopsis thaliana* (*At*), *Oryza sativa* (*Os*), *Medicago Sativa* (*Mt*), *Brassica juncea* (*Bj*), and *Eucalyptus grandis* (*Eg*) respectively. Accession numbers and identifier of the predicted proteins are listed in Supporting Information.


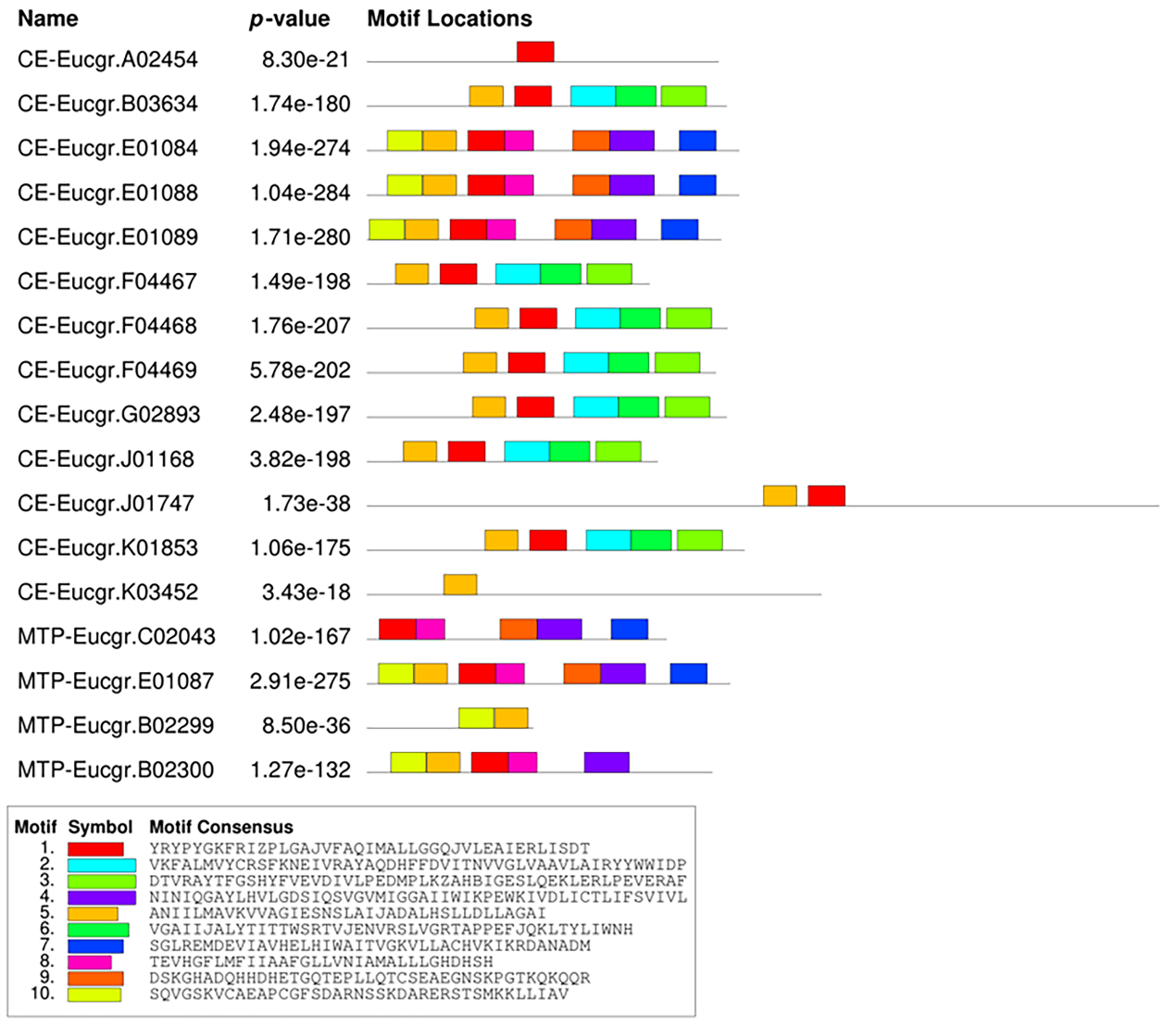


Supplementary Figure S21. The motif of CE/MTP family members. The motif analyzed using MEME (https://meme-suite.org/meme/tools/meme).


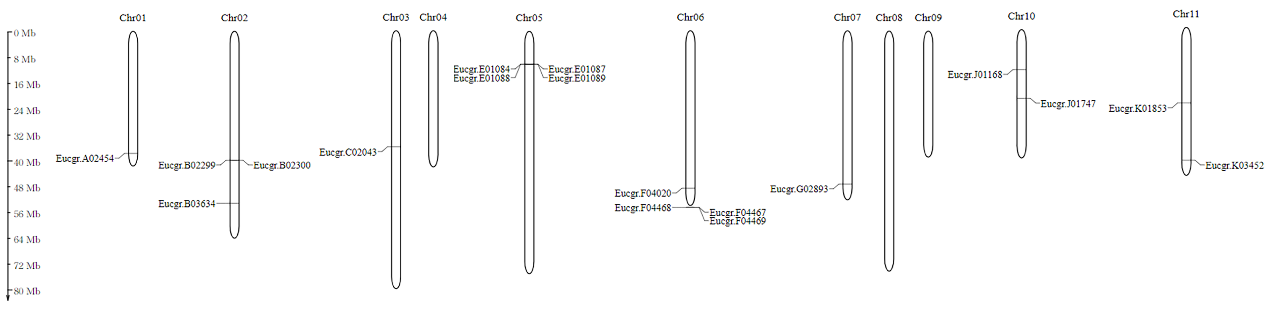


Supplementary Figure S22. The Chromosomal location of CE/MTP family genes in *E.grandis* using MG2C (http://mg2c.iask.in/mg2c_v2.1/).


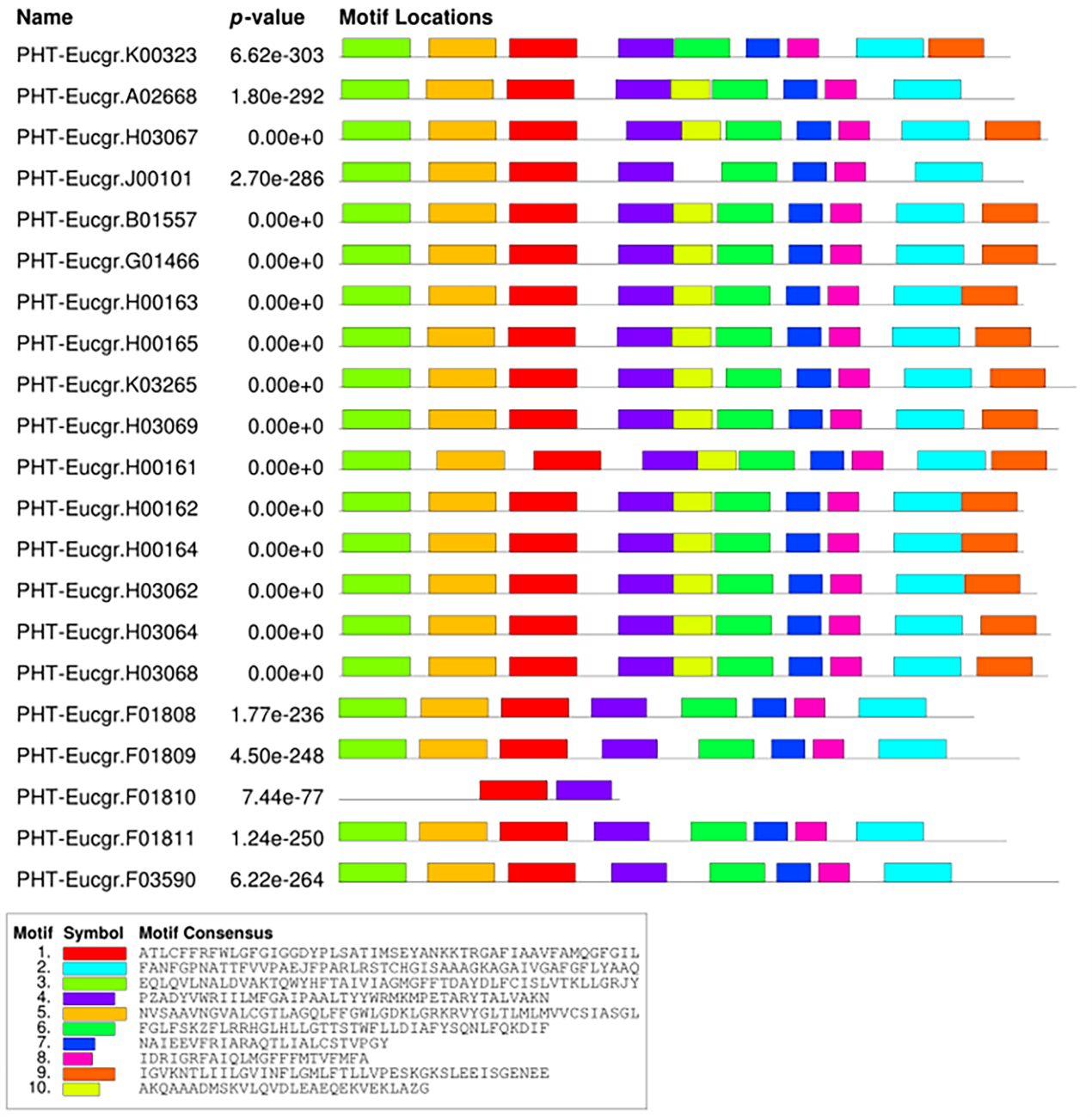


Supplementary Figure S23. The motif of CE/MTP family members. The motif analyzed using MEME (https://meme-suite.org/meme/tools/meme).


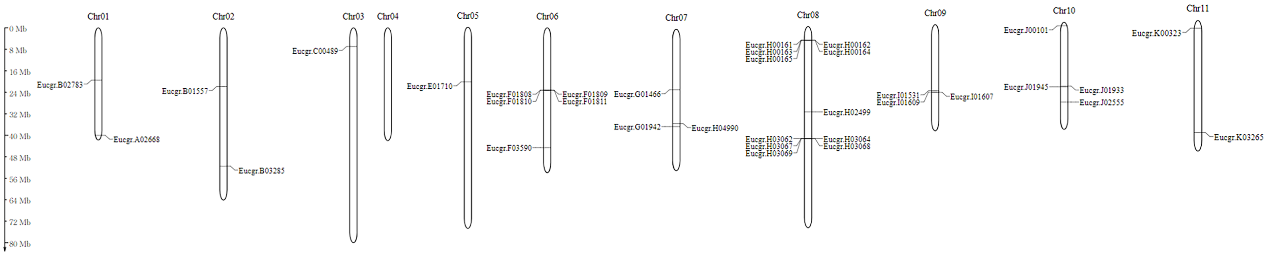


Supplementary Figure S24. The Chromosomal location of PHT family genes in *E.grandis* using MG2C (http://mg2c.iask.in/mg2c_v2.1/).


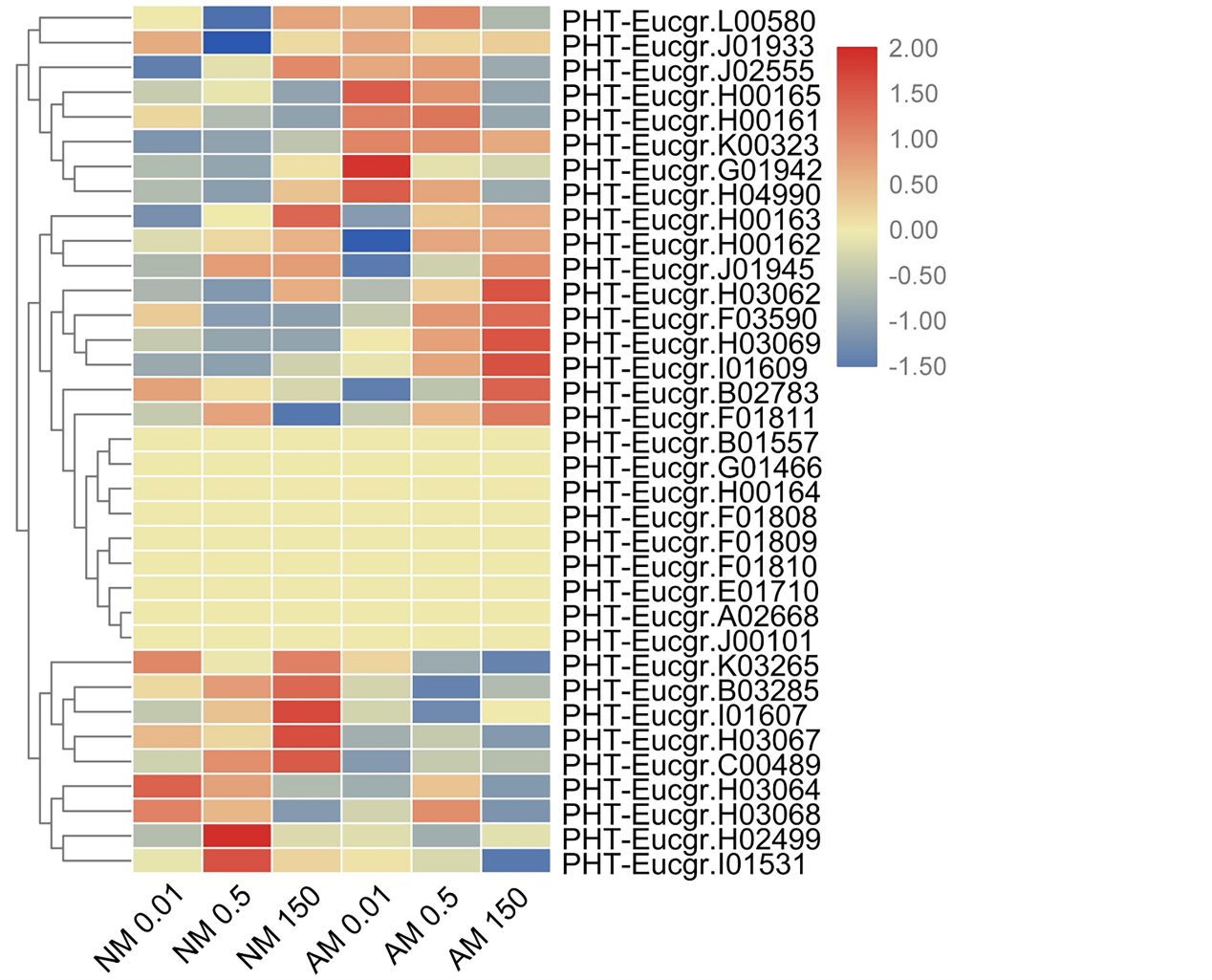


Supplementary Figure S25. Heatmap of PHT family members. Cluster analysis of transcriptional fold-changes of PHT family gens in roots of non-mycorrhizal and mycorrhizal *E. grandis* exposed to 0.01, 0.5, or 150μM ZnCl_2_. The color scale indicates fold-changes of mRNAs. For each gene, the expression levels in non-mycorrhizal roots exposed to 0.5μM ZnCl_2_ were defined as 1, and the corresponding fold-changes under 0.01 and 150μM ZnCl_2_ were calculated.


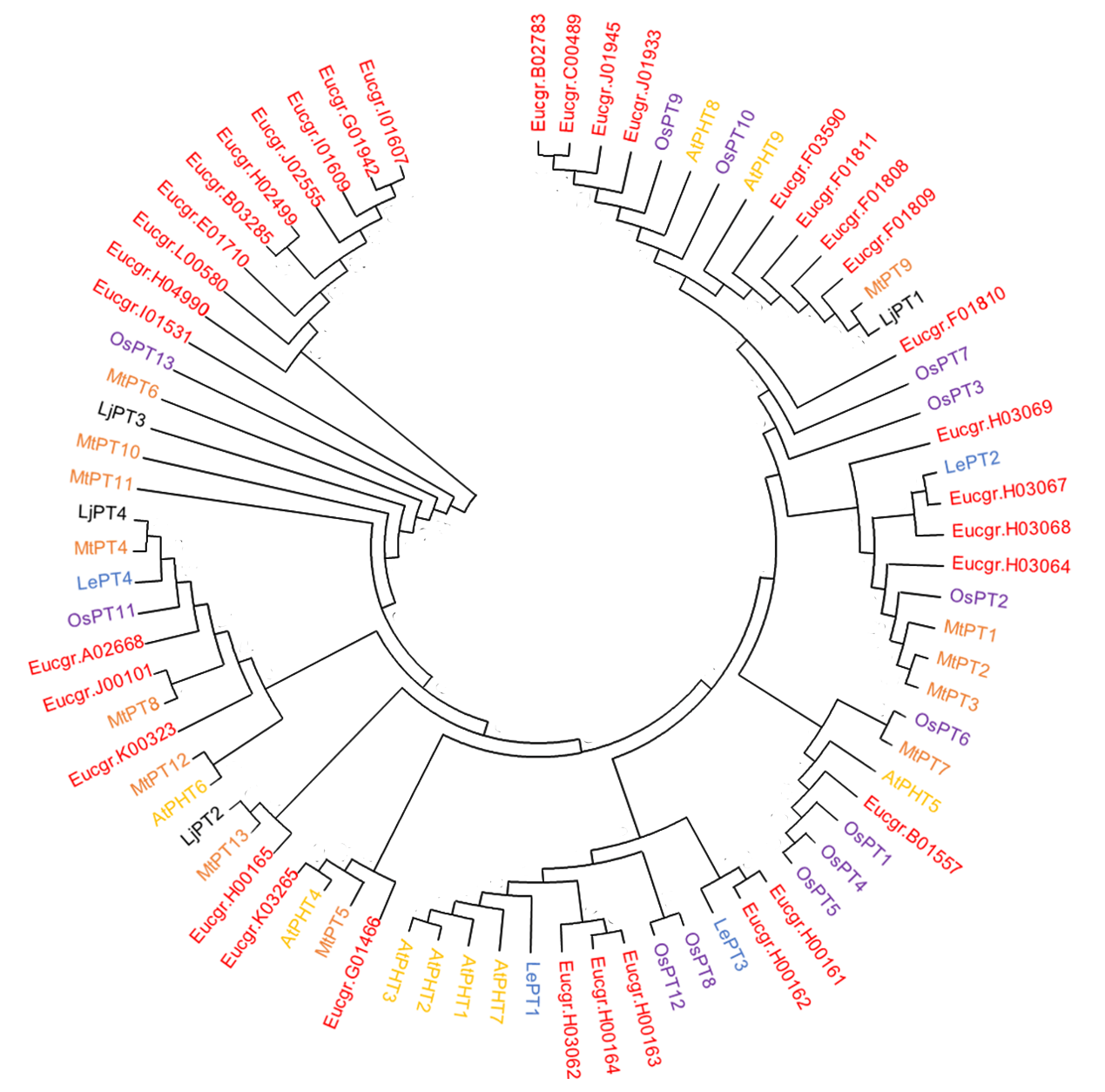


Supplementary Figure S26. Phylogenetic analysis of the PHT family members from various species.


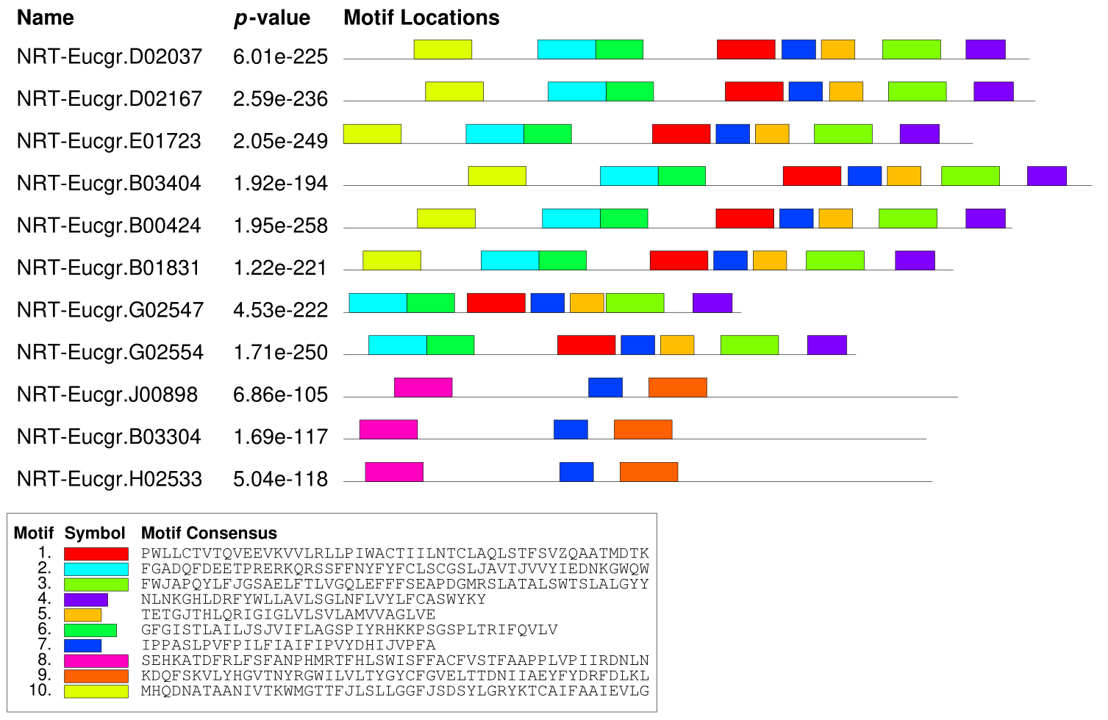

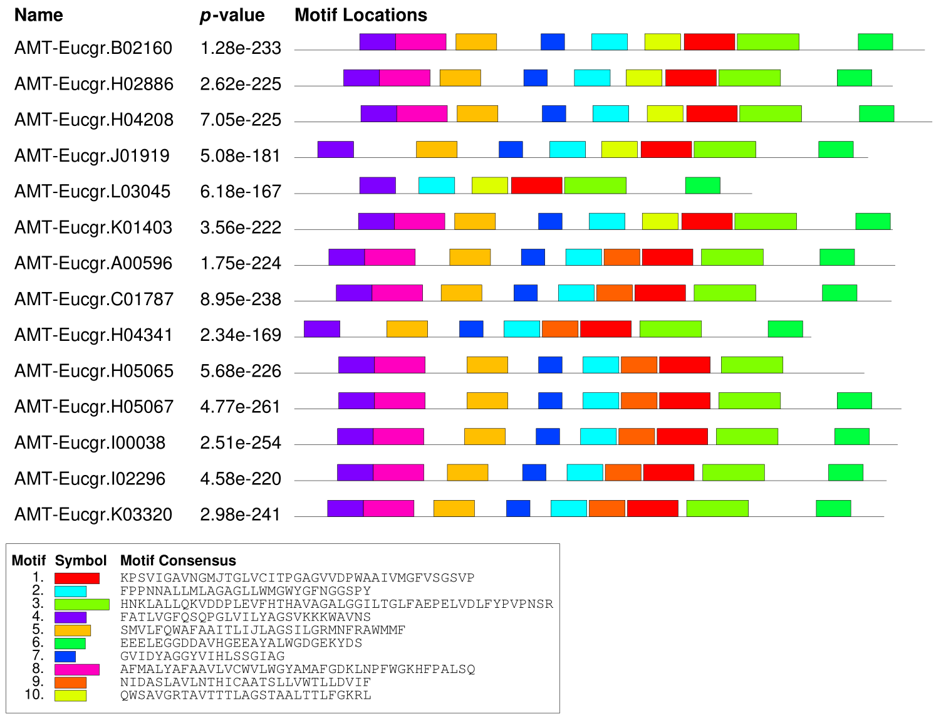


Supplementary Figure S27. The motif of AMT and NRT family members. The motif analyzed using MEME (https://meme-suite.org/meme/tools/meme).


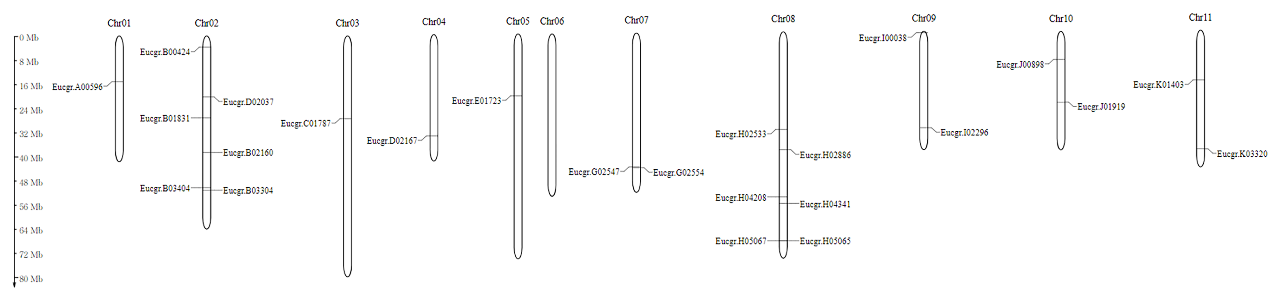


Supplementary Figure S28. The Chromosomal location of AMT and NRT family genes in *E.grandis* using MG2C (http://mg2c.iask.in/mg2c_v2.1/).


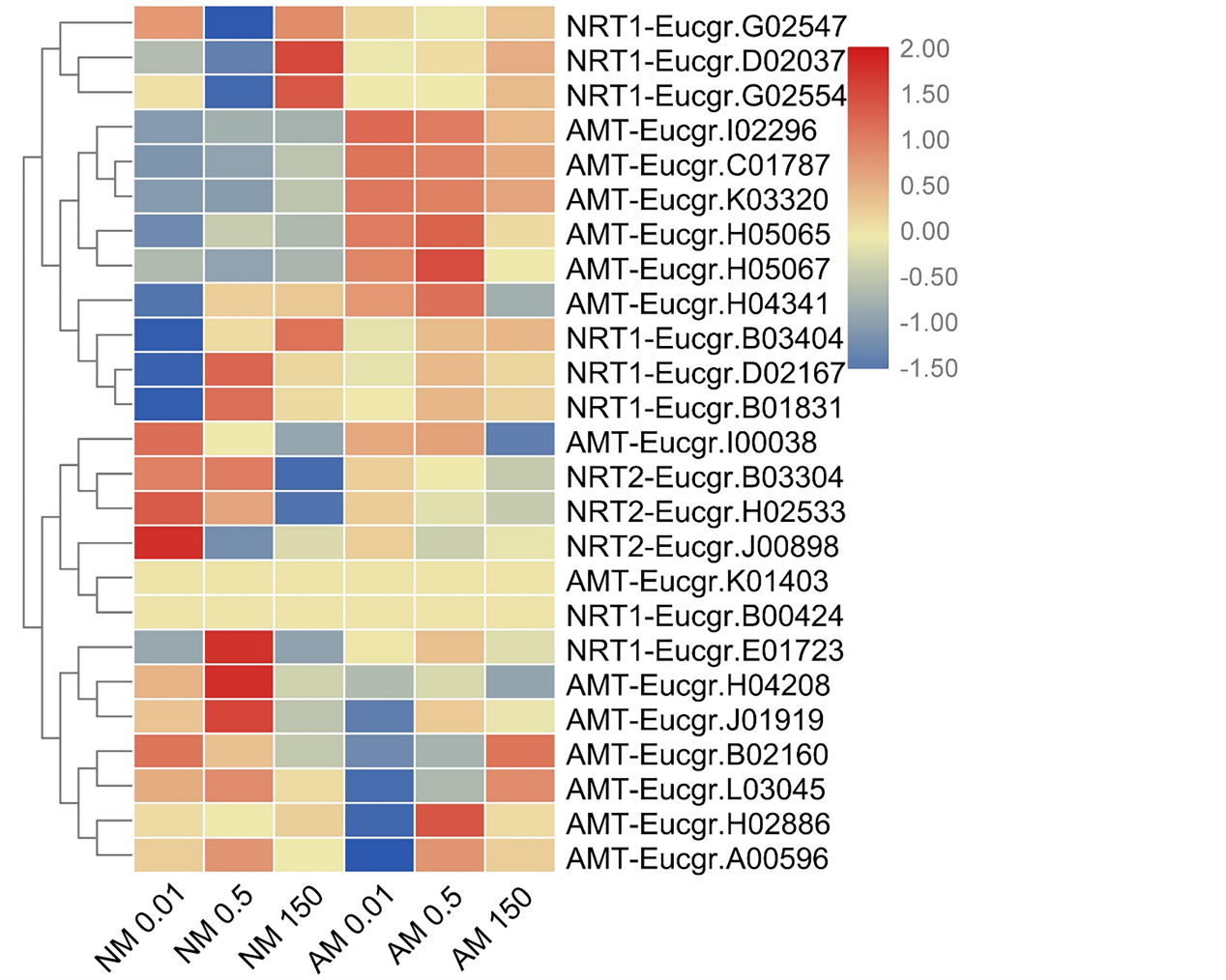


Supplementary Figure S29. Heatmap of AMT and NRT family members. Cluster analysis of transcriptional fold-changes of AMT and NRT family gens in roots of non-mycorrhizal and mycorrhizal *E. grandis* exposed to 0.01, 0.5, or 150μM ZnCl_2_. The color scale indicates fold-changes of mRNAs. For each gene, the expression levels in non-mycorrhizal roots exposed to 0.5μM ZnCl_2_ were defined as 1, and the corresponding fold-changes under 0.01 and 150μM ZnCl_2_ were calculated.


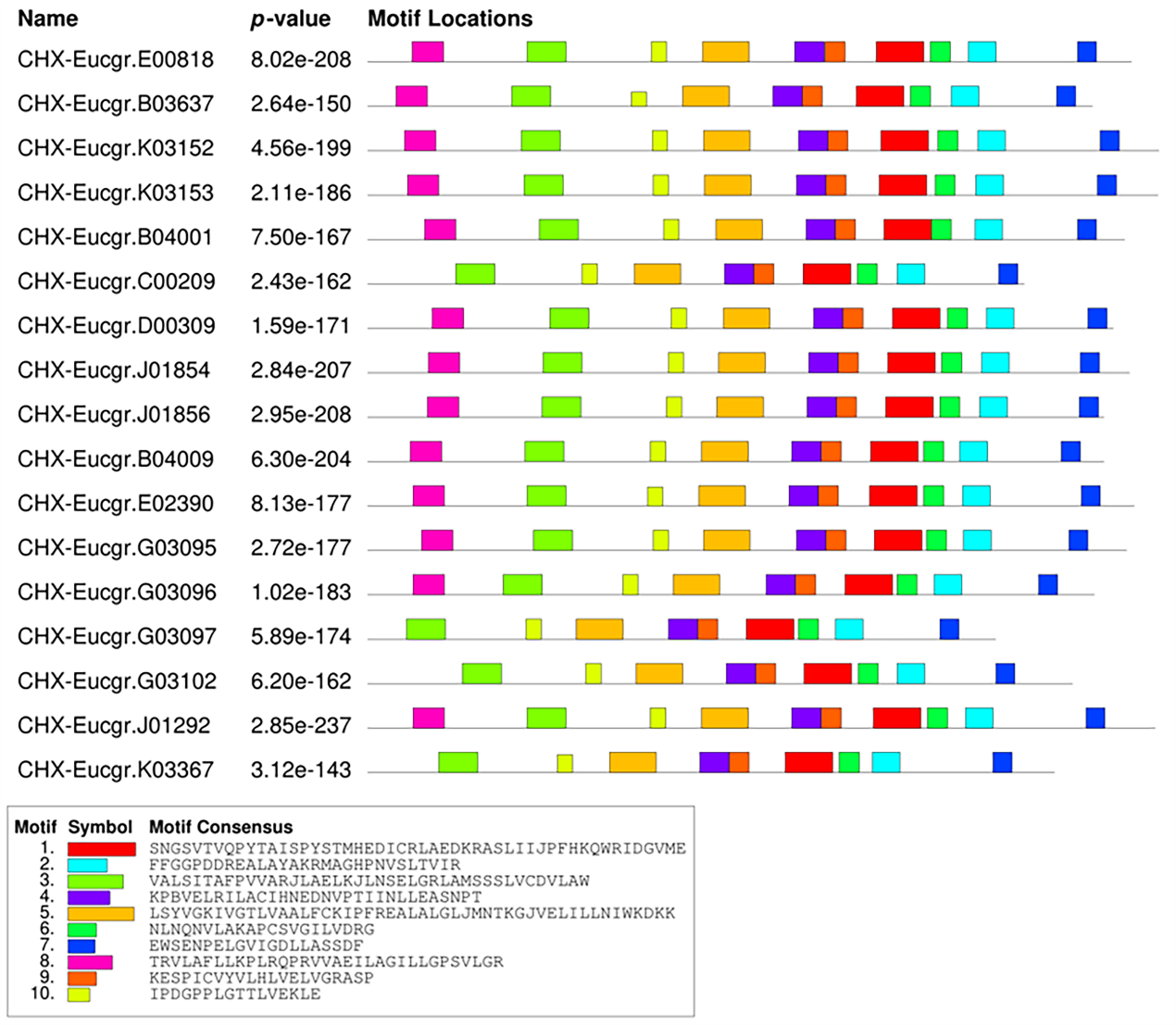

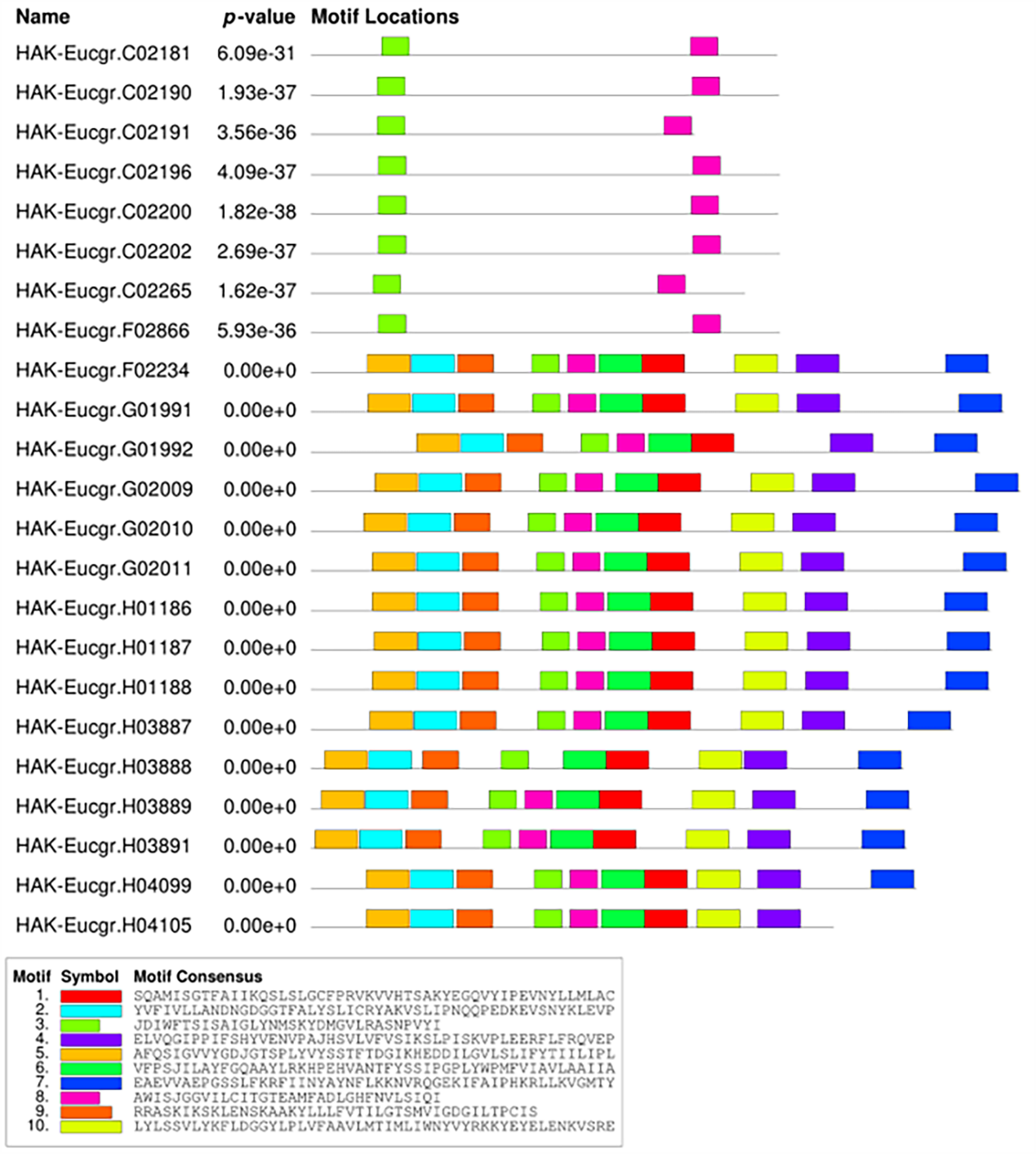


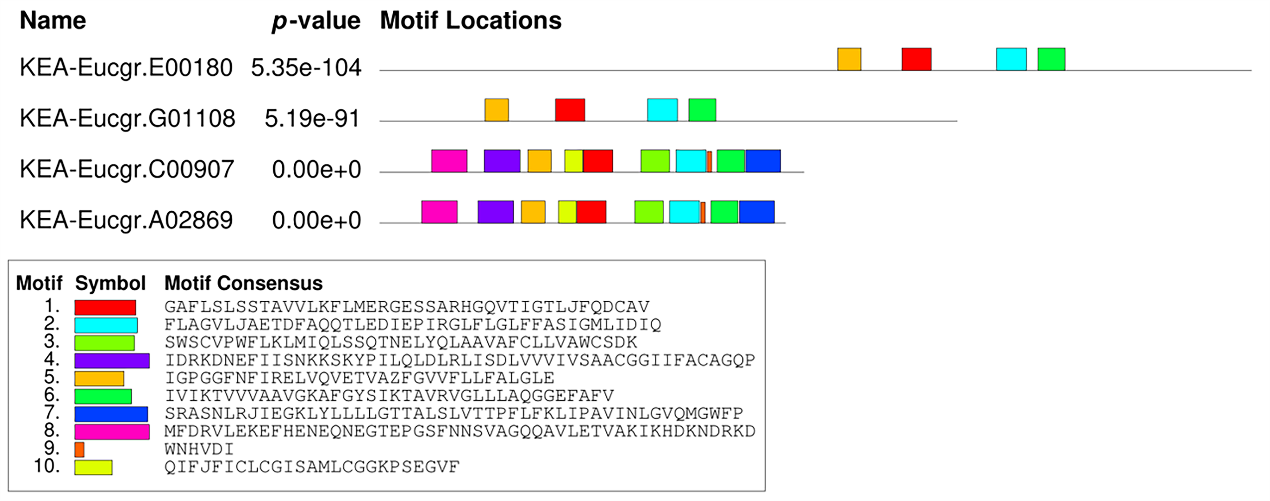

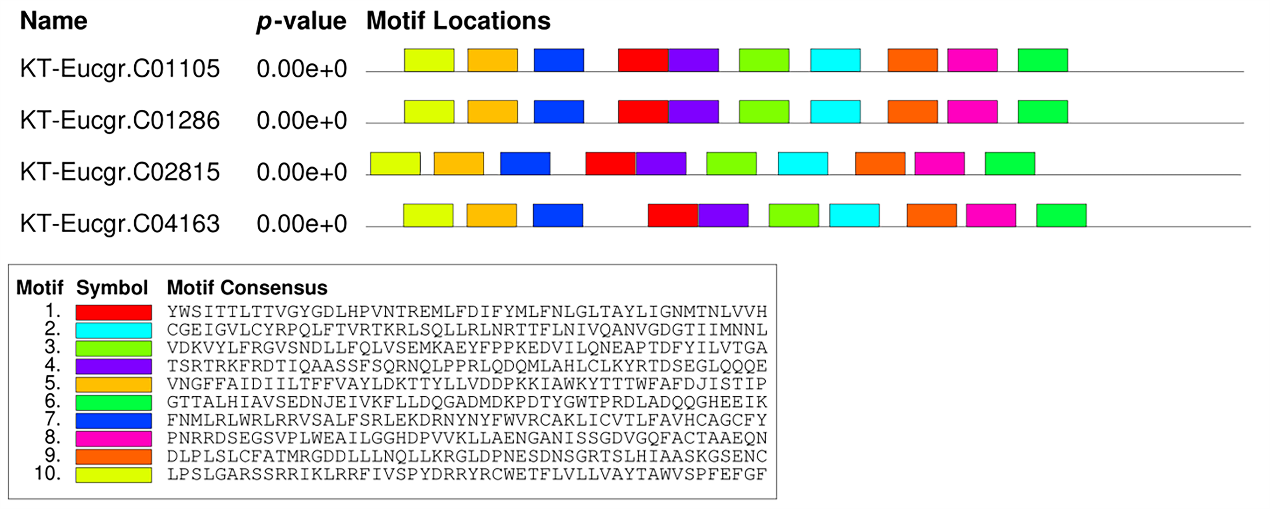


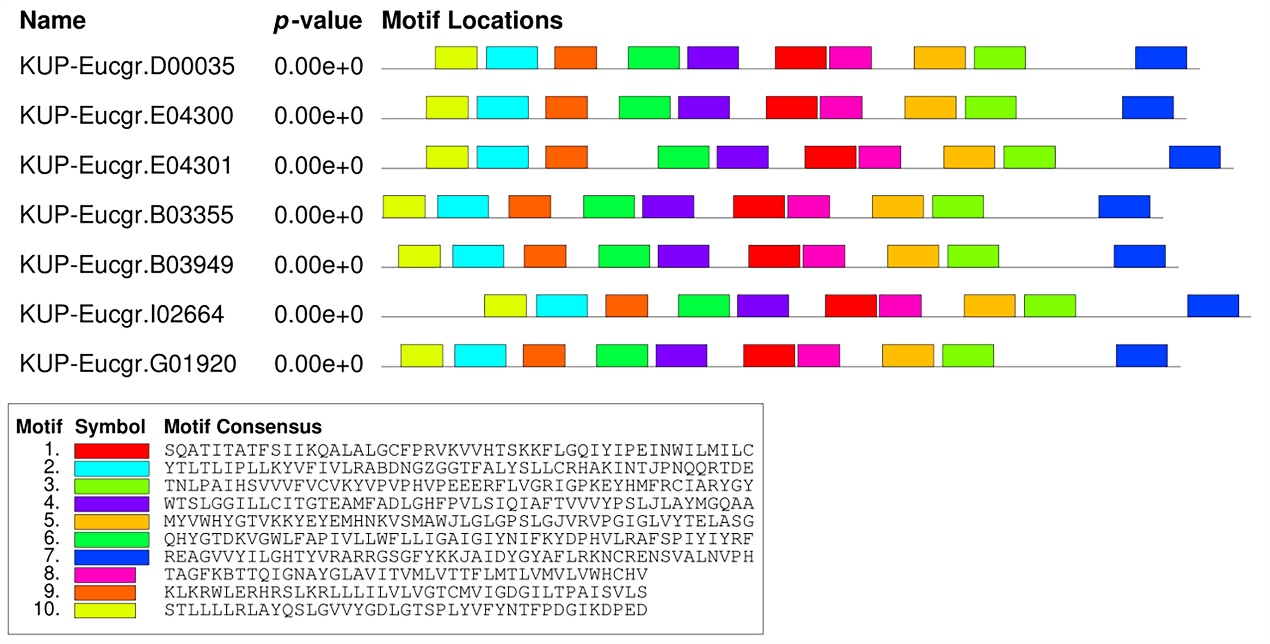


Supplementary Figure S30. The motif of Potassium transporters subfamily members (CHX, HAK, KEA, KT, KUP). The motif analyzed using MEME (https://meme-suite.org/meme/tools/meme).


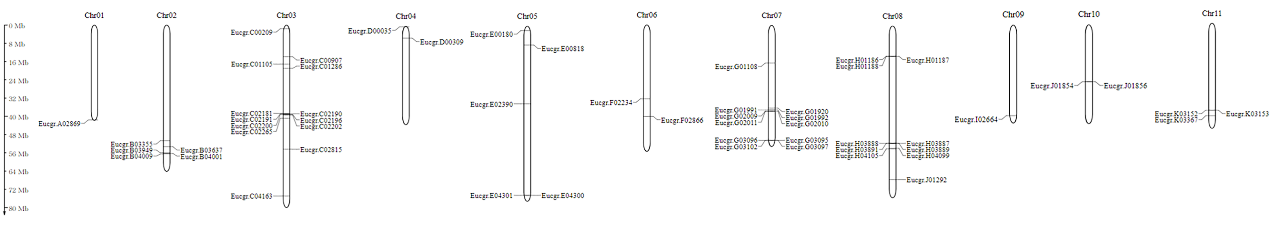


Supplementary Figure S31. The Chromosomal location of Potassium transporters family genes in *E.grandis* using MG2C (http://mg2c.iask.in/mg2c_v2.1/).


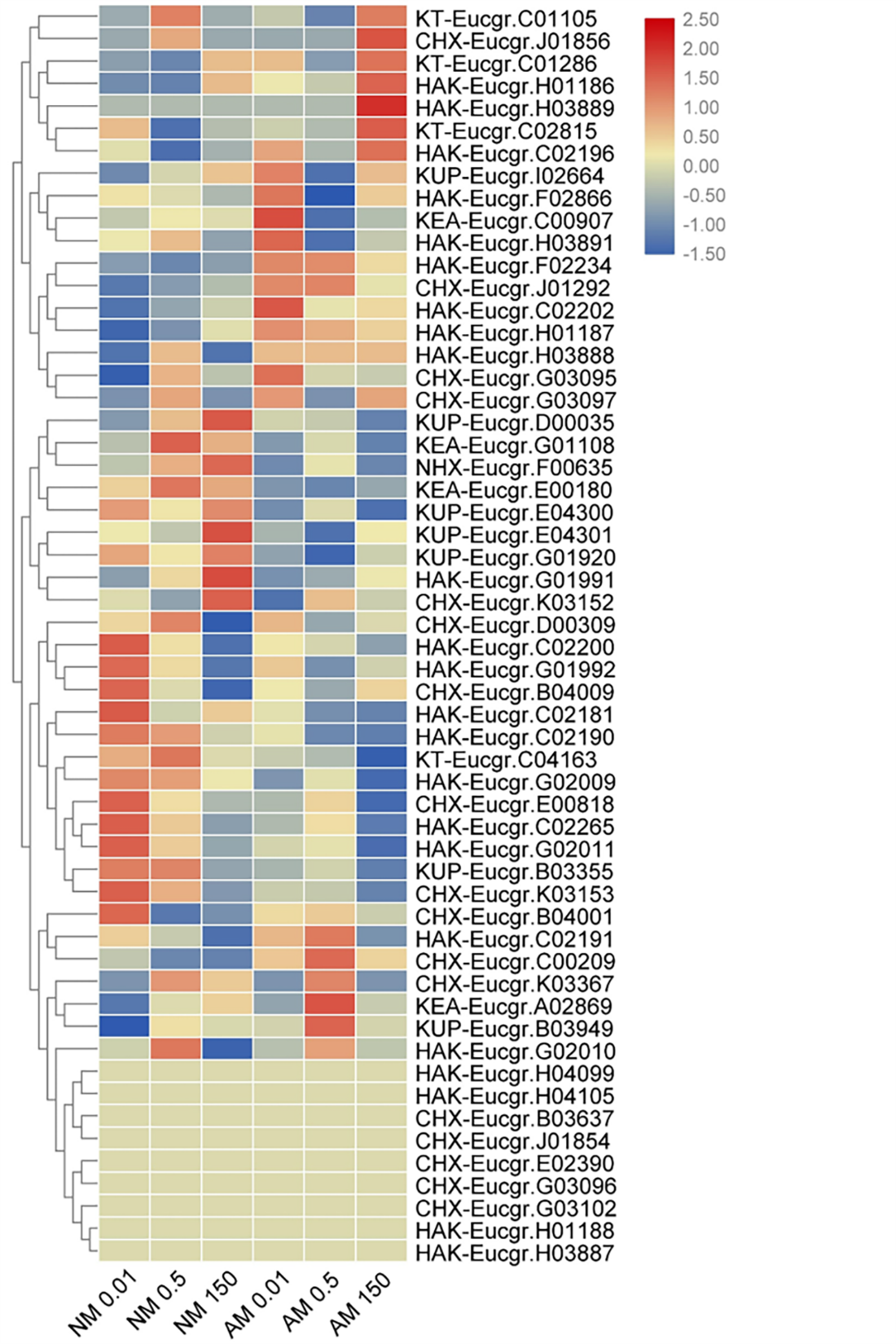


Supplementary Figure S32. Heatmap of Potassium transporters family members. Cluster analysis of transcriptional fold-changes of Potassium transporters gens in roots of non-mycorrhizal and mycorrhizal *E. grandis* exposed to 0.01, 0.5, or 150μM ZnCl_2_. The color scale indicates fold-changes of mRNAs. For each gene, the expression levels in non-mycorrhizal roots exposed to 0.5μM ZnCl_2_ were defined as 1, and the corresponding fold-changes under 0.01 and 150μM ZnCl_2_ were calculated.
